# Supplementary material for: Developing a machine learning model for accurate nucleoside hydrogels prediction based on descriptors
Source: Nat Commun. 2024 Mar 23;15:2603. doi: 10.1038/s41467-024-46866-9 (PMC10960799; doi:10.1038/s41467-024-46866-9)
Supplement: Supplementary file 1 — Supplementary Information [file 41467_2024_46866_MOESM1_ESM.pdf]

Supplementary Information

Developing a machine learning model for accurate nucleoside hydrogels prediction based on descriptors

Weiqi Li#, Yinghui Wen#, Kaichao Wang#, Zihan Ding, Lingfeng Wang, Qianming Chen, Liang Xie*, Hao Xu*, Hang Zhao*

State Key Laboratory of Oral Diseases, National Clinical Research Center for Oral Diseases, Research Unit of Oral Carcinogenesis and Management, Chinese Academy of Medical Sciences, West China Hospital of Stomatology, Sichuan University, Chengdu, Sichuan 610041, P. R. China.

# These authors have contributed equally to this work

* Correspondence and requests for materials should be addressed to:
Liang Xie
Email: lxie@scu.edu.cn
Hao Xu
Email: hao.xu@scu.edu.cn
Hang Zhao
Email: zhaohangahy@scu.edu.cn

Table of Contents

1. Supplementary Discussion..1
2. Supplementary Methods..5
3. Supplementary Figures..8
4. Supplementary Tables..40
5. Supplementary References..48

1. Supplementary Discussion

1.1 Key descriptors of the gelator properties

Features 1: nN $(CO)_2$, number of imides (-thio). In organic chemistry, an imide is a functional group consisting of two acyl groups bound to nitrogen. The compounds are structurally related to acid anhydrides, although imides are more resistant to hydrolysis. In terms of commercial applications, imides are best known as components of high-strength polymers, called polyimides. The effect of imides on hydrogel has been reported [1].
Feature 2: P_VSA_charge_4, P_VSA-like on partial charges, bin 4. The molecule partial charges were calculated by partial equalization of orbital electronegativity (PEOE). The PEOE is based on the calculation of the electronegativity for each atom of the molecule, which can be determined using a set of defined coefficients. These coefficients are defined only for some atom types (H, C, N, O, F, Cl, Br, I, S, P, Si, B, Be, Mg, Al) in specific orbital hybridizations. The effect of electronegativity on self-assembly has also been reported [2].
Feature 3: B09[O-O], Binary of O – O at topological distance 9. the presence/absence that reflects the occurrences of atom pairs of O and O at topological distance 9. To date, a correlation has not been found between this property and hydrogels.
Feature 4: H-052, H attached to C^{0}_{sp3} with 1X attached to next C. a structural feature originally proposed for the prediction of octanol-water partition coefficient ($\log P$) and molar refractivity (MR). The strong correlation between $\log$ P and gel ability has been widely reported [3-5].

1.2 The formation of dynamic borate diester bonds

Davis et al. found the vicinal diol group in G could form monoesters and diesters with H_3BO_3, thus introducing borate diesters into the hydrogel network to improve the gel-forming[6]. Therefore, we speculated that the formation of dynamic borate diester bonds contributed to form stable and self-healing hydrogels in the absence of cations. The tube-inversion test results show that **6** and **8** couldn't form hydrogels in the presence of the cations of Na^+ or K^+ (Supplementary Fig. 14), suggesting cations couldn't help the process of self-assembling into hydrogels. Furthermore, they failed to form hydrogels in the presence of H_3BO_3 but could successfully construct stable cation-independent hydrogels in the solution of H_3BO_3 and Tris which could be explained by the fact that borate diesters are stable in alkaline solution but easily hydrolyzed in acidic solution. The presence of borate diester bonds was explored by ^{11}B Nuclear magnetic resonance (NMR) and Alizarin Red S (ARS) experiments. ^{11}B NMR results show that H_3BO_3 display a signal at 22.72 ppm while hydrogels display two peaks at 0 – 15 ppm (Fig. 6a, Supplementary Fig. 28), and the ARS experiments show that the fluorescence gradually decreases from H_3BO_3 to

hydrogels (Fig. 6b, Supplementary Fig. 29), both suggesting the presence of the borate diester bonds.

1.3 Thioflavin T (ThT) assay

ThT functions as a molecular chaperone by end stacking on G-quartets, and fluorescence could be observed only if the G-quartets exist [7]. The ThT assay was performed to investigate the presence of G-quartets in the hydrogels. Fig. 6c and Supplementary Fig. 30 show that there are no fluorescent signals in 8AG-T/Na^+/K^+ and 8OHG-T/Na^+/K^+ hydrogels, while G-K^+ hydrogel exhibit strong fluorescent signals, demonstrating that G-quartets exist in G-K^+ hydrogel but not in 8AG-T/ Na^+/K^+ and 8OHG-T/Na^+/K^+ hydrogels.

1.4 The single crystal of 6

Instead of forming a G-quartet similar to G, the attendance of 8-NH_2 in **6** makes it construct a ribbon-like base-pair pattern along the *b*-axis. In addition, viewing along the *b* axis, it is observed that two DMSO molecules, DMSO1 and DMSO2, connect the adjacent molecules of **6** (Fig. 6h, Supplementary Fig. 36). For DMSO1, with a bending angle of 81.99°, the O11 links the surrounding **6** by forming HBs with O3' and O5' in sugar parts (O3'-H3'···O11 and O5'-H5'···O11) and N2 (N2-H2A···O11) in base parts (Supplementary Fig. 37-38). Meanwhile, the weak C-H···O HBs constructed by the C13 of DMSO1 and the O2' of **6** also contribute to the connection of adjacent **6**. DMSO2 connected two adjacent base-pair layers by forming two HBs (O2'-H2'···O15 and C16-H16B···O5'). Finally, to further understand the holistic intermolecular HBs in the crystal structures of **6**, Hirshfeld analysis was also performed, and the surfaces were mapped over its d_{norm}, shape index, and curvedness (Supplementary Fig. 39). Quantitatively, the nearly identical percentages of H···H (37.9%) and O···H/H···O (32.7%) interactions indicate the contributions of the O11 and O15 of two DMSO molecules to the connection of **6** (Supplementary Fig. 40-41). Moreover, in the 2D fingerprint plot of **6**, the O···H/H···O and N···H/H···N interactions appear as a pair of spikes at the bottom left of the plot (i.e., short di and de), indicating the existence of cyclic HBs.

2. Supplementary Methods

2.1 Details of model construction

To obtain such a dataset and construct the prediction model, all the published nucleoside derivatives, and the information on whether they have the hydrogel-forming ability were collected by systematic literature review, and 71 molecules were included. To unify the molecular structures, the ChemDraw software (Version 20.0) was utilized to redraw the structures of 71 nucleoside derivatives. These nucleoside derivatives were then divided into two groups, gelator (n=38) and non-gelator (n=33) groups, which was based on whether they have the hydrogel-forming ability (Supplementary Data 1). For the subsequent ML models constructed, all the molecules were converted to the standard SMILES (Simplified Molecular Input Line Entry System). Then the dataset including the structures of 71 nucleoside derivatives and the information on whether they have the hydrogel-forming ability was established (Supplementary Data 2).

A total of 5566 molecular descriptors were calculated for each nucleoside derivative by alvaDesc (Supplementary Data 3). After removing the 1491 descriptors with missing values that were not suitable for this study, we initially obtained 4175 descriptors for the 71 nucleoside derivatives (Supplementary Fig. 1). Subsequently, a three-step feature selection was utilized based on the 4175 descriptors to avoid overfitting and improve the model accuracy. The flow chart of the feature selection was illustrated in Fig. 2a. Firstly, the rank-sum test was used to find the descriptors have significant differences (P<0.05) between gelator (n=38) and non-gelator (n=33) group, 144 descriptors were obtained (Fig. 2b, Supplementary Fig. 2). Secondly, exclude one of the pairs of descriptors with correlation coefficient higher than 0.8 (Rho>0.8) with Spearman correlation to avoid the collinearity. After this step, 40 descriptors were kept for subsequent model training (Fig. 2c, Supplementary Fig. 4). To visually demonstrate whether the descriptors after feature selection could representatively distinguish the gelator and non-gelator groups, the three-dimensional (3D) principal component analysis (PCA) were plotted (n for descriptors= 4175, Fig. 2d; n for descriptors= 144, Supplementary Fig. 3; n for descriptors= 40, Fig. 2e). Finally, ML algorithm-based recursive feature elimination (RFE) was used to obtain the optimal combination of descriptors that maximizes model performance. Four commonly used ML algorithms were utilized to construct prediction models: LR, decision tree (DT), RF, and extreme gradient boosting (XGBoost).

Taken together, to construct the prediction models comprehensively, different mathematical representations of molecules based on descriptors were used to build prediction models with four ML algorithms, details for the built models were shown in Supplementary Table 1. The performances of all these models were assessed by five evaluation indexes, including test accuracy, area under the curve (AUC), precision, recall and F1 score (Supplementary Methods 2.2

Details of model parameters). And in this study, test accuracy and AUC were mainly focused on, and the results of precision, recall, and F1 score were used as auxiliary indicators. Fivefold stratified cross-validation which was performed 10 times independently was applied in hyperparameter optimization, recursive feature elimination (RFE), and calculation for evaluation indexes.

2.2 Details of model parameters

The optimal model was determined with reference to the results of accuracy and AUC, and with attention to parameters including recall, F1 score and precision.

A true positive (*TP*) is an outcome where the model correctly predicts the positive class. Similarly, a true negative (*TN*) is an outcome where the model correctly predicts the negative class. A false positive (*FP*) is an outcome where the model incorrectly predicts the positive class. And a false negative (*FN*) is an outcome where the model incorrectly predicts the negative class.

Precision: Denotes the percentage of samples with positive predictions that are truly positive. In our study, it means the proportion for the true gelators of we predicted gelators.

$$\text{Precision} = \frac{TP}{TP+FP} \tag{1}$$

Recall: Denotes the percentage of samples that are truly positive and the number of samples that are predicted to be positive. In our study, it means the proportion for accurately predicted gelators of all gelators.

$$\text{Recall} = \frac{TP}{TP+FN} \tag{2}$$

F1 score: It can be interpreted as a harmonic mean of the precision and recall. The higher the summed average of precision and recall is, the better the model performance is.

$$\text{F1 score} = \frac{2TP}{2TP+FP+FN} \tag{3}$$

Accuracy: The percentage of total predictions that were correct. In our study, it means the proportion of accurately predicted gelators and nongelators in nucleoside derivatives.

$$\text{Accuracy} = \frac{TP+TN}{TP+TN+FP+FN} \tag{4}$$

AUC: Denotes the area under the ROC curve. When a gelator and a non-gelator is randomly picked, the probability of the gelator ranking in front of the non-gelator according to the calculated score is the AUC value. The larger the AUC value is, the better the predicted model performs.

2.3 Details of cluster analysis (K-means) for test set

We reduced dimensionality by PCA for 4175 molecular descriptors of 71 nucleoside derivatives. Using k-means, 71 nucleoside derivatives were

clustered (Supplementary Fig. 5). The K value was determined based on the elbow’s method and silhouette score (K=4, Supplementary Fig. 6). To ensure that the test set covered the same areas as the training set, we conducted stratified sampling based on clustering and gelling results, dividing nucleoside derivatives into 80% as training set (n=56) and 20% as test set (n=15) (Supplementary Fig. 7). We use 5-fold cross validation to train and hyperparameter the model on the training set and evaluate the model's generalization ability on the additional test set.

3. Supplementary Figures

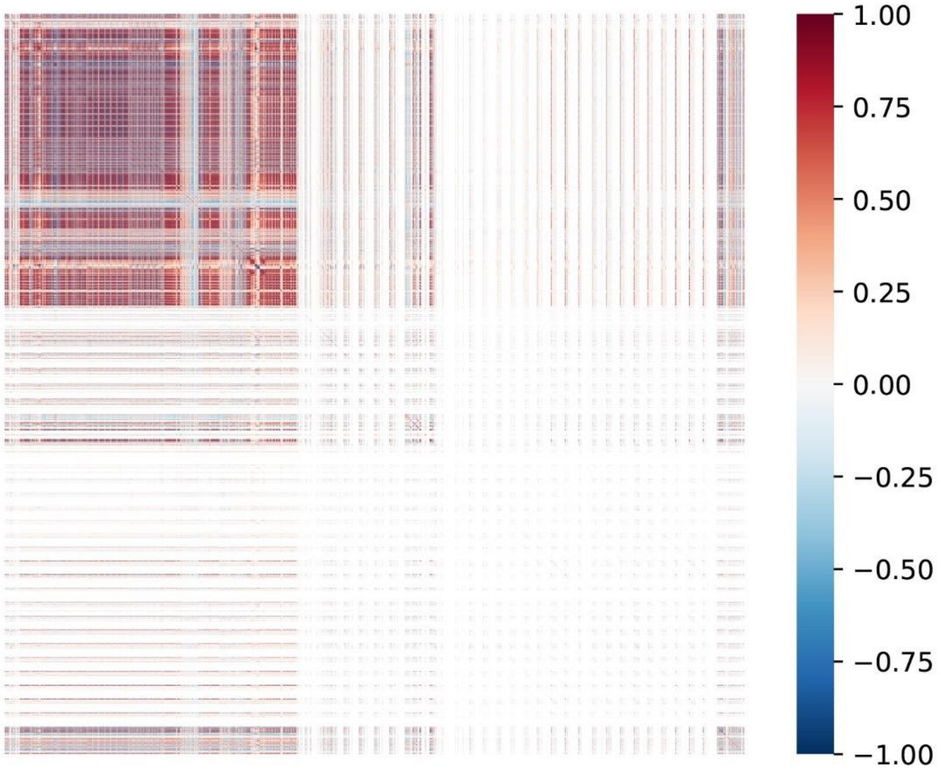

Supplementary Fig. 1. 4175 descriptors correlation heatmaps. Presence of a large number of descriptors with high relevance (correlation >0.80).

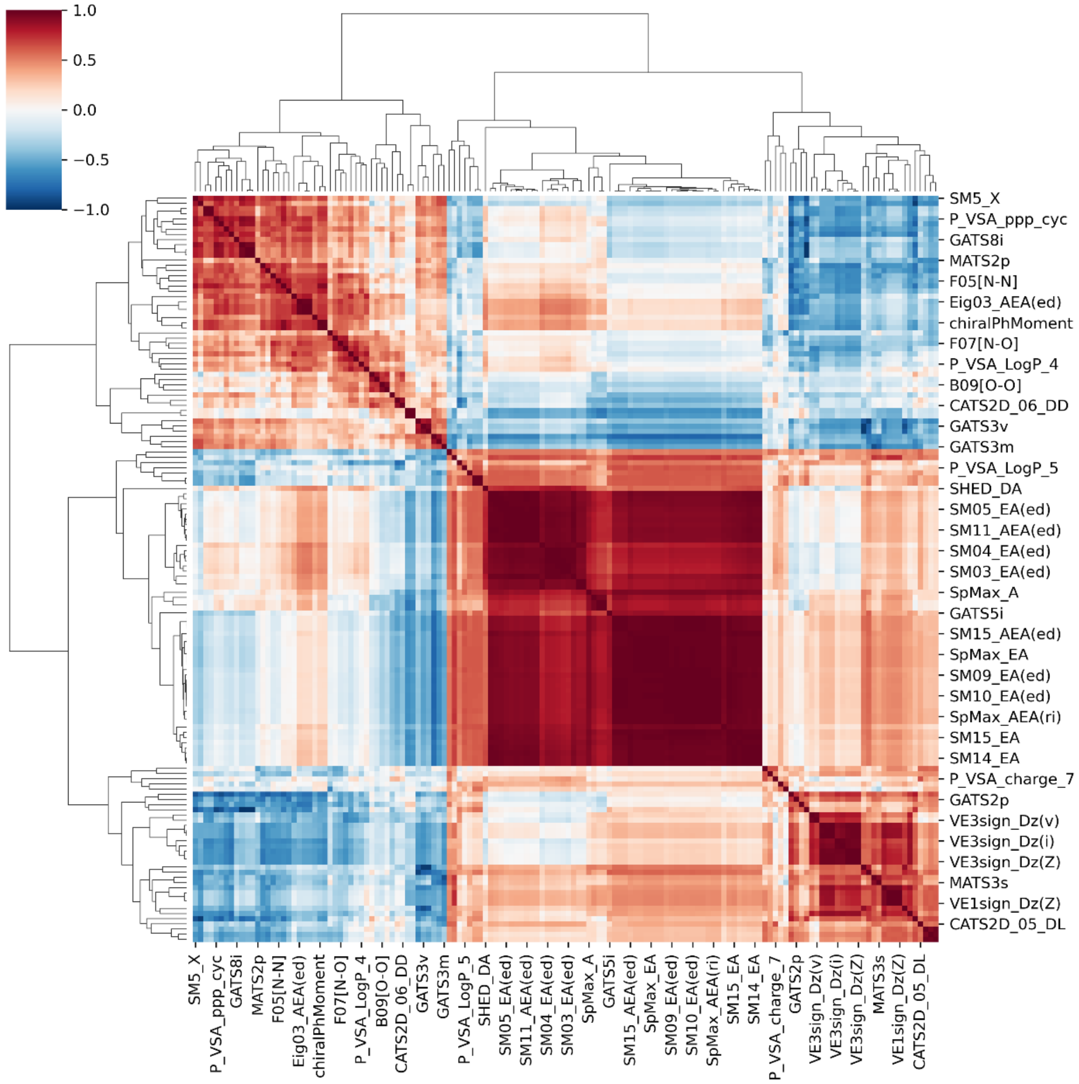

Supplementary Fig. 2. 144 descriptors correlation heatmaps after the rank-sum test selection (*P*<0.05). Still presence of a large number of descriptors with high relevance (correlation >0.80).

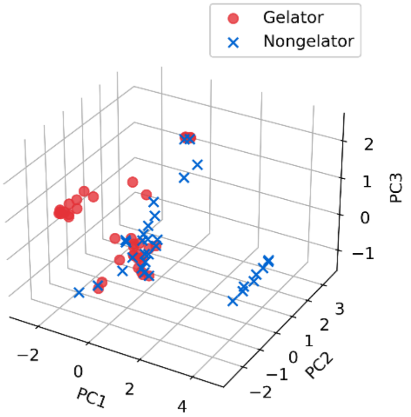

Supplementary Fig. 3. 3D PCA of 144 descriptors. The principal component analysis (PCA) of the 144 descriptors shows that the gelator and non-gelator groups cannot be well distinguished.

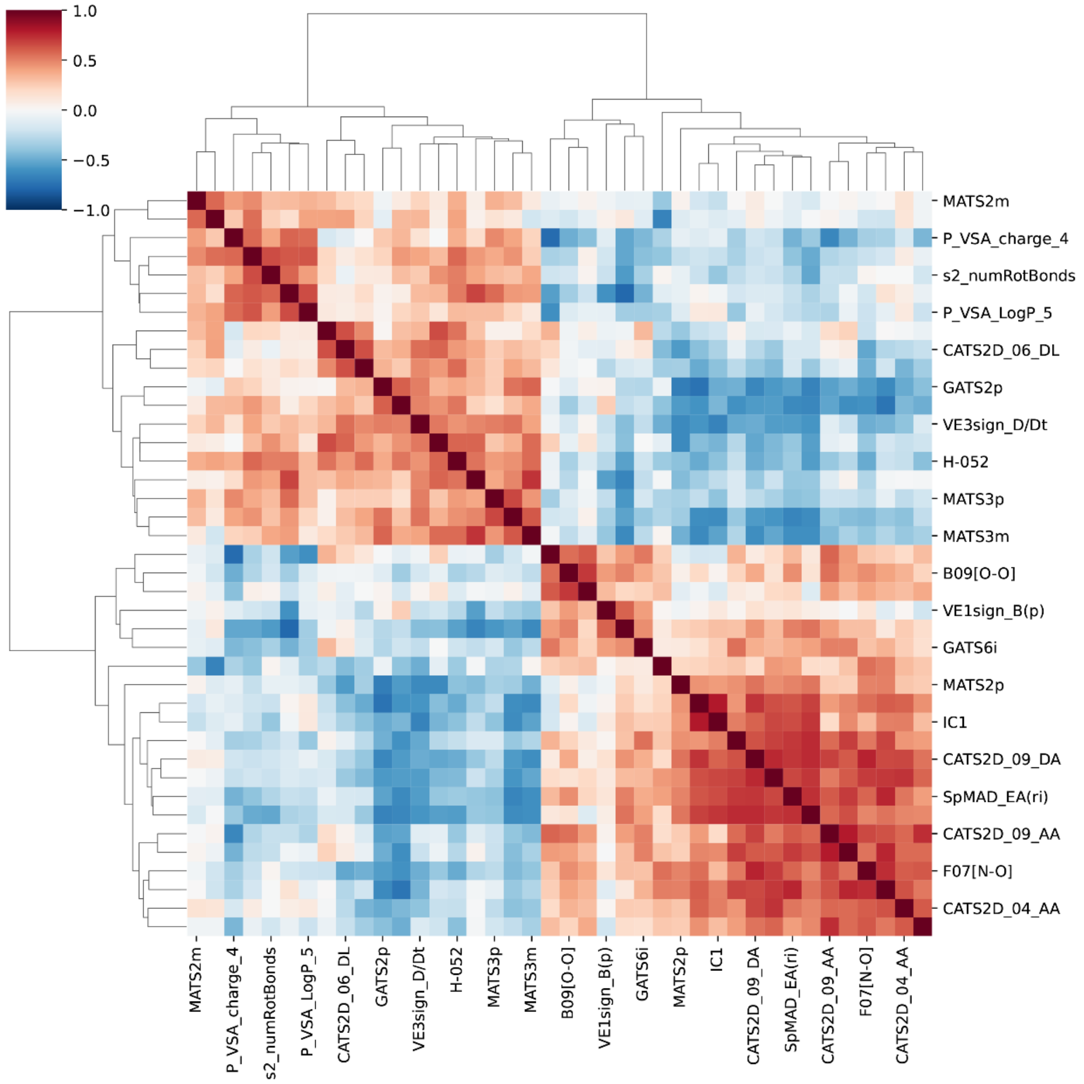

Supplementary Fig. 4. Details of 40 descriptors correlation heatmaps. All correlations between descriptors are less than 0.80 after feature selection.

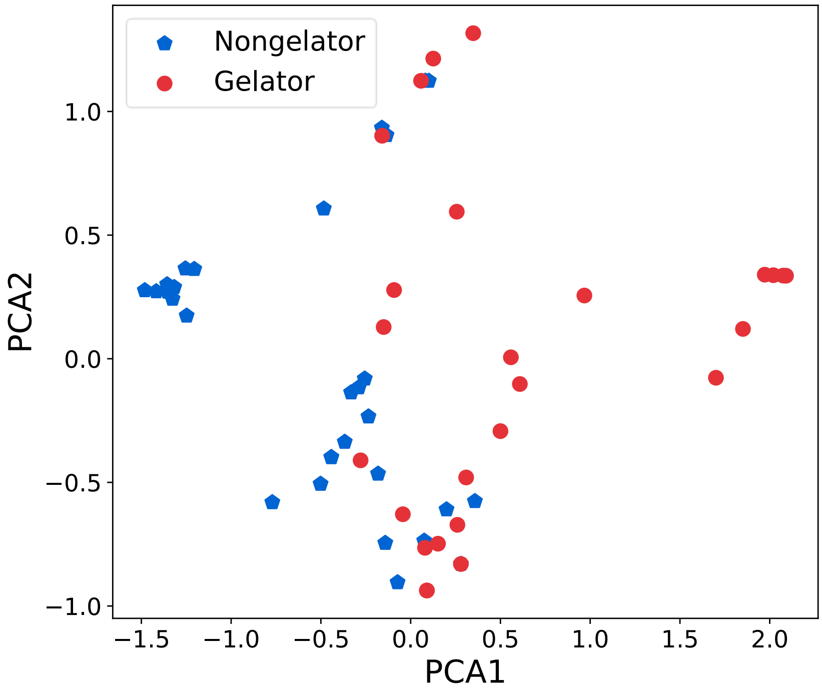

Supplementary Fig. 5. Results of principal component analysis (PCA)

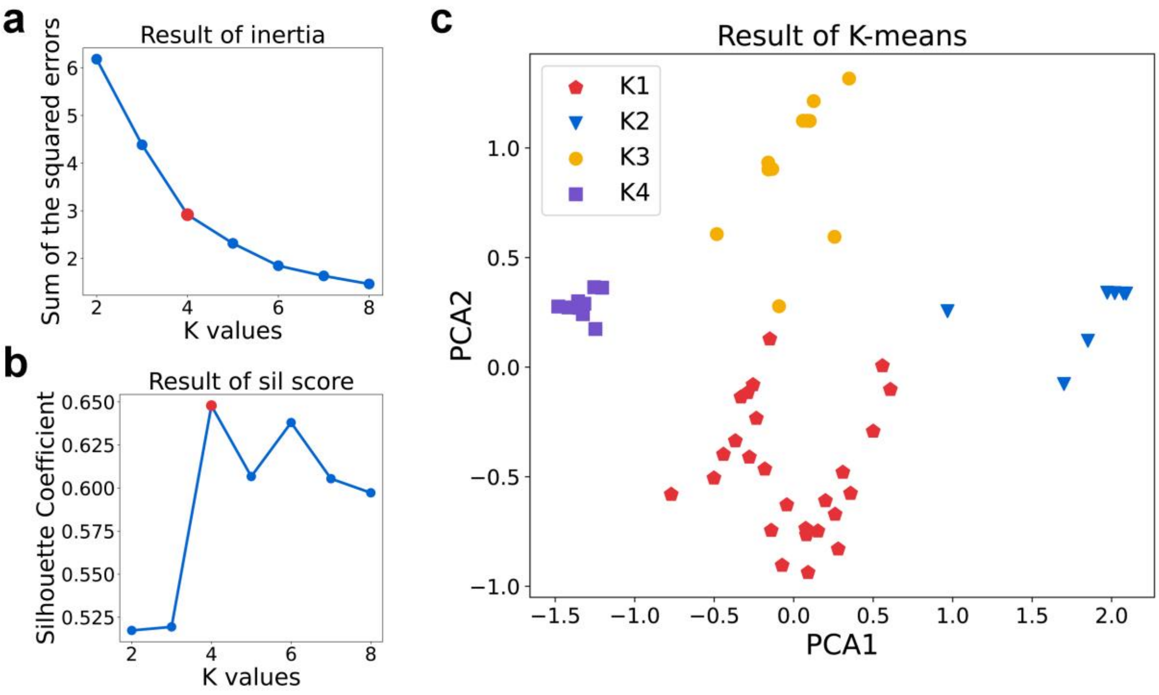

Supplementary Fig. 6. Results of cluster analysis of K-means. a. result of inertia, b. result of silhouette score, c. result of K-means (K=4).

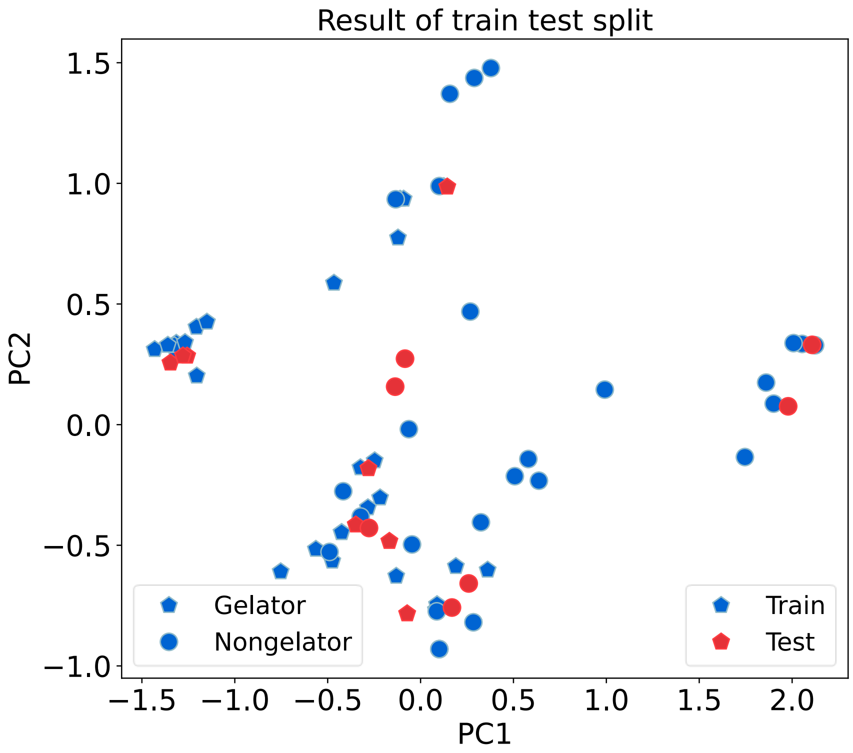

Supplementary Fig. 7. The distribution of training set and test set

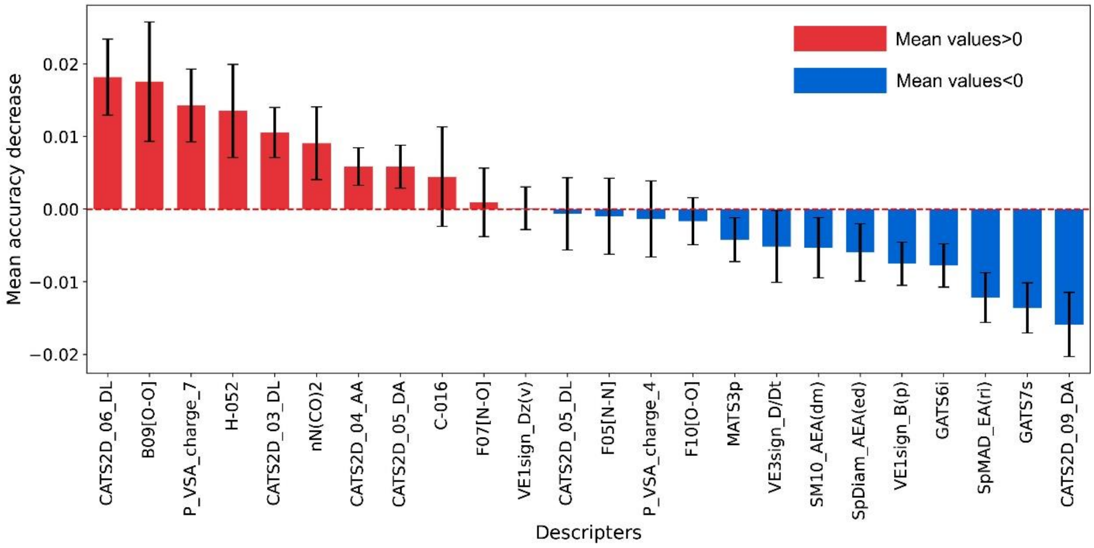

Supplementary Fig. 8. The feature importance of 24 descriptors for logistic regression based on the permutation feature importance. Data are mean values ± standard error of the mean (SEM).

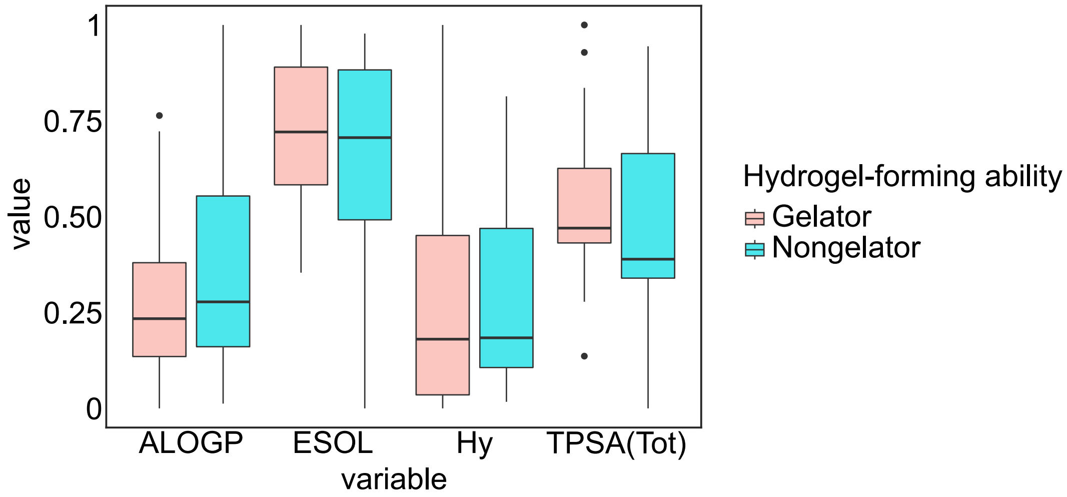

Supplementary Fig. 9. A grouped box plot of 4 descriptors which express chemical properties. These descriptors may be relevant to hydrolgel-forming ability of nucleoside derivatives (hydrophilicity, Hy; topological polar surface area, TPSA; octanol-water partition coefficient, log *P*; and solubility, ESOL).

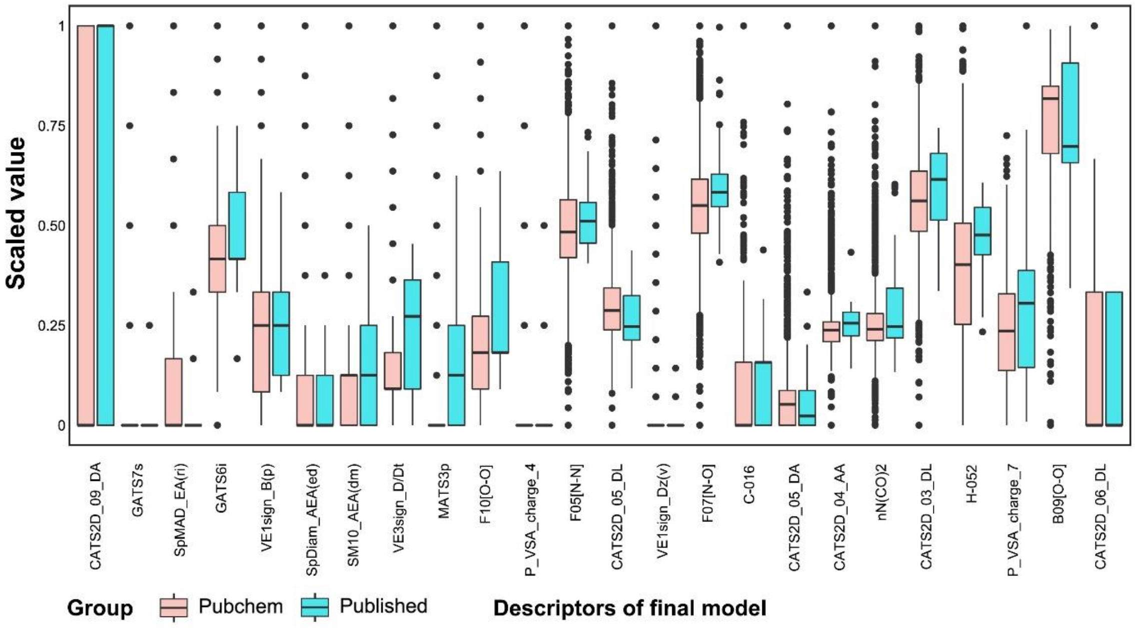

Supplementary Fig. 10. A grouped box plot of 24 molecular descriptors (optimal model) for nucleoside derivatives. Nucleoside derivatives are divided into two groups, including nucleoside derivatives from PubChem dataset (Pubchem group, n=7257) and all published nucleoside gelators and nongelators (Published group, n=71).

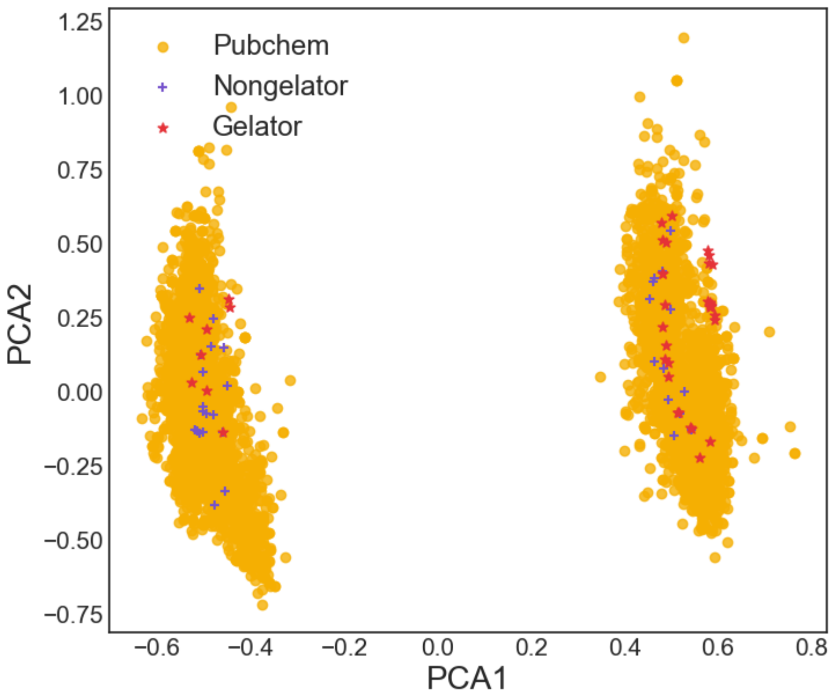

Supplementary Fig. 11. 2D-PCA results of 24 features grouped by nucleoside derivatives. Including PubChem dataset (Pubchem group, n=7257), published gelators (Gelator group, n=38) and published nongelators (Nongelator group, n=33).

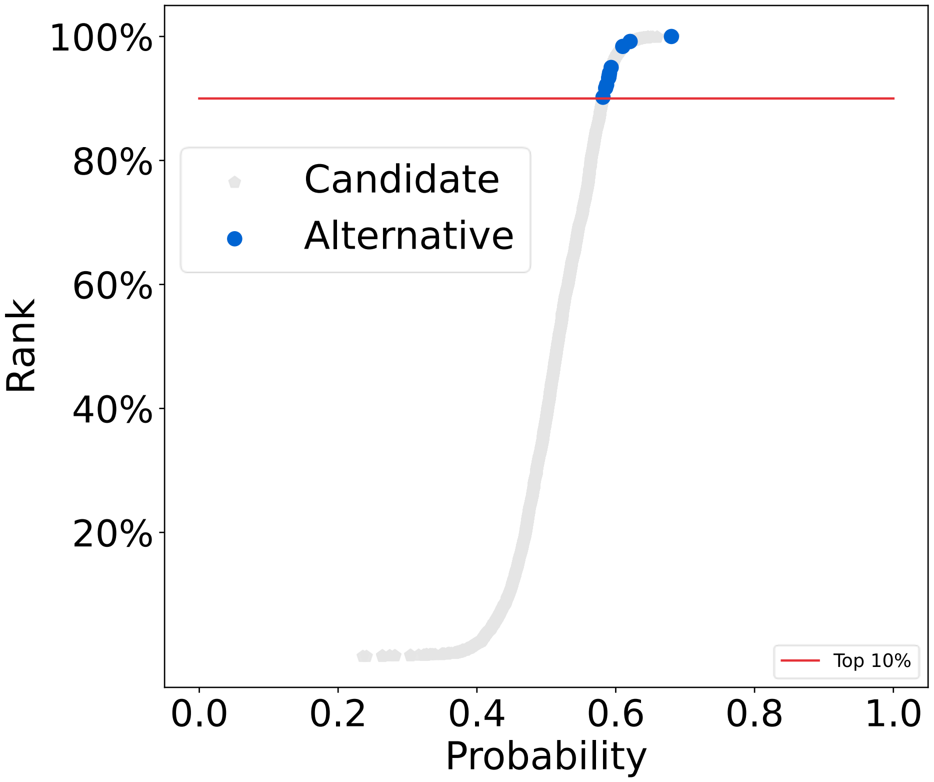

Supplementary Fig. 12. The 12 chosen alternative nucleoside derivatives based on gelator probabilities of top 10%. The x-axis representing the hydrogel-forming probability and the y-axis representing the ranking of the probabilities from smallest to largest.

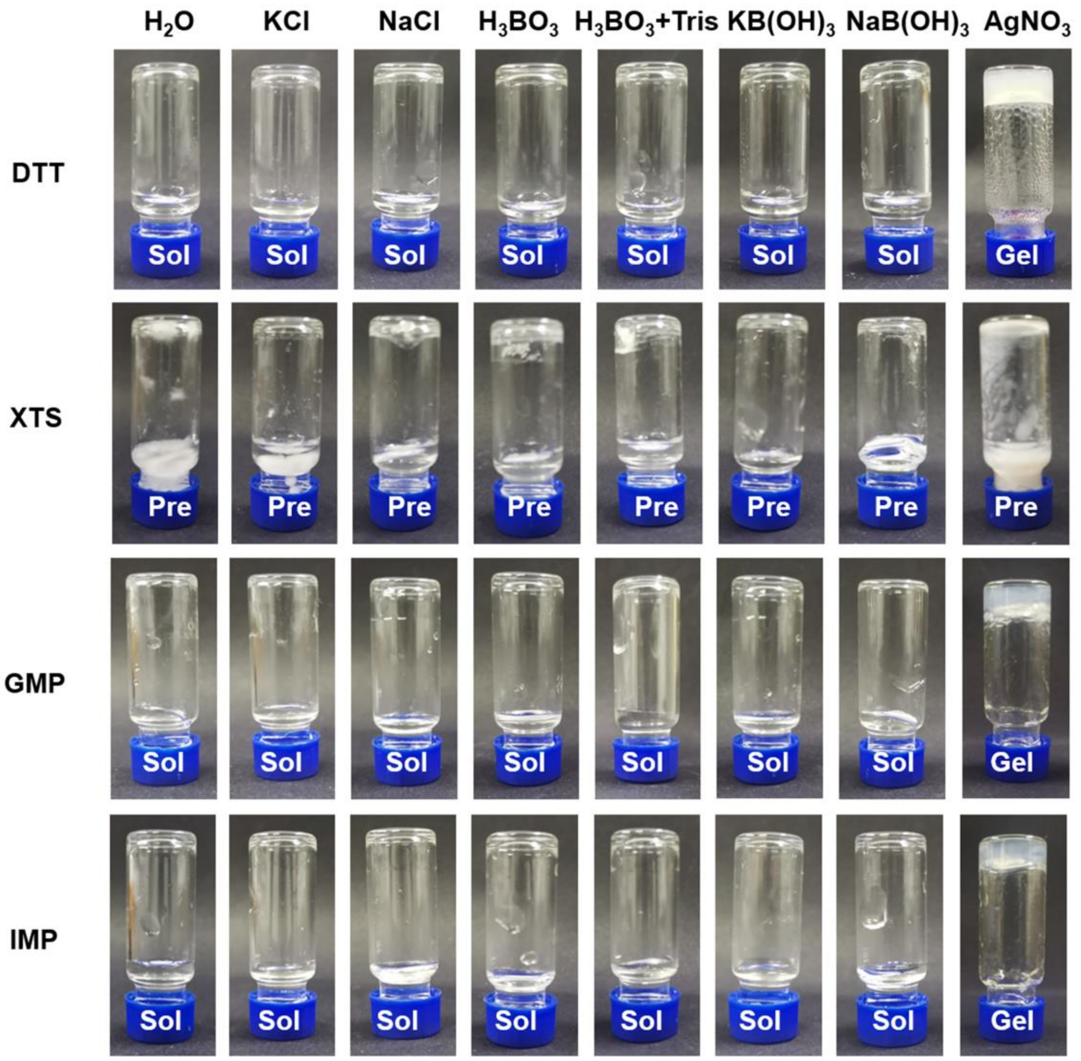

Supplementary Fig. 13. Photographs of hydrogels or samples assembled from nucleoside derivatives in different solutions. Sol: solution. Pre: precipitate.

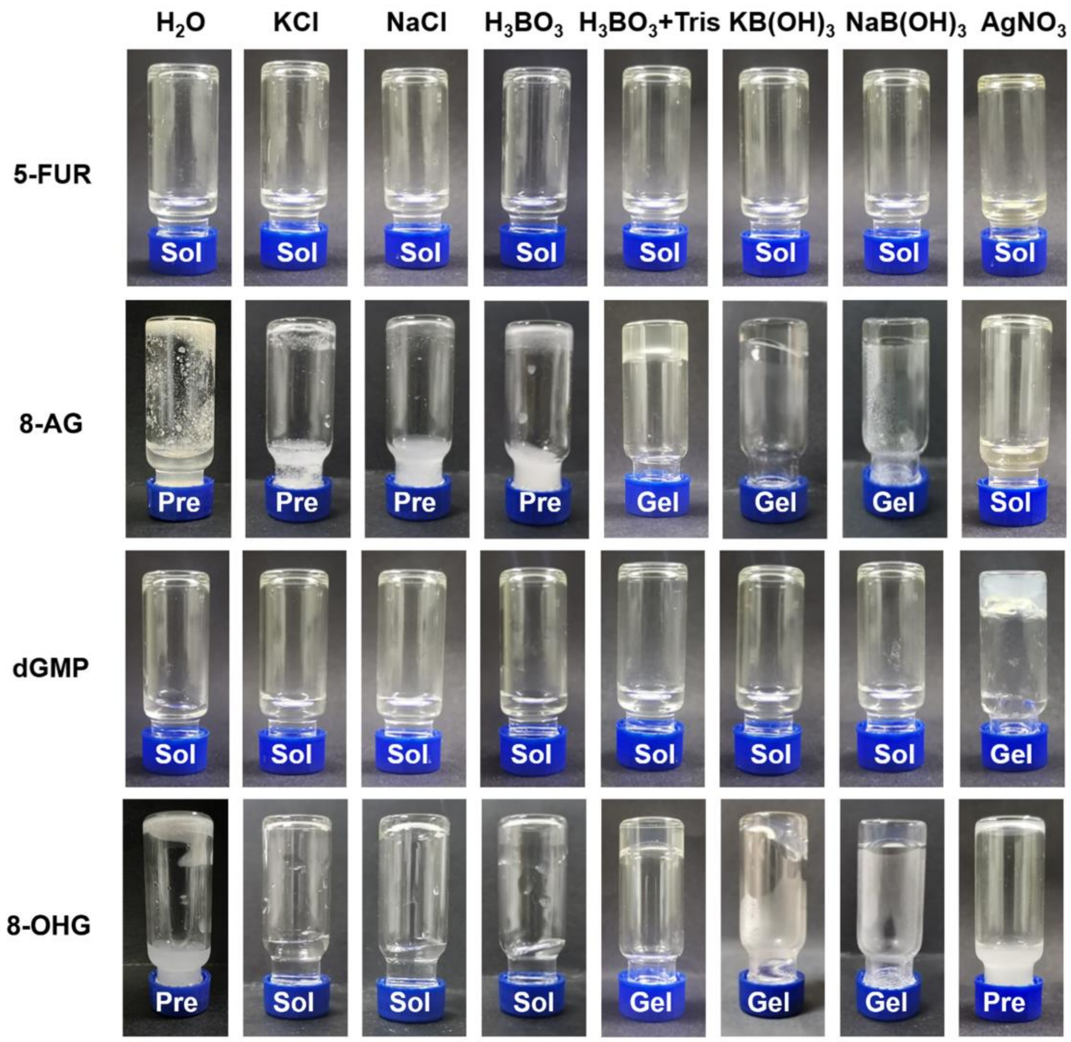

Supplementary Fig. 14. Photographs of hydrogels or samples assembled from nucleoside derivatives in different solutions. Sol: solution. Pre: precipitate.

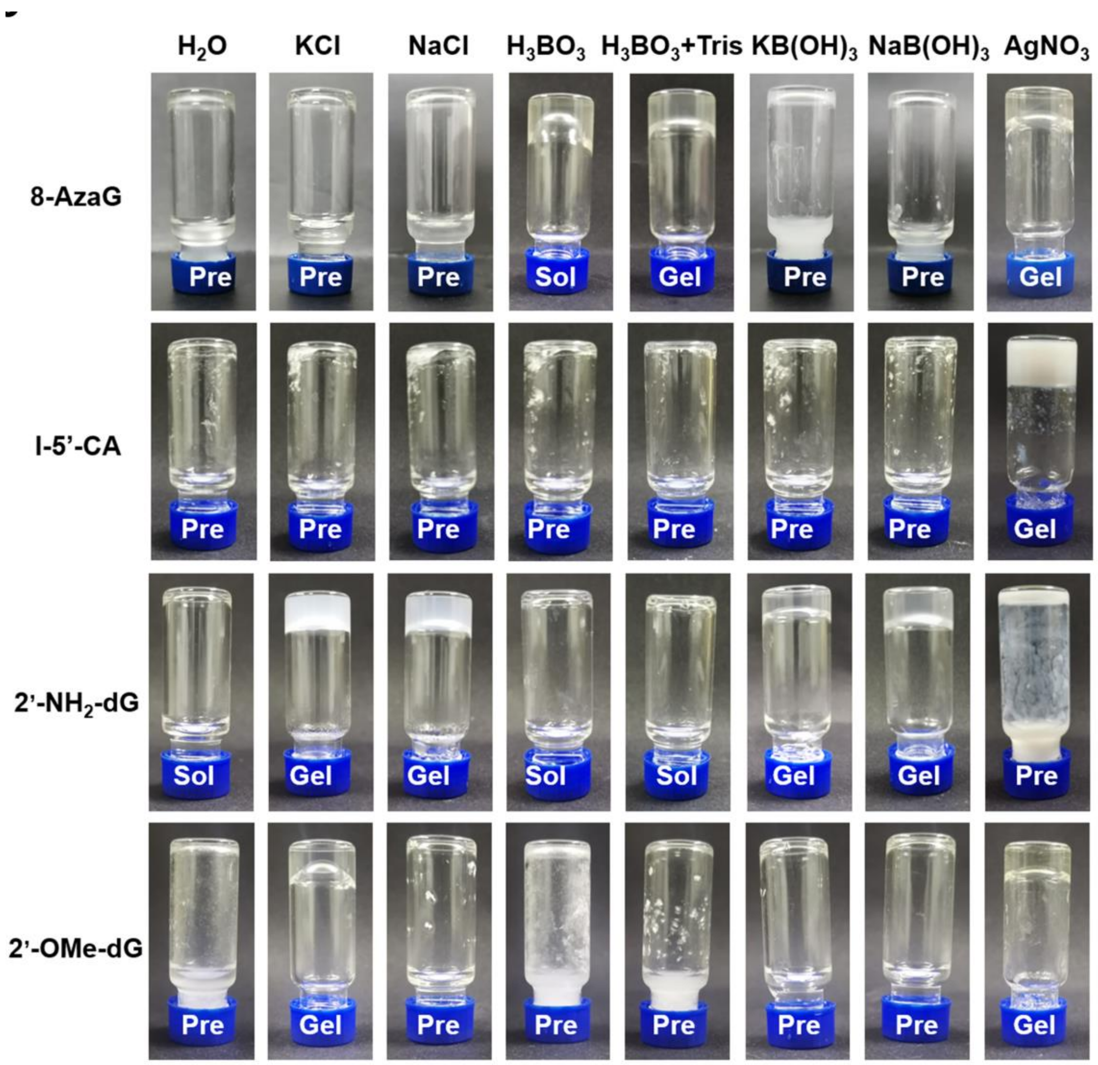

Supplementary Fig. 15. Photographs of hydrogels or samples assembled from nucleoside derivatives in different solutions. Sol: solution. Pre: precipitate.

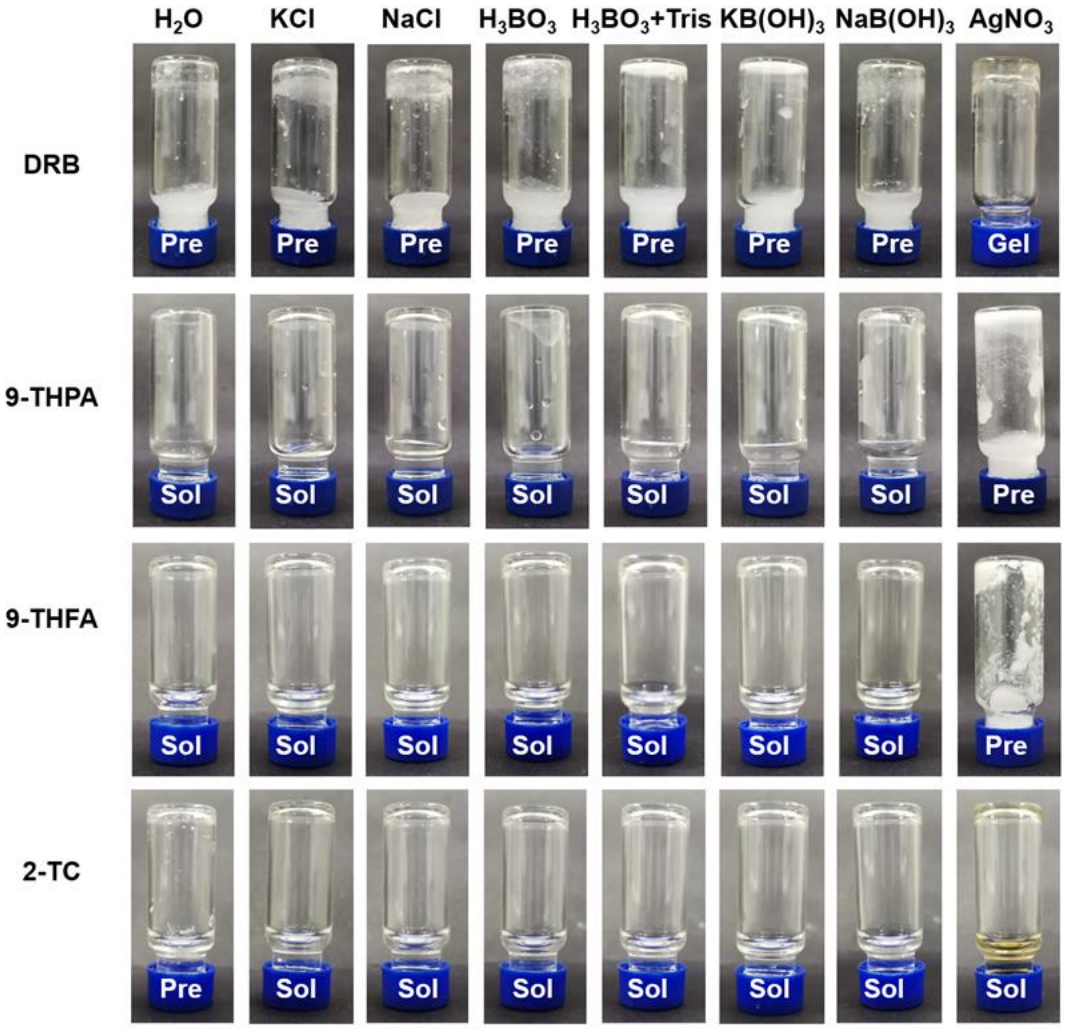

Supplementary Fig. 16. Photographs of hydrogels or samples assembled from nucleoside derivatives in different solutions. Sol: solution. Pre: precipitate.

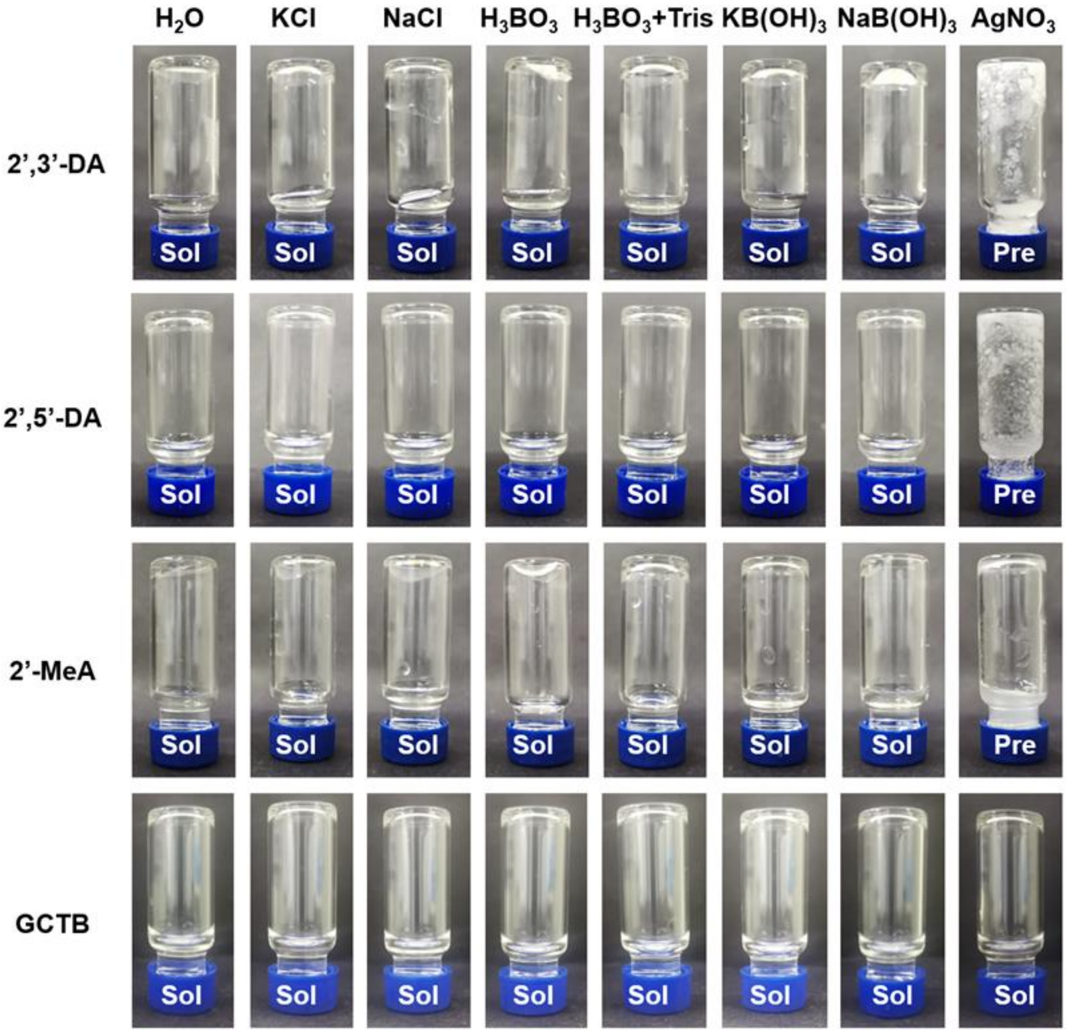

Supplementary Fig. 17. Photographs of hydrogels or samples assembled from nucleoside derivatives in different solutions. Sol: solution. Pre: precipitate.

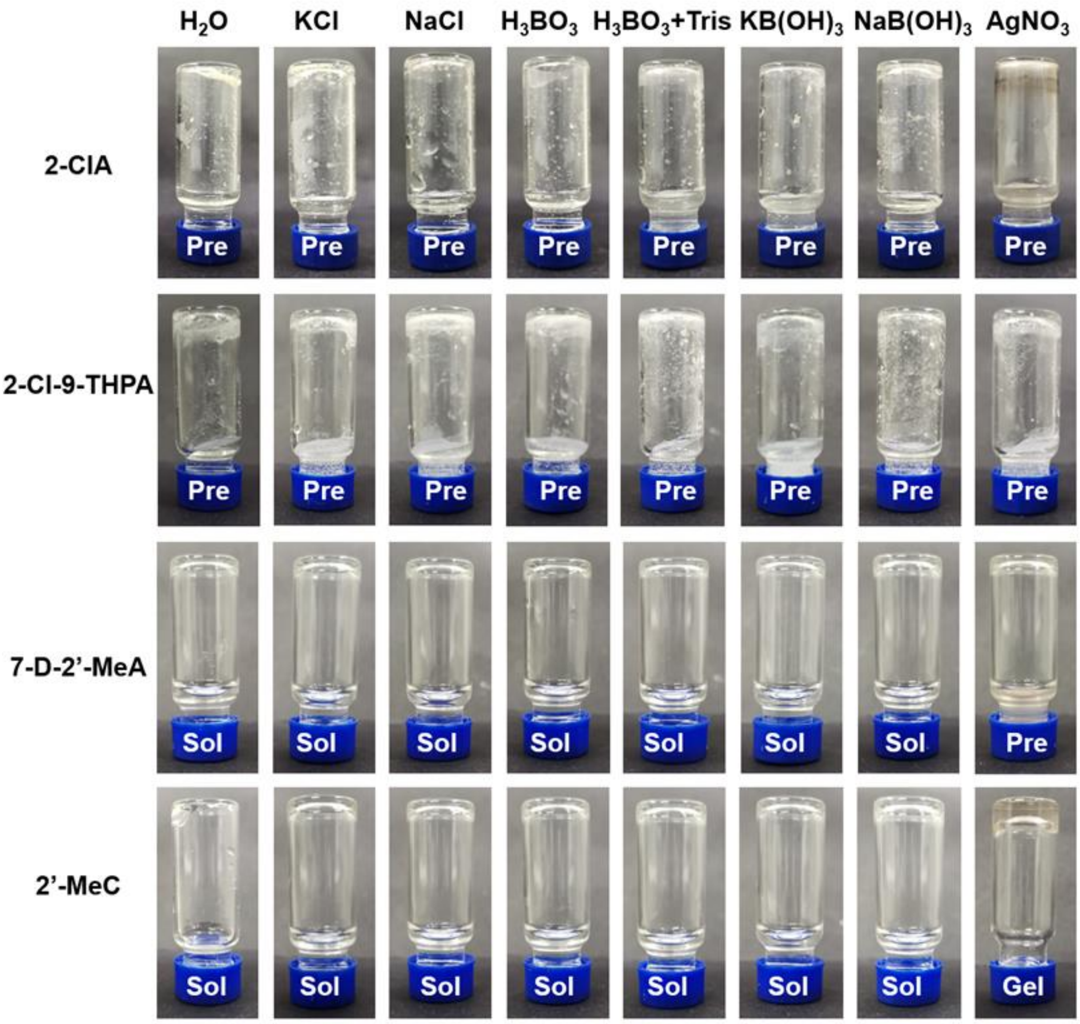

Supplementary Fig. 18. Photographs of hydrogels or samples assembled from nucleoside derivatives in different solutions. Sol: solution. Pre: precipitate.

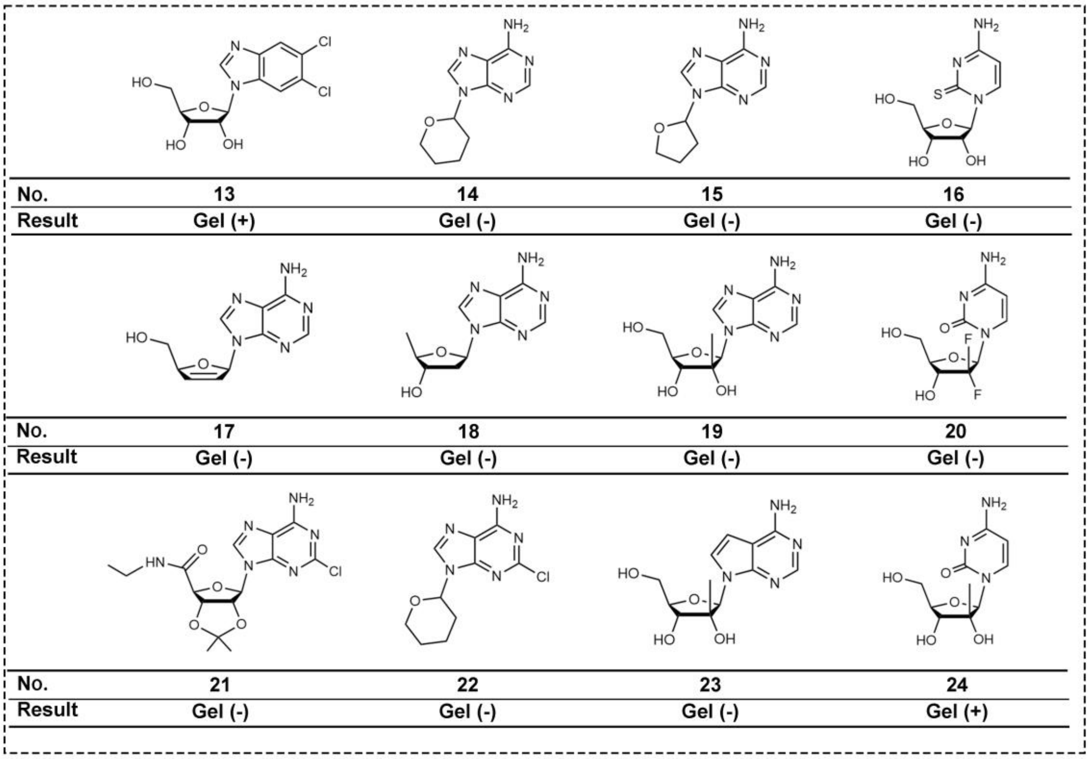

Supplementary Fig. 19. 12 nucleoside derivatives with low probability of hydrogel-forming ability were selected. The result shows 10 nucleoside derivatives (**14**-**23**) formed hydrogels, while the two others (**13** and **24**) did not. **13**, 5,6-Dichlorobenzimidazole riboside, DRB; **14**, 9-(2-tetrahydropyranyl)adenine, 9-THPA; **15**, 9-(2-tetrahydrofuryl)adenine, 9-THFA; **16**, 2-thiocytidine, 2-TC; **17**, 2',3'-dideoxy-2'3'-didehydroadenosine, 2',3'-DA; **18**, 2',5'-dideoxyadenosine, 2',5'-DA; **19**, 2'-C-methyladenosine, 2'-MeA; **20**, gemcitabine, GCTB; **21**, 2-chloro-2',3'-O-isopropylideneadenosine-5'-N-ethylcarboxamide, 2-ClA; **22**, 2-chloro-9-(2-tetrahydropyranyl)adenine, 2-Cl-9-THPA; **23**, 7-deaza-2'-C-methyladenosine, 7-D-2'-MeA; **24**, 2'-C-methylcytidine, 2'-MeC**.**

G

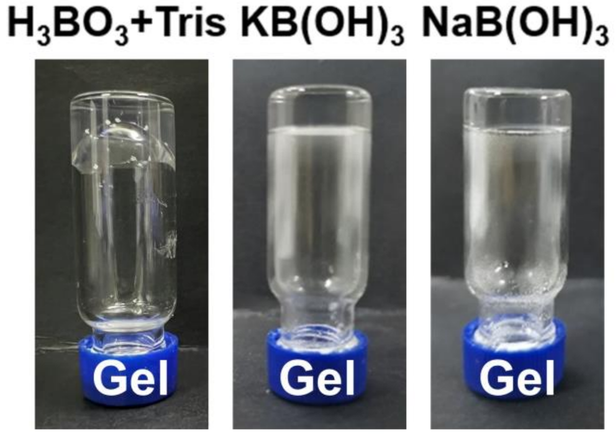

Supplementary Fig. 20. Photographs of hydrogels assembled from G in different solutions.

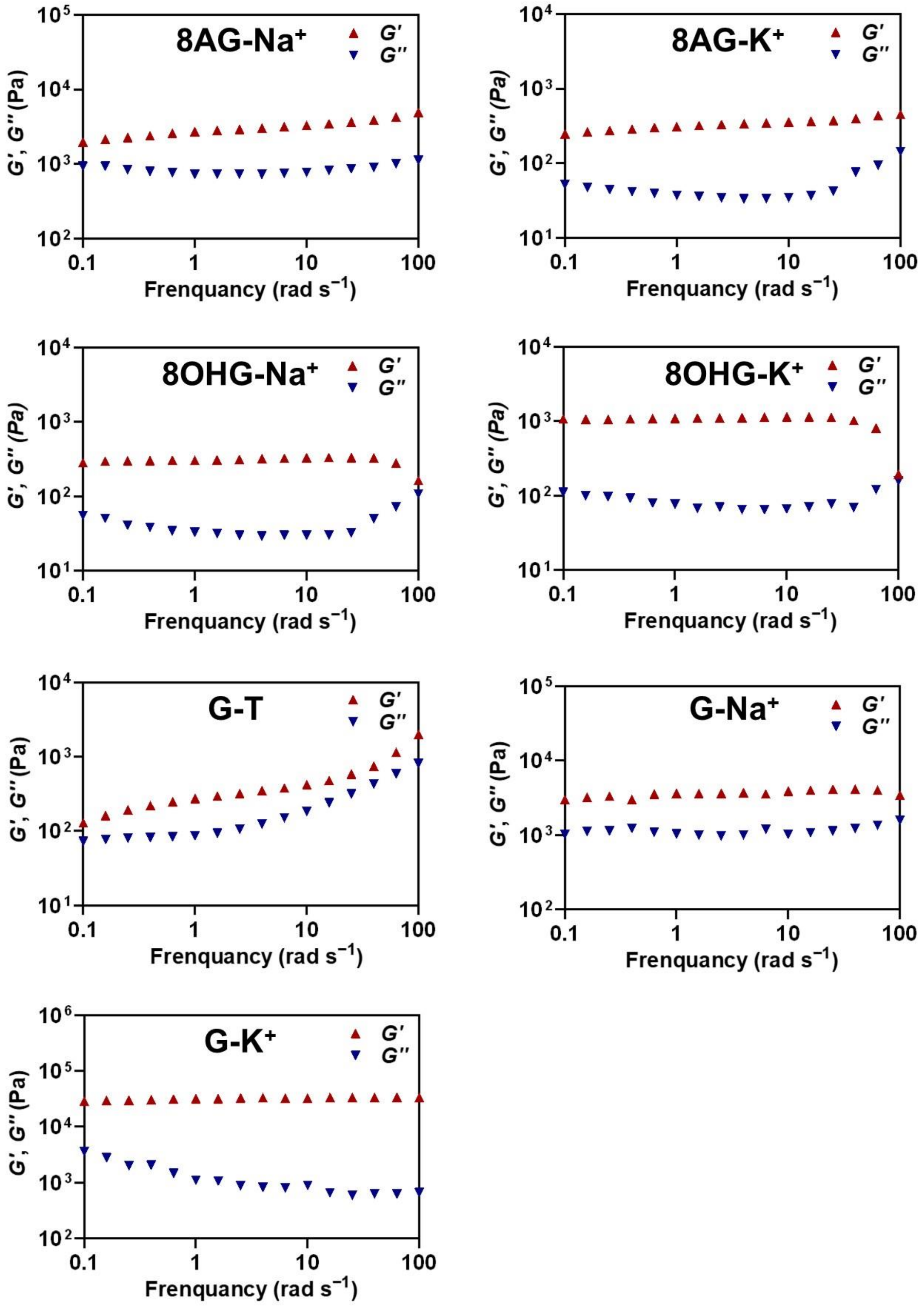

Supplementary Fig. 21. Evolution of *G*' and *G*'' as a function of frequency sweep by rheological measurements.

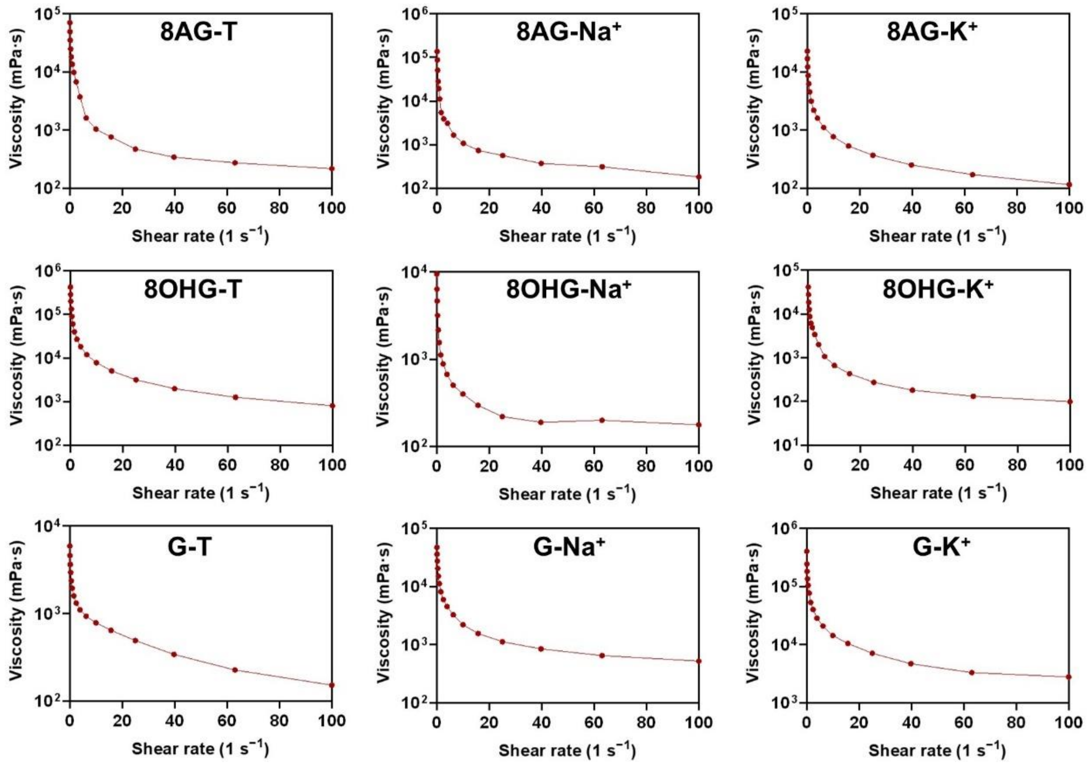

Supplementary Fig. 22. Viscosity test by rheological measurements. The hydrogels exhibit excellent shear-thinning properties because their viscosities decreased as the shear rate increased.

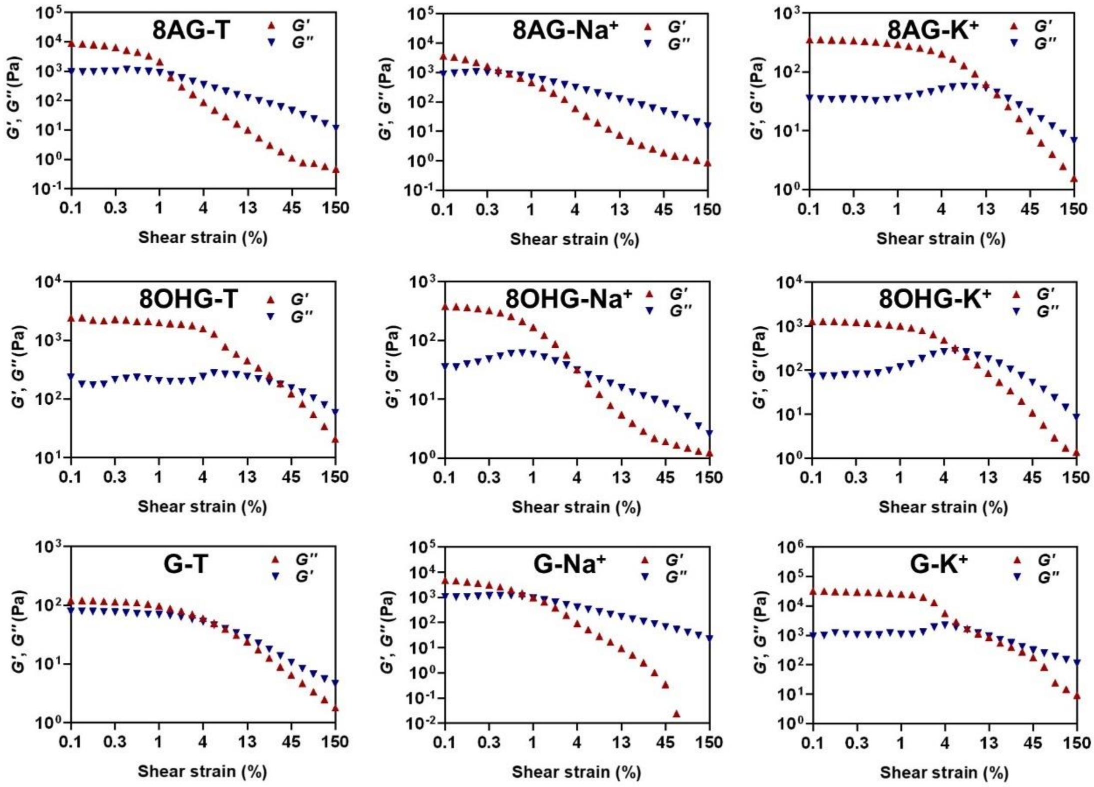

Supplementary Fig. 23. Evolution of *G*' and *G*" as a function of strain by rheological measurements.

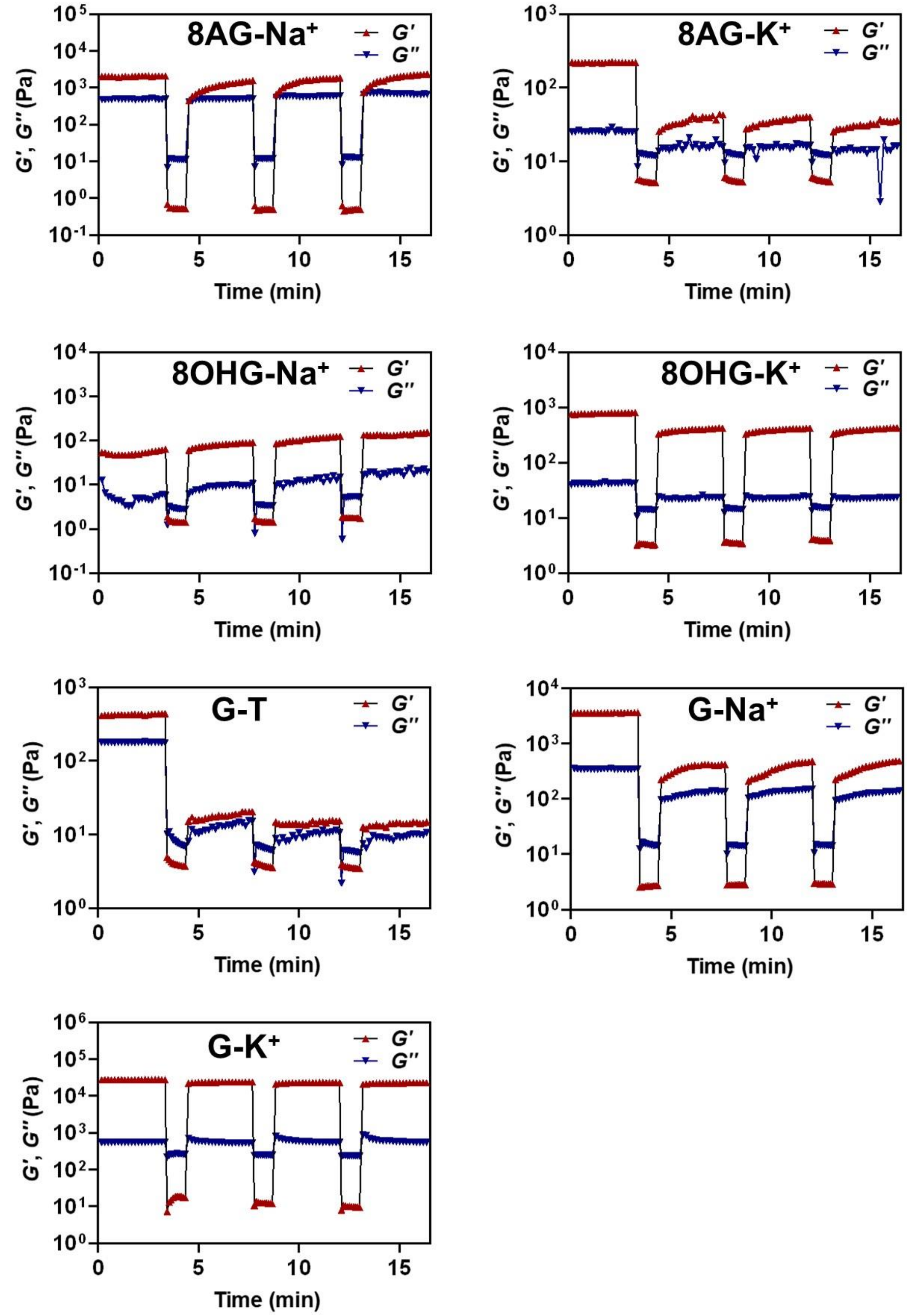

Supplementary Fig. 24. Evolution of *G*' and *G*'' as a function of self-healing by rheological measurements.

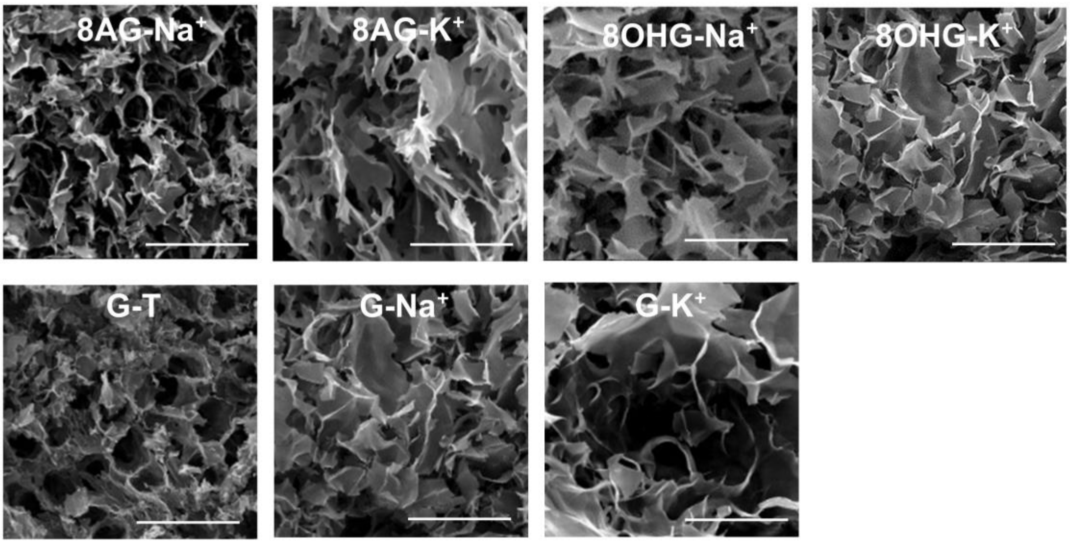

Supplementary Fig. 25. Scanning electron microscopy (SEM) image (scale bar: 50 μm) of 8AG-Na^+, 8AG-K^+, 8OHG-Na^+, 8OHG-K^+, G-T, G-Na^+, and G-K^+ hydrogels.

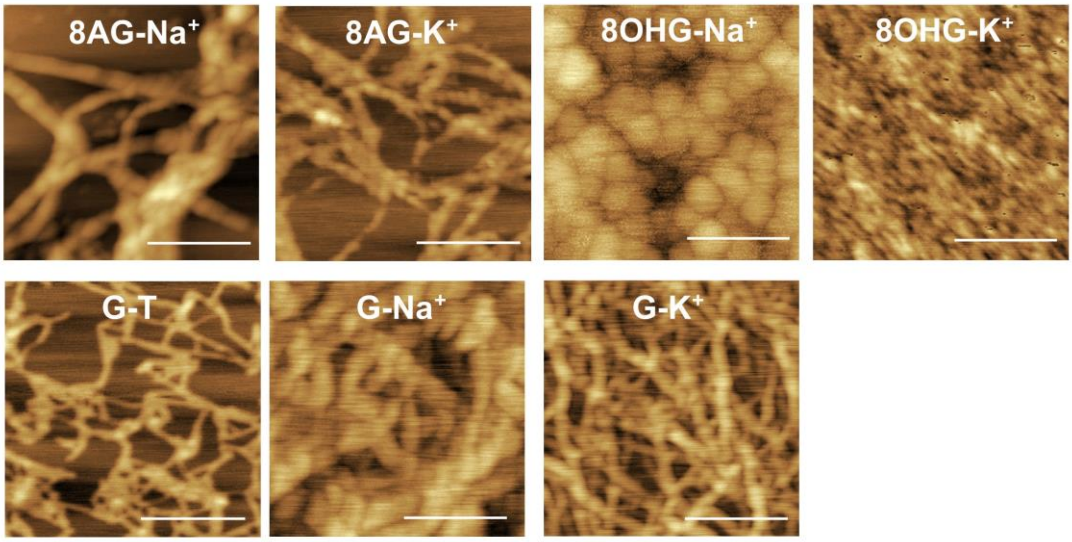

Supplementary Fig. 26. Atomic force microscopy (AFM) image (scale bar: 200 nm) of 8AG-Na^+, 8AG-K^+, 8OHG-Na^+, 8OHG-K^+, G-T, G-Na^+, and G-K^+ hydrogels.

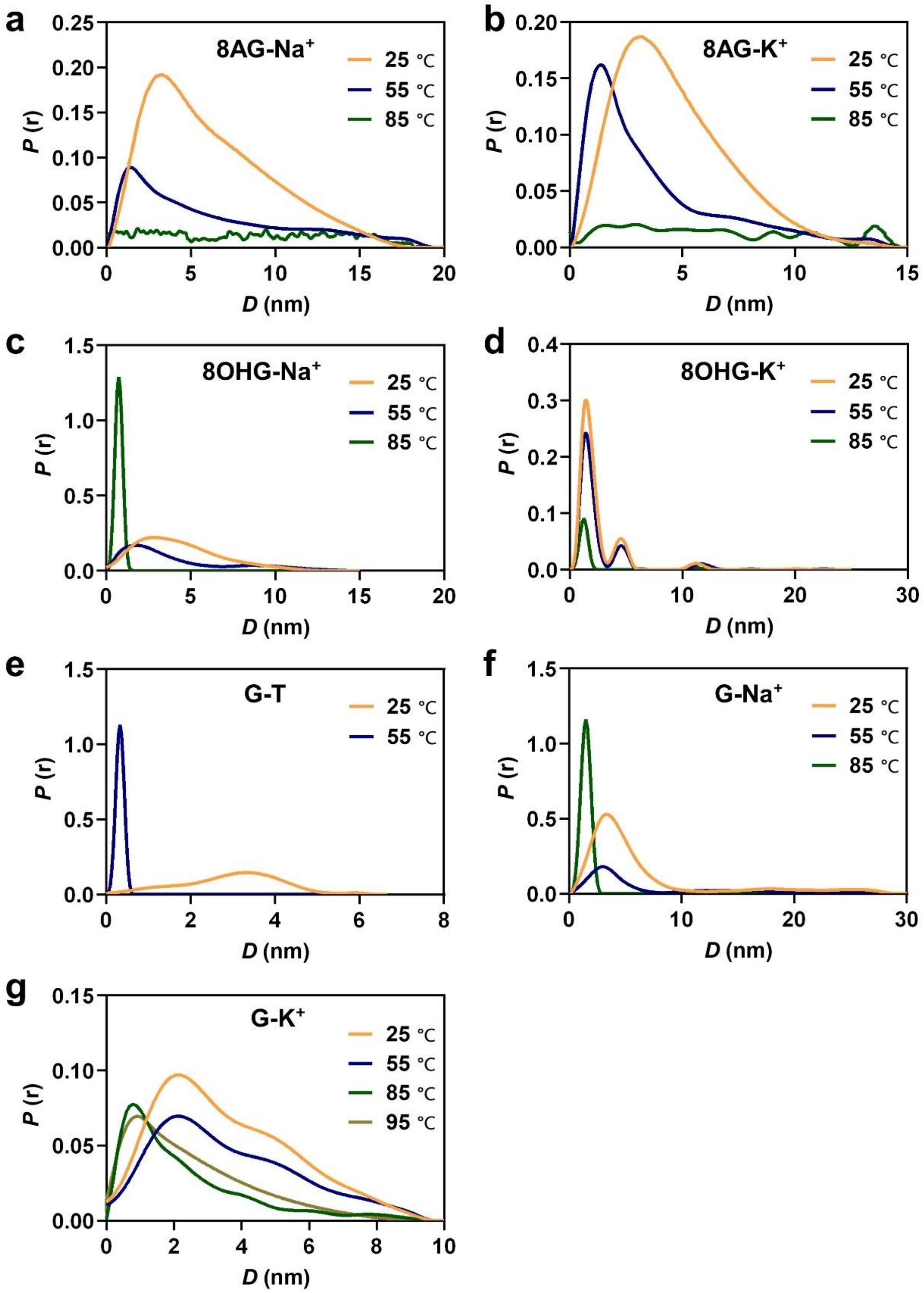

Supplementary Fig. 27. The PDDF profiles from variable-temperature small-angle X-ray scattering (VT-SAXS) experiments: 8AG-Na^+ (a), 8AG-K^+ (b), 8OHG-Na^+ (c), 8OHG-K^+ (d), G-T (e), G-Na^+ (f), and G-K^+ (g) hydrogels.

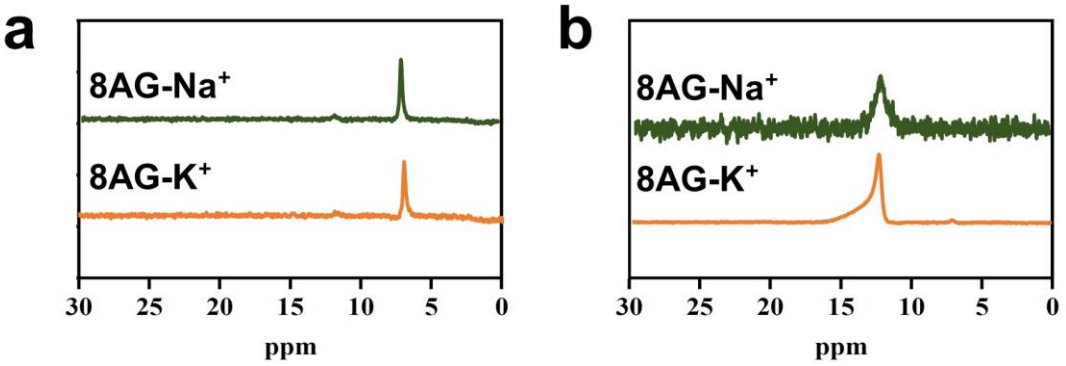

Supplementary Fig. 28. ^{11}B **nuclear magnetic resonance (NMR) spectra: 8AG-Na^{+} and 8AG-K^{+} hydrogels in D_2O (a) and 8OHG-Na^{+} and 8OHG-K^{+} hydrogels in DMSO-D_6 (b).**

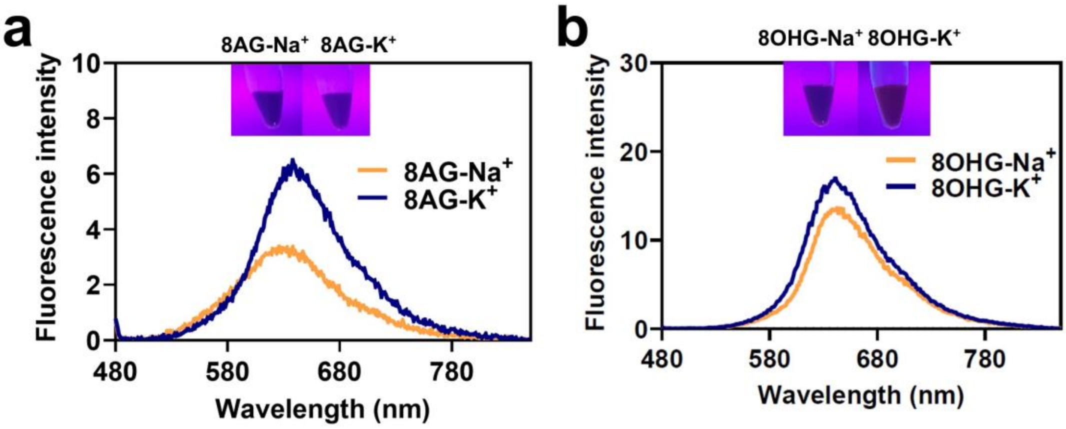

Supplementary Fig. 29. Fluorescence intensity of Alizarin Red S (ARS): 8AG-Na^{+} and 8AG-K^{+} hydrogels (a) and 8OHG-Na^{+} and 8OHG-K^{+} hydrogels (b).

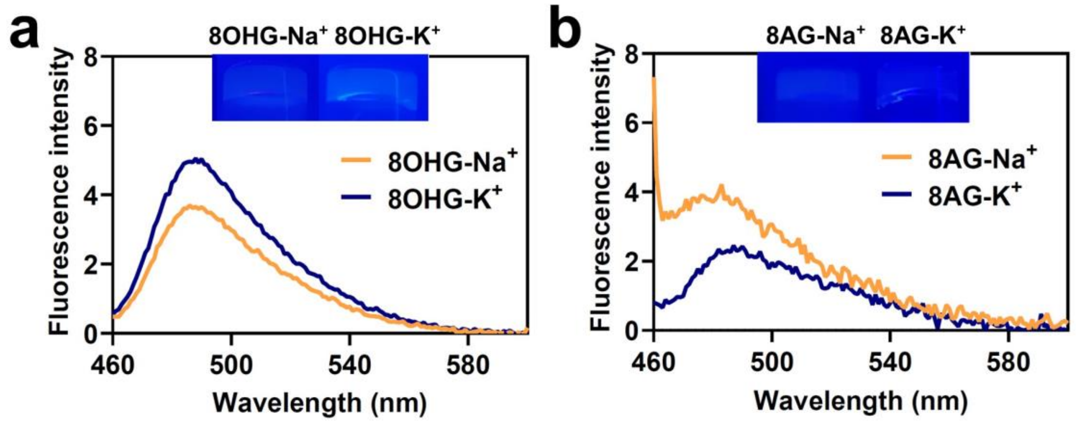

Supplementary Fig. 30. Thioflavin T (ThT) assay: 8AG-Na^{+} and 8AG-K^{+} hydrogels (a), 8OHG-Na^{+} and 8OHG-K^{+} hydrogels (b).

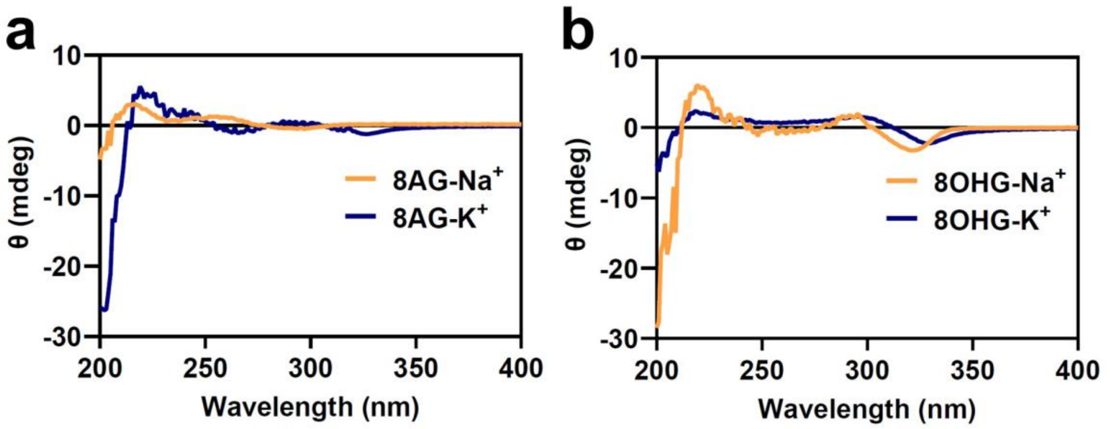

Supplementary Fig. 31. Circular dichroism spectra: 8AG-Na$^+$ and 8AG-K$^+$ hydrogels (a), 8OHG-Na$^+$ and 8OHG-K$^+$ hydrogels (b).

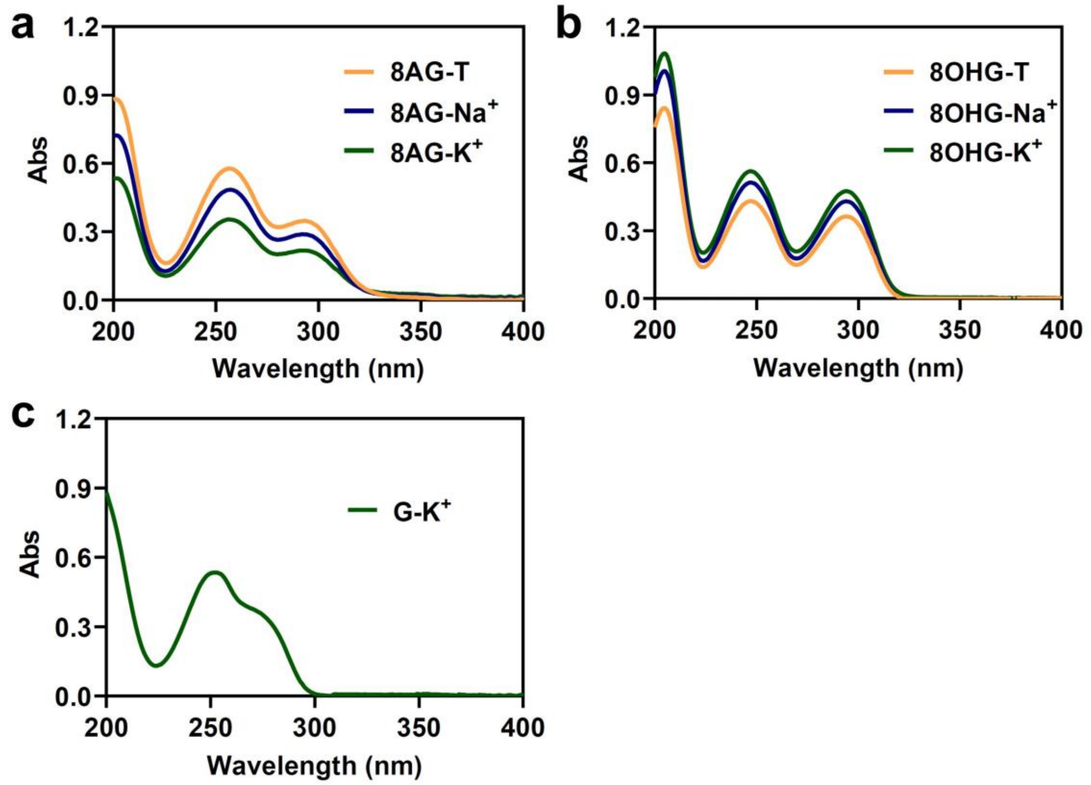

Supplementary Fig. 32. Ultraviolet (UV) spectra of hydrogels: 8AG-T, 8AG-Na$^+$ and 8AG-K$^+$ hydrogels (a); 8OHG-T, 8OHG-Na$^+$ and 8OHG-K$^+$ hydrogels (b); G-K$^+$ hydrogel (c).

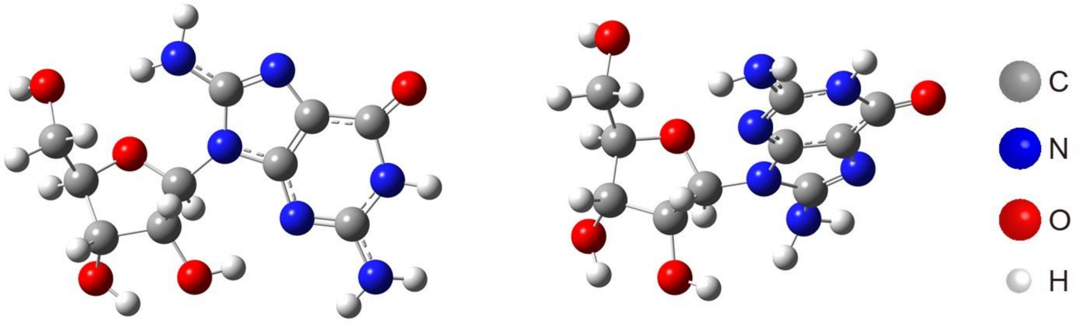

Anti-**6**
-1.8 kcal mol^{-1}

Syn-**6**
0.0 kcal mol^{-1}

Supplementary Fig. 33. The theoretical calculation of free energy difference of 6 with *anti/syn* conformation.

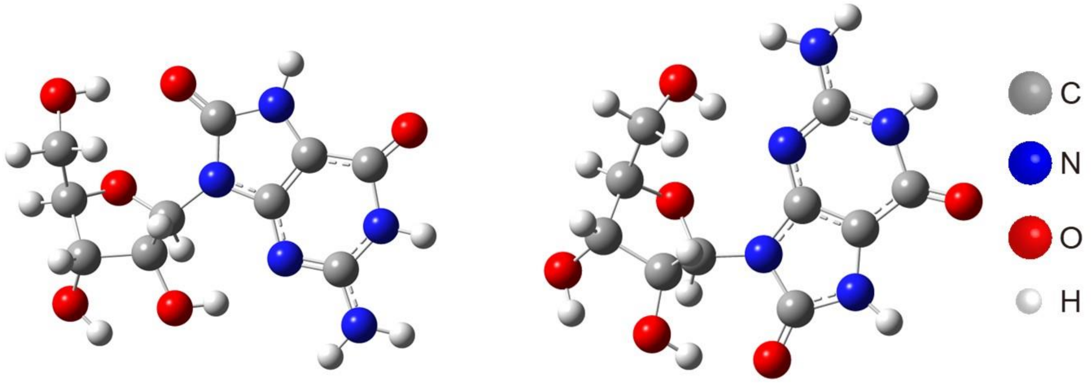

Anti-**8**
-2.7 kcal mol^{-1}

Syn-**8**
0.0 kcal mol^{-1}

Supplementary Fig. 34. The theoretical calculation of free energy difference of 8 with *anti/syn* conformation.

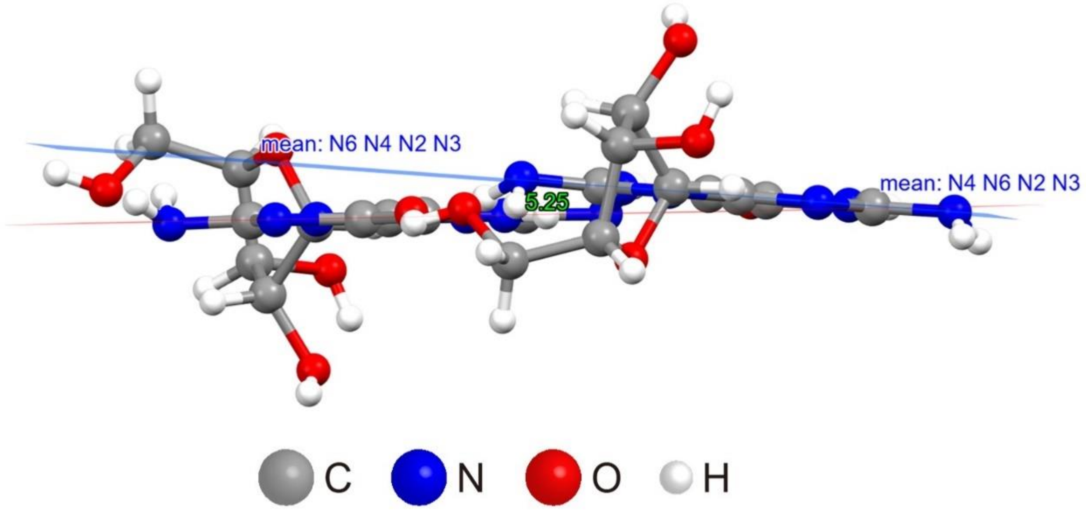

Supplementary Fig. 35. The bending angle of the base pairs in 6.

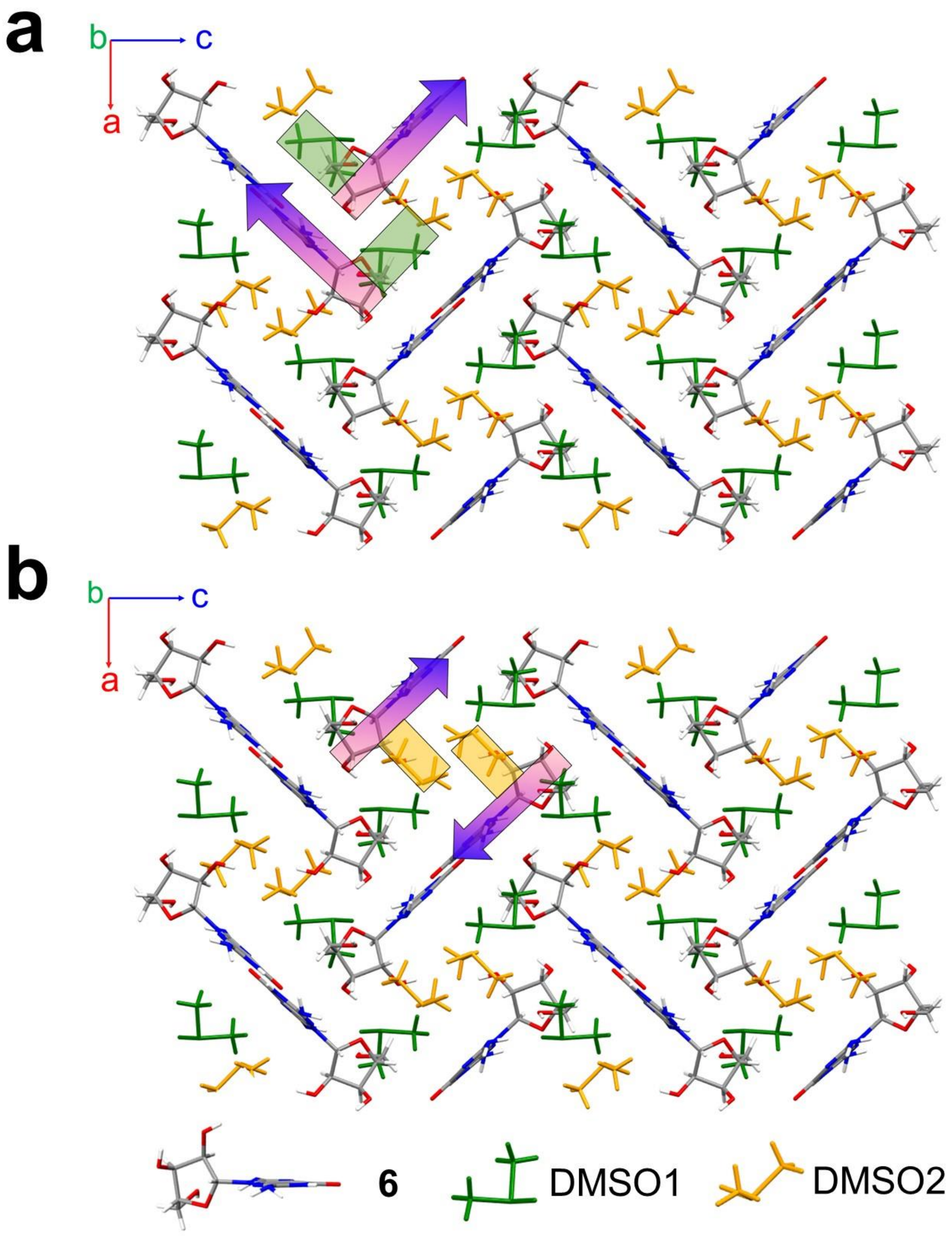

Supplementary Fig. 36. The single crystal structure of the interactions between DMSO1 and **6** (**a**) and between DMSO2 and **6** (**b**).

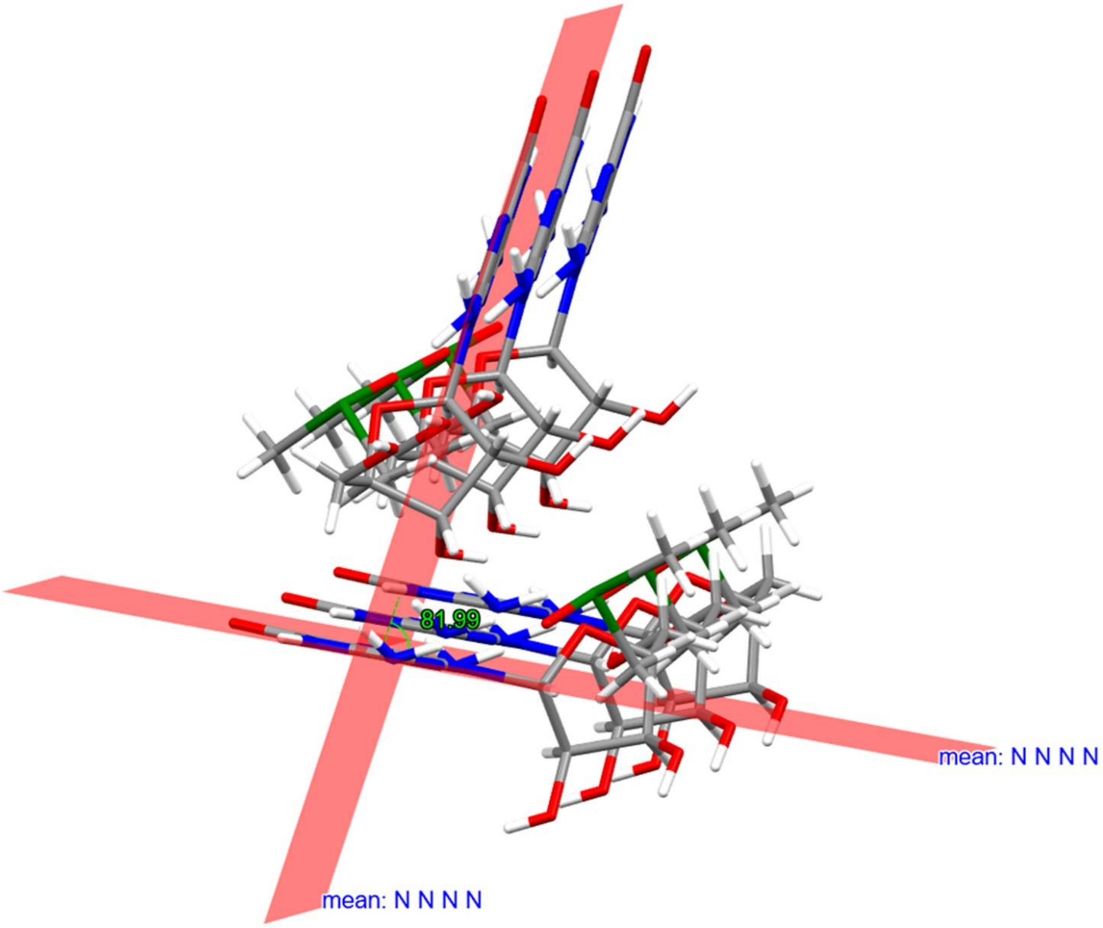

Supplementary Fig. 37. The bending angle of the bases connected by DMSO1.

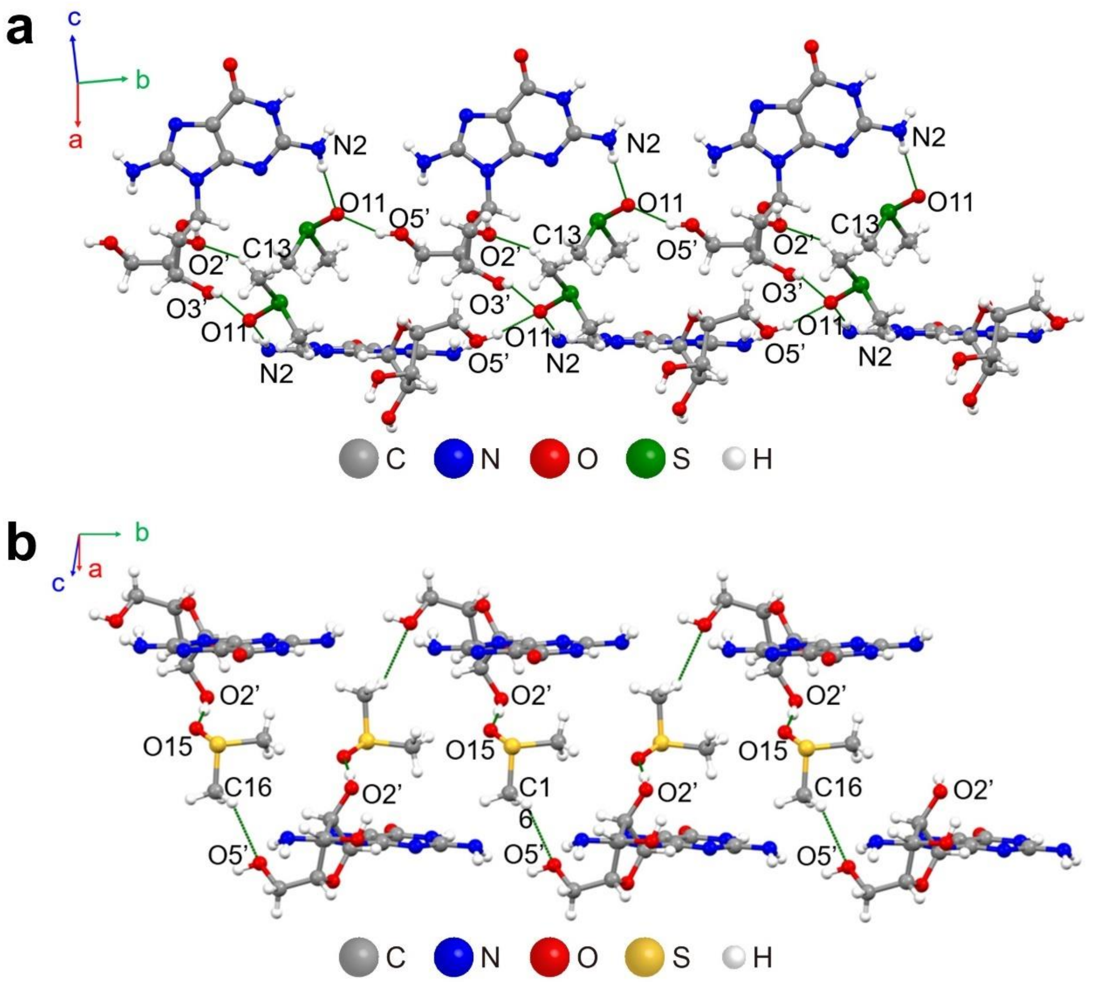

Supplementary Fig. 38. The single crystal structure of the interactions between DMSO1 and **6** (**a**), and between DMSO2 and **6** (**b**).

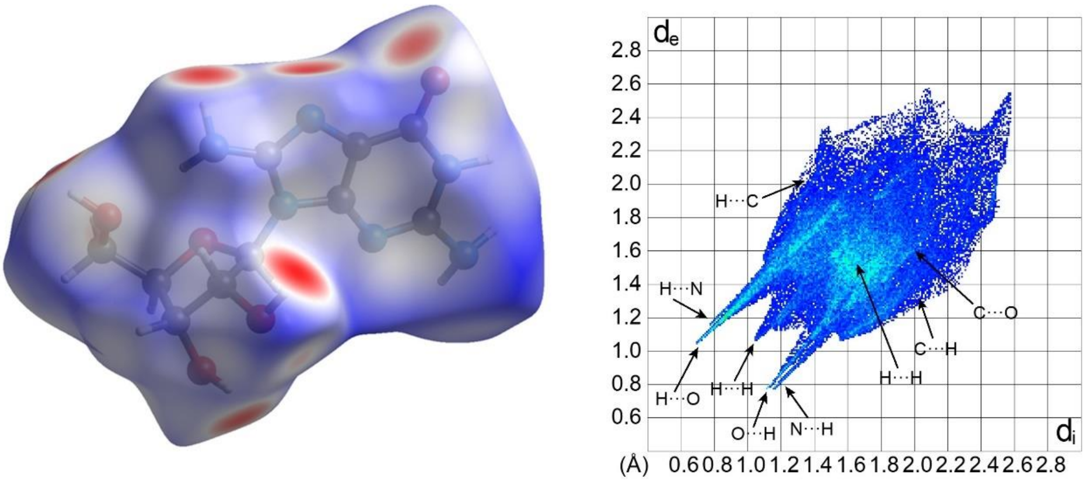

Supplementary Fig. 39. The 2D fingerprint plots and Hirshfeld surfaces (d_{norm}) of 6.

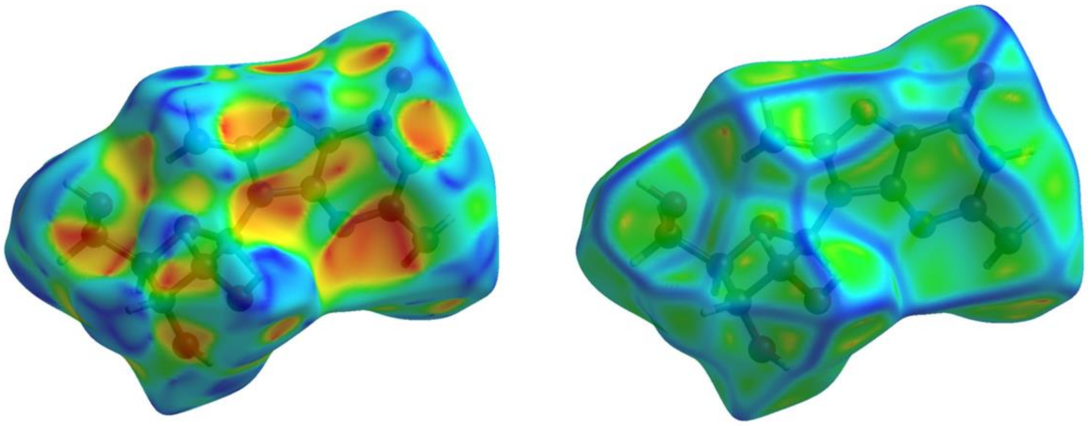

Supplementary Fig. 40. The Hirshfeld surfaces mapped over by shape index and curvedness images of 6.

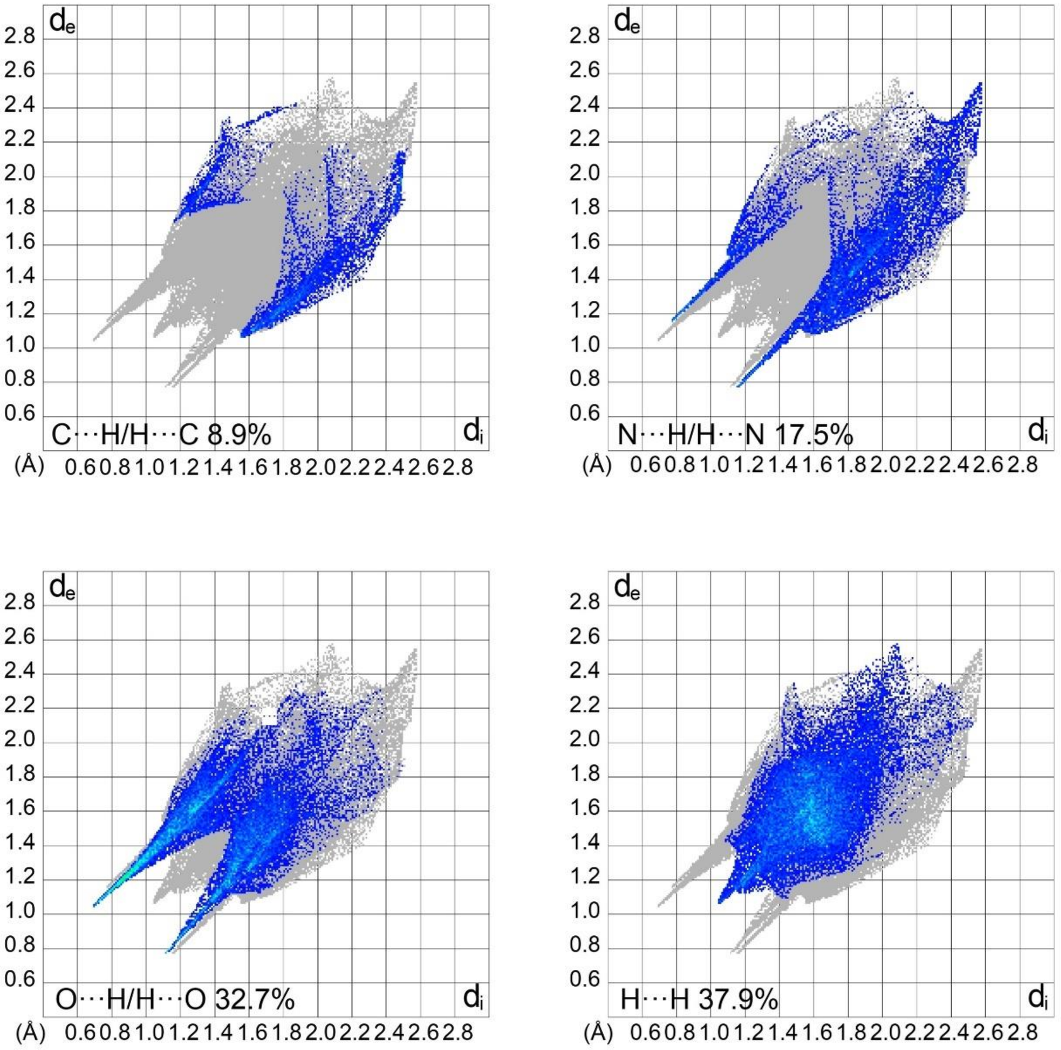

Supplementary Fig. 41. The classified decomposed close contacts 2D graphs of 6.

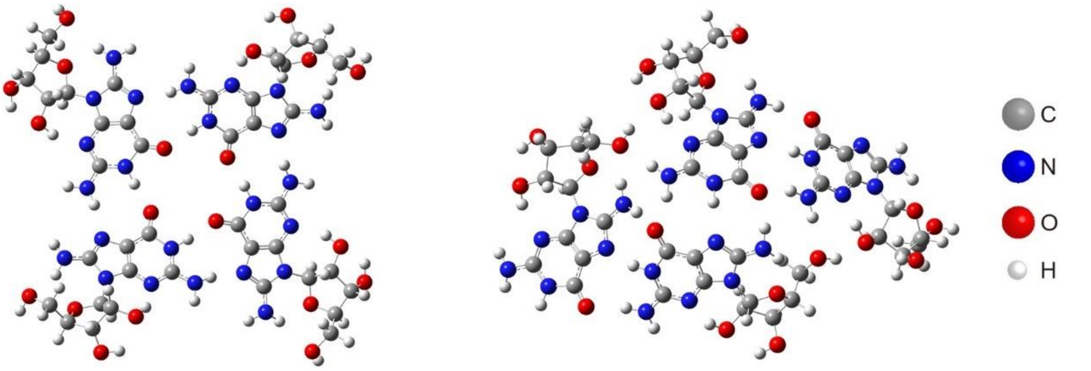

G-quartet of **6**
6.2 kcal mol^{-1}

G-ribbon of **6**
0.0 kcal mol^{-1}

Supplementary Fig. 42. The theoretical calculation of free energy difference of G-quartet and G-ribbon self-assembled by 6.

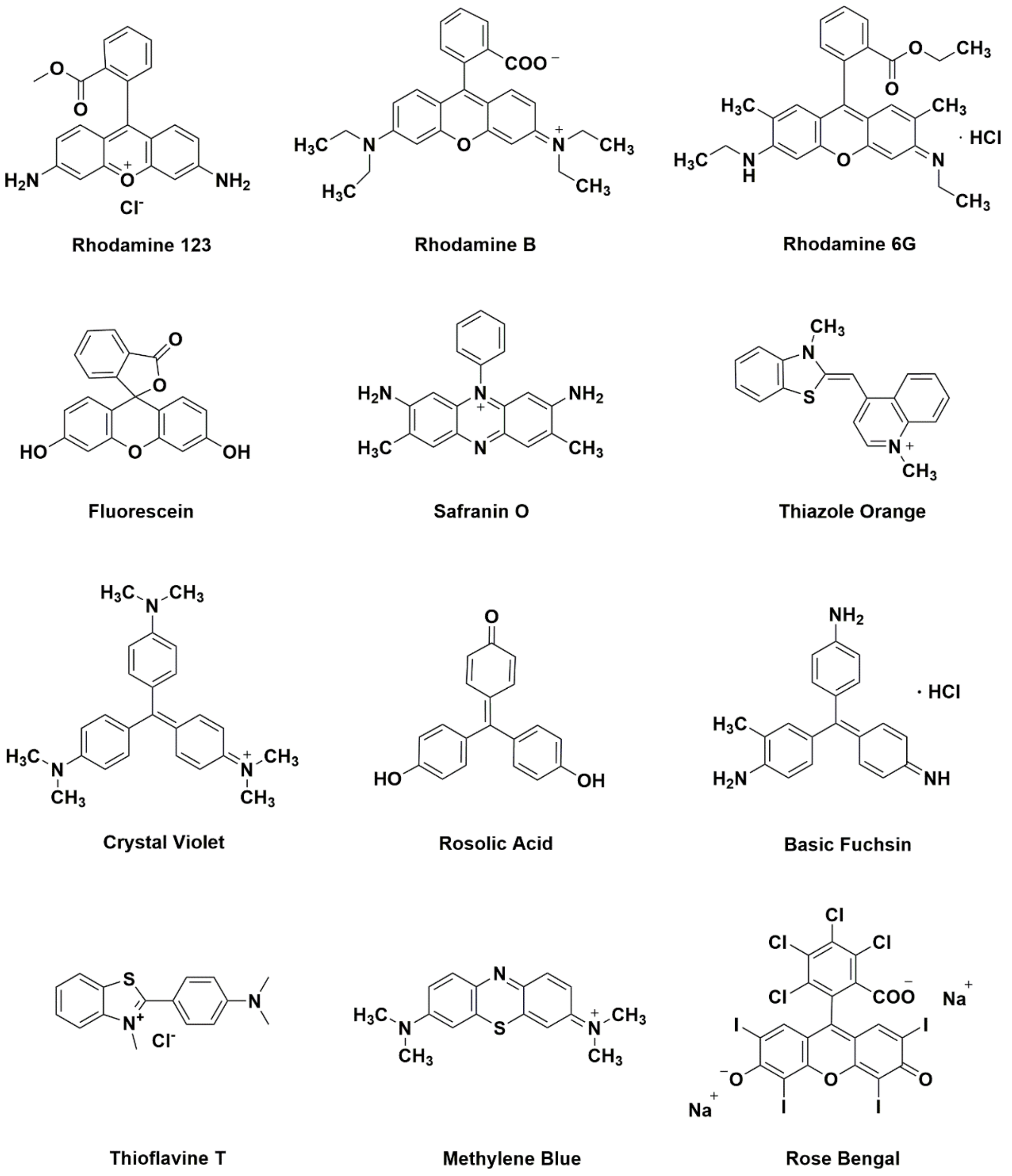

Supplementary Fig. 43. The chemical structures of the dyes including rhodamine 123 (Rho123), rhodamine B, rhodamine 6G, fluorescein, safranin O, fluorescein, thiazole orange, crystal violet, rosolic acid, basic fuchsin, thioflavin T, methylene blue, and rose bengal.

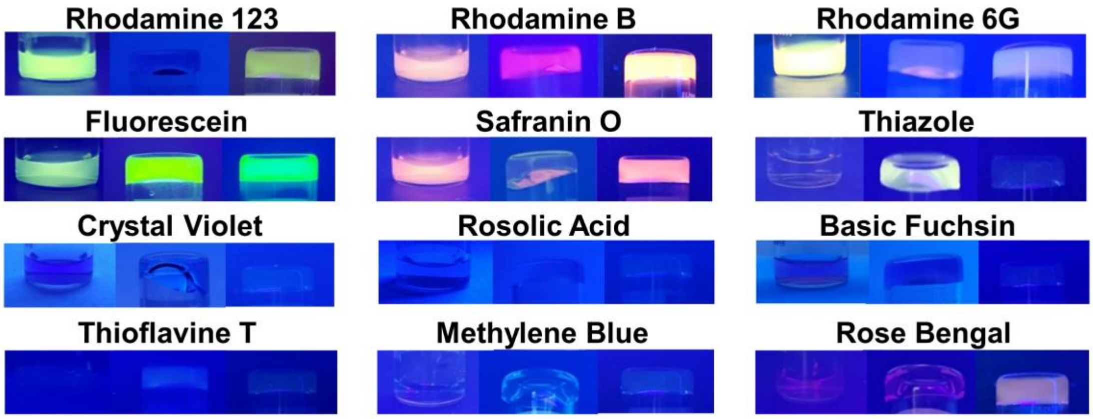

Supplementary Fig. 44. The fluorescence of 8AG-T and 8OHG-T hydrogels mixed with dyes. From left to right are the dye solution, 8OHG-T hydrogel mixed with dyes, and 8AG-T hydrogel mixed with dyes.

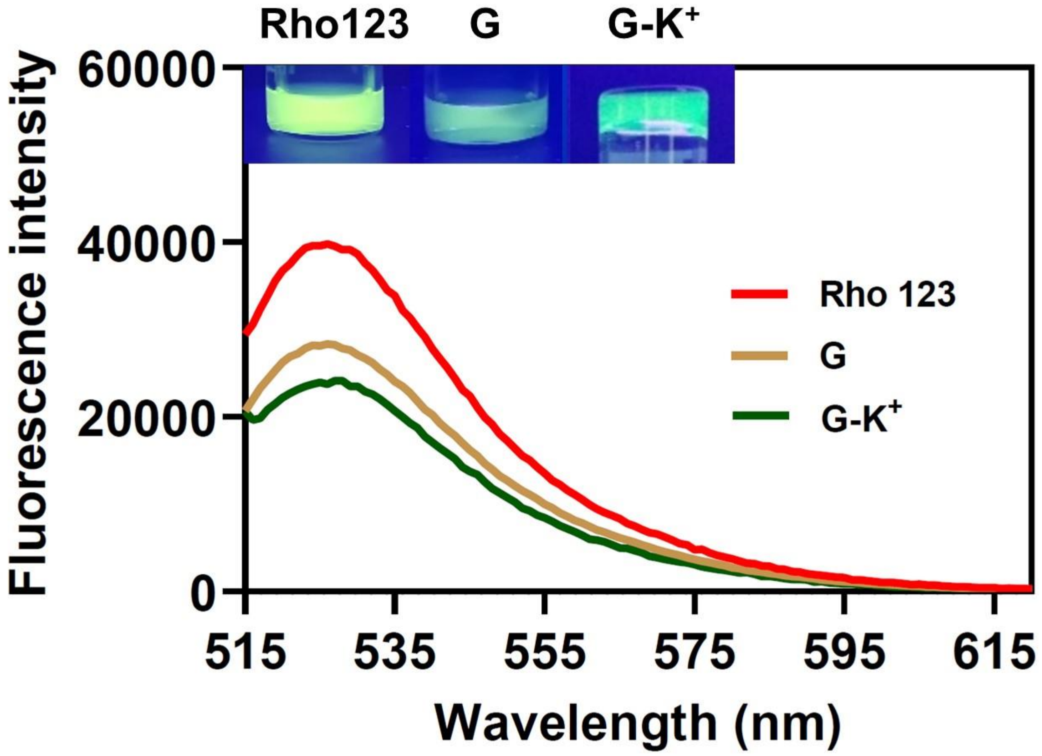

Supplementary Fig. 45. The fluorescence of G-K^+ hydrogels after adding Rho123.

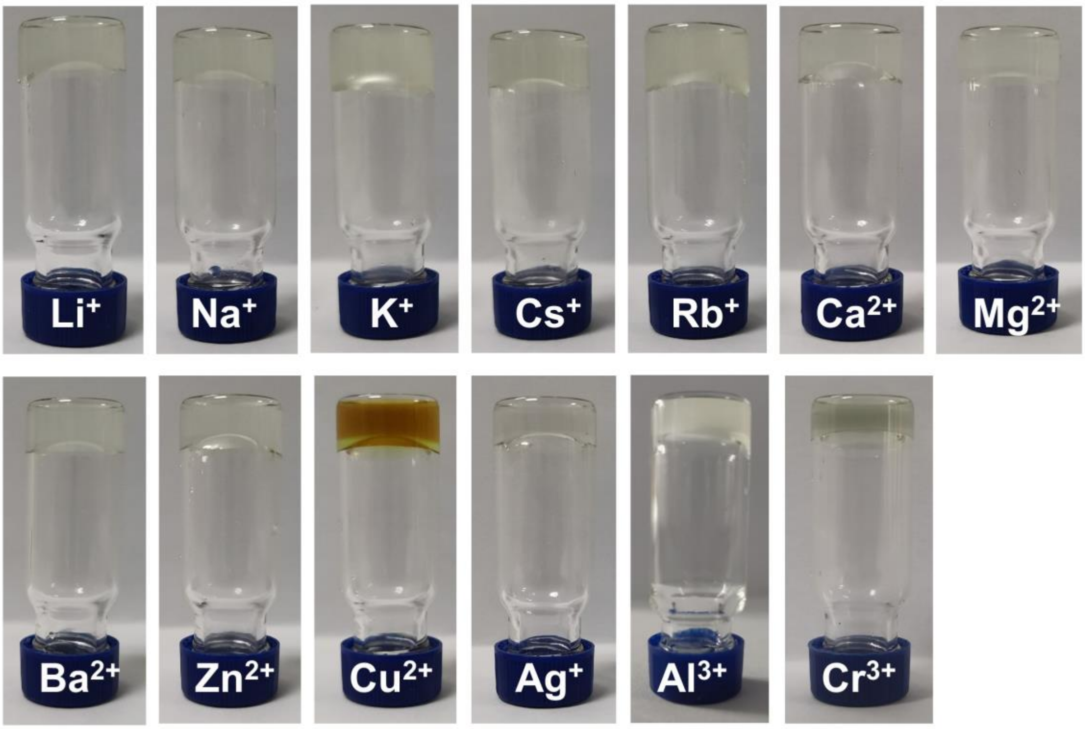

Supplementary Fig. 46. The photographs of 8AG-T hydrogels after adding ionic solutions.

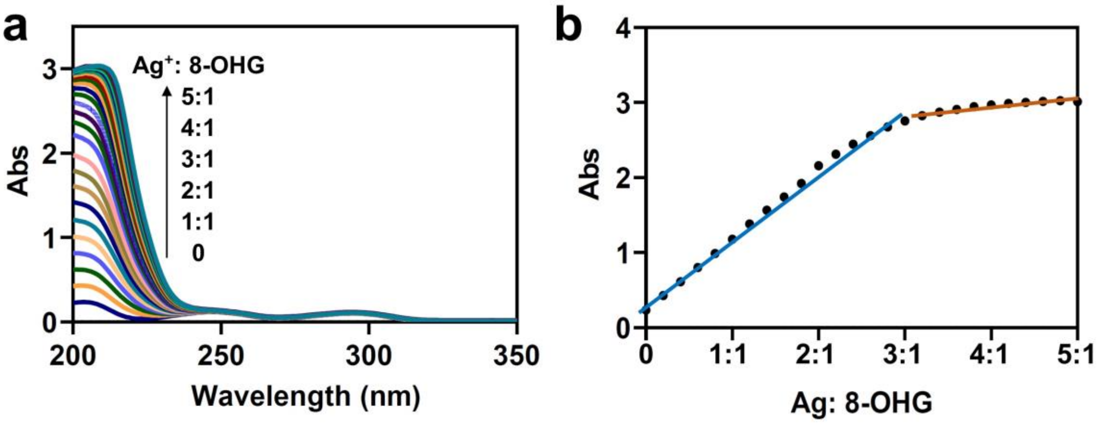

Supplementary Fig. 47. Ultraviolet (UV) spectrophotometric titration of 8OHG-T hydrogel with increasing Ag^+. a UV spectra of 8OHG-T hydrogel. **b** Absorbances of 8OHG-T hydrogel at 204 nm.

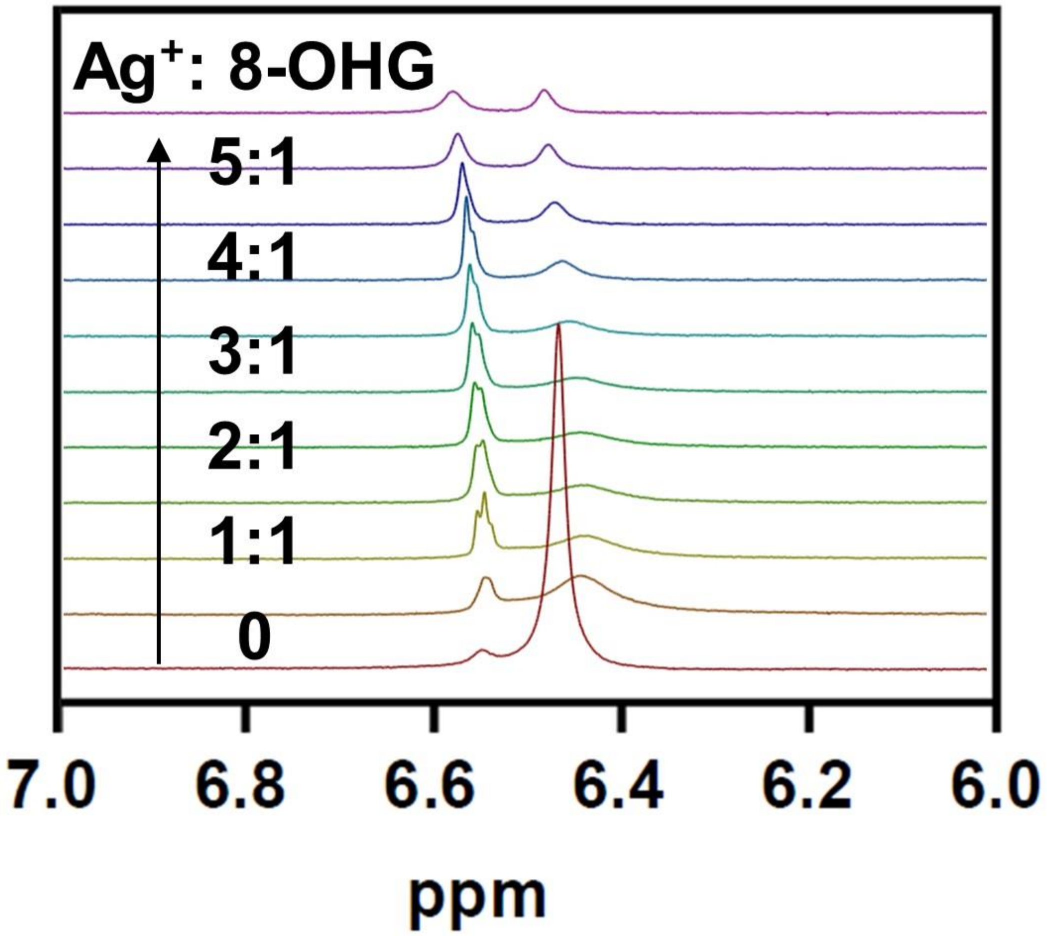

Supplementary Fig. 48. ^1H nuclear magnetic resonance (NMR) spectra of 8OHG-T hydrogel with the titration of Ag^+. The peaks represent C2-NH_2 in **8**, indicating the Ag^+ binding site of 8OHG-T hydrogel is C2N group.

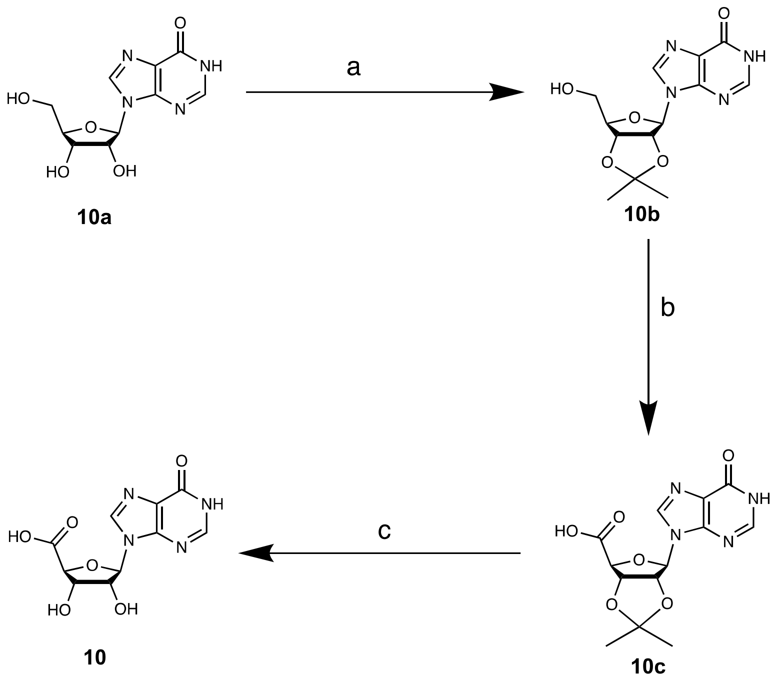

Supplementary Fig. 49. Synthesis of inosine-5'-carboxylic acid (10).

Reagents and conditions: a) $HClO_4$, acetone, 24 h; b) $NaHCO_3$, TEMPO, iodobenzene diacetate, 6.5 h; c) 1 N HCl, 2 h.

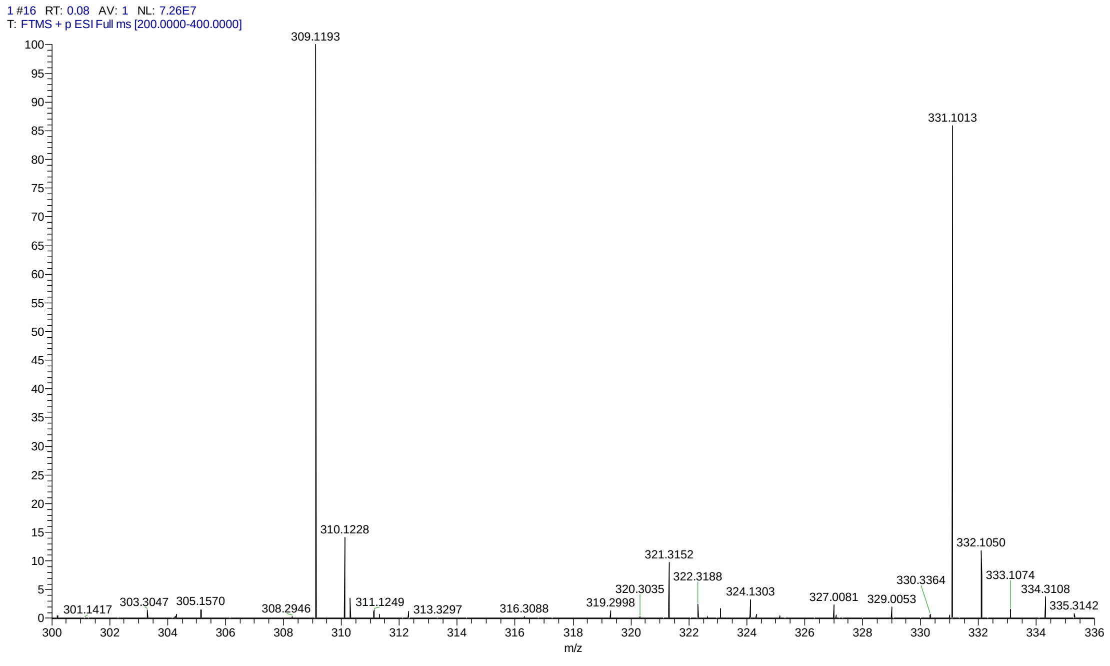

Supplementary Fig. 50. HRMS data of 2',3'-*O*-isopropylideneinosine (10b).

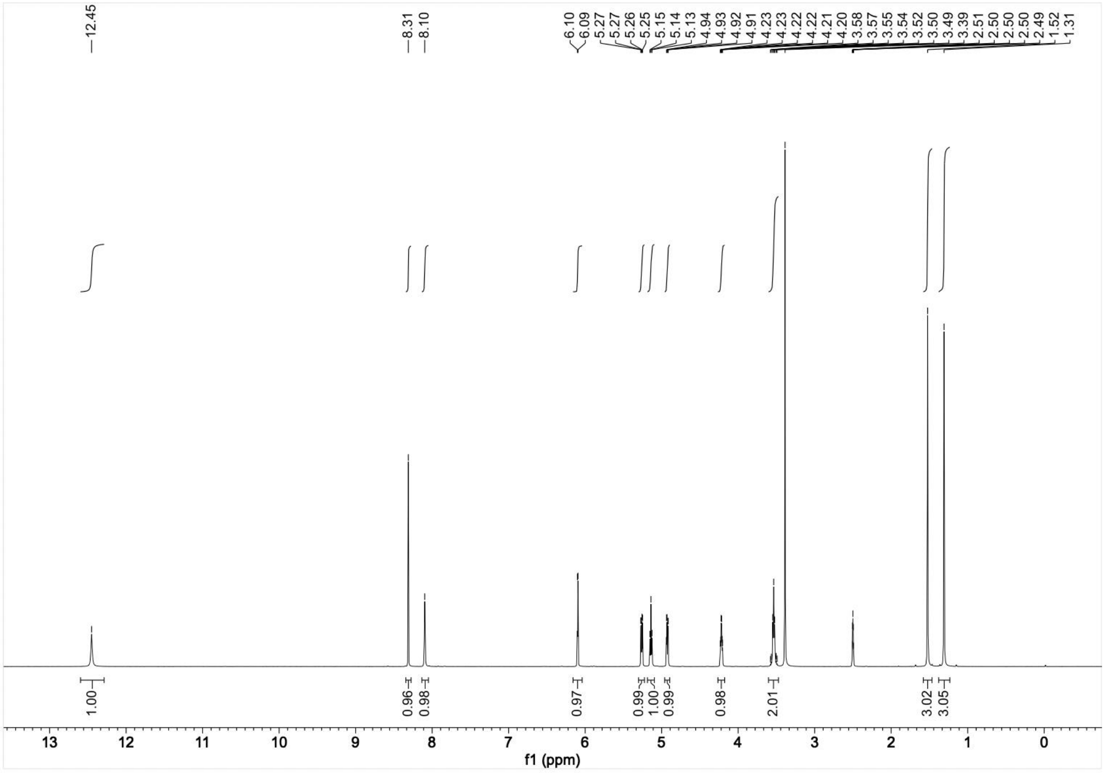

Supplementary Fig. 51. ^{1}H NMR spectrum of 2’,3’-*O*-isopropylideneinosine (10b).

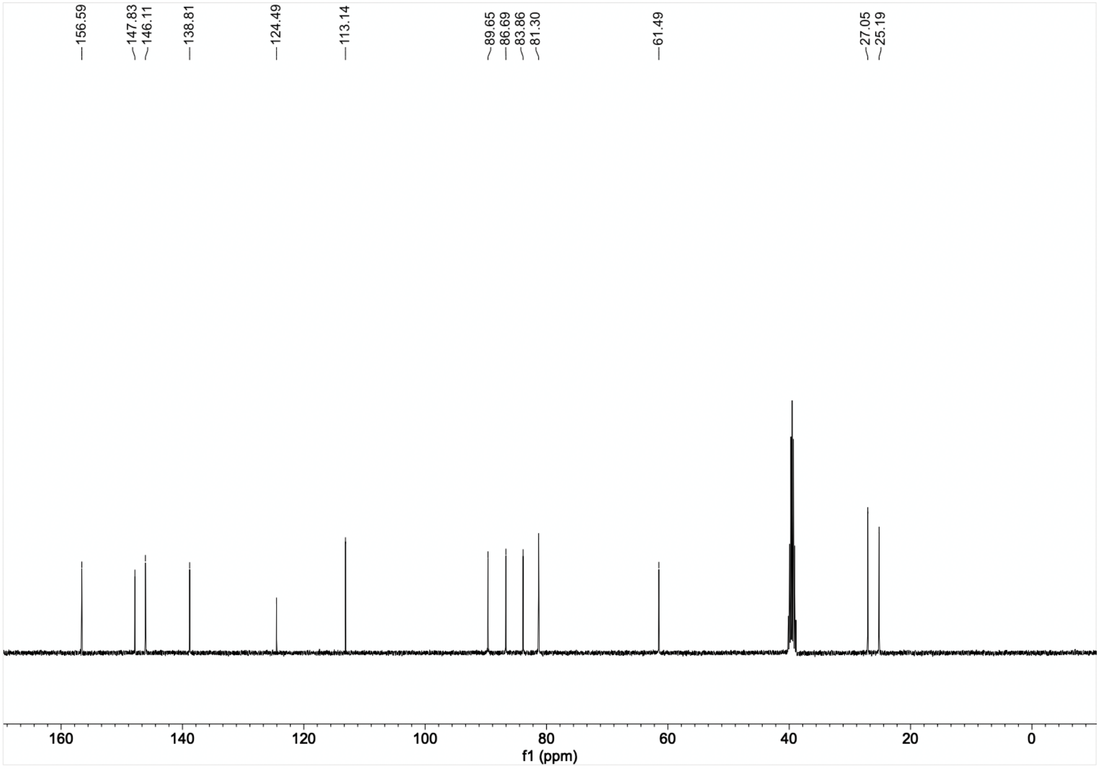

Supplementary Fig. 52. ^{13}C NMR spectrum of 2’,3’-*O*-isopropylideneinosine (10b).

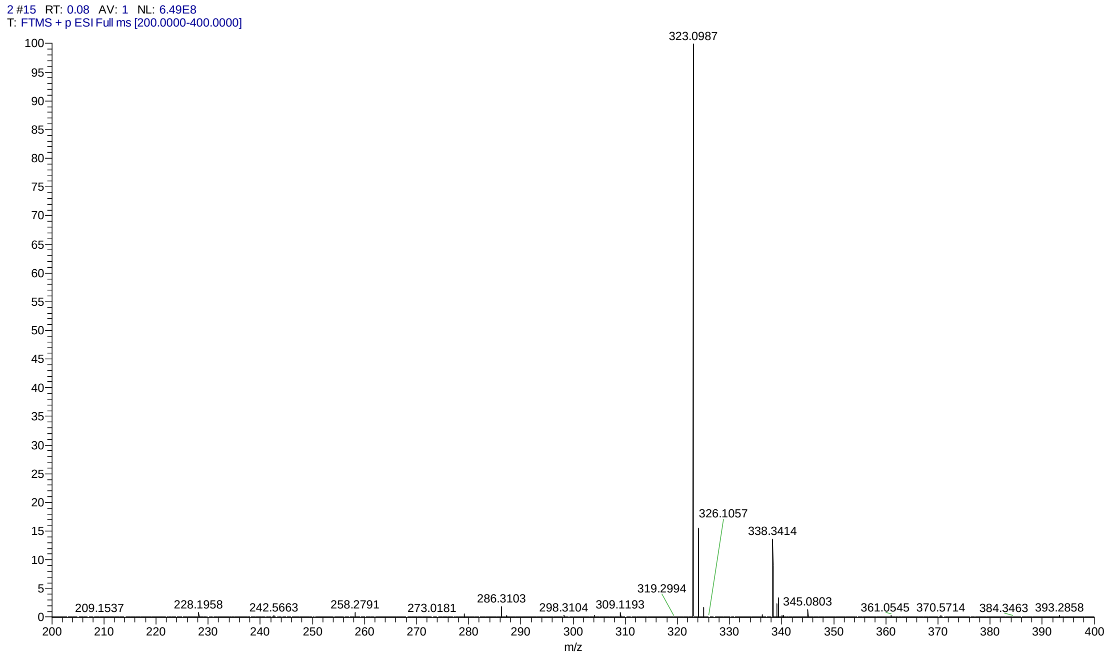

Supplementary Fig. 53. HRMS data of 2’,3’-*O*-isopropylideneinosine 5’-carboxylic acid (10c).

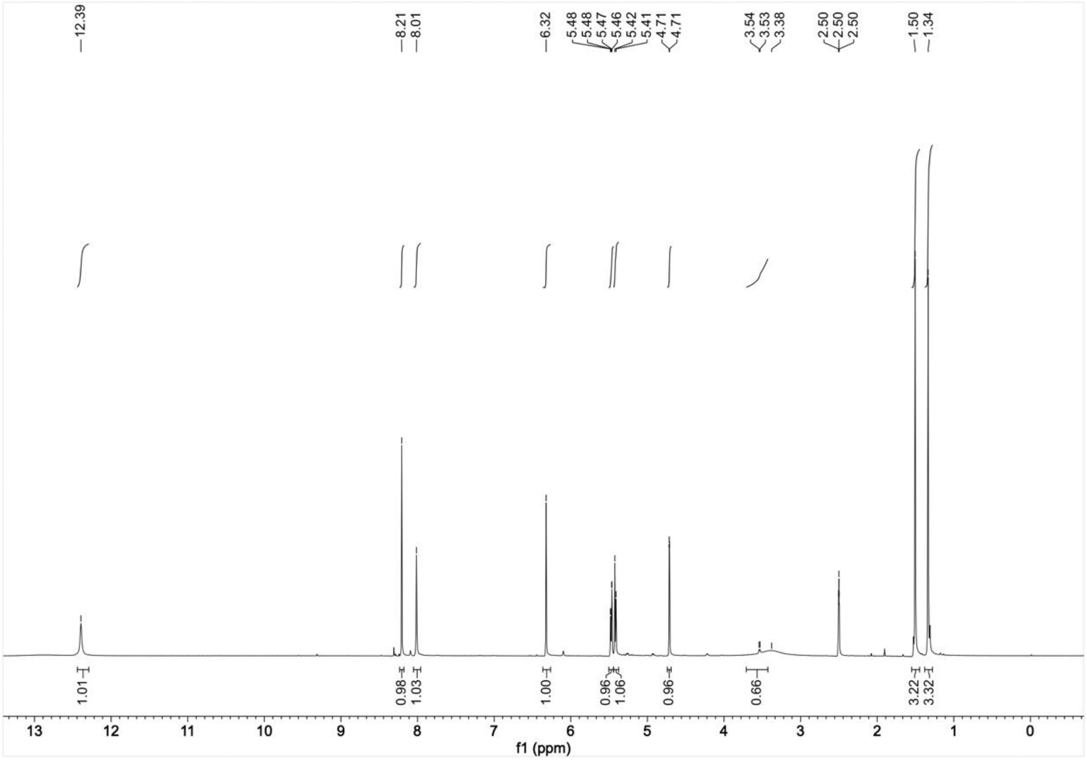

Supplementary Fig. 54. ^{1}H NMR spectrum of 2',3'-*O*-isopropylideneinosine 5’-carboxylic acid (10c).

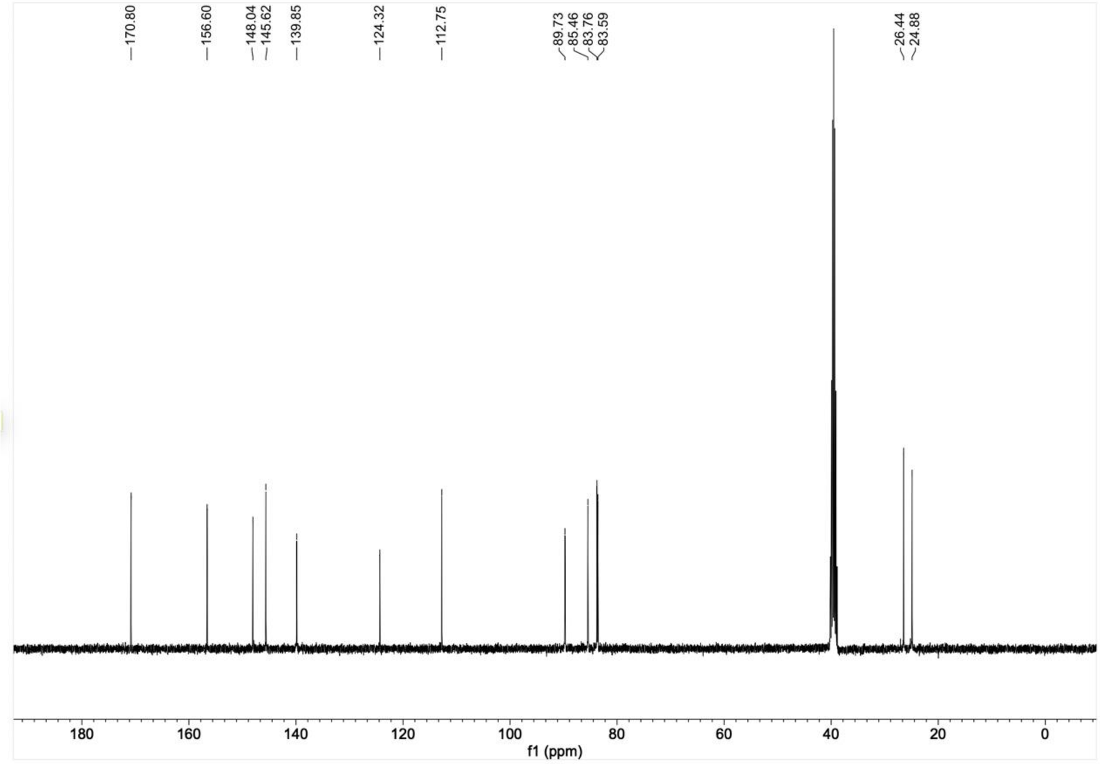

Supplementary Fig. 55. ^{13}C NMR spectrum of 2',3'-*O*-isopropylideneinosine 5'-carboxylic acid (10c).

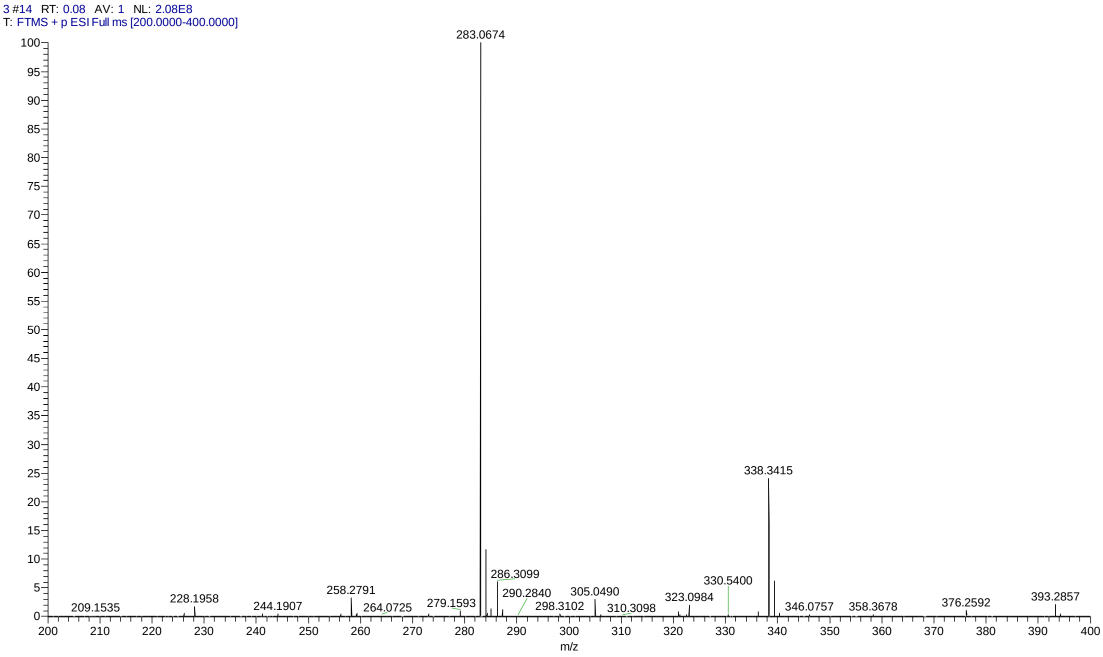

Supplementary Fig. 56. HRMS data of inosine-5'- carboxylic acid (10).

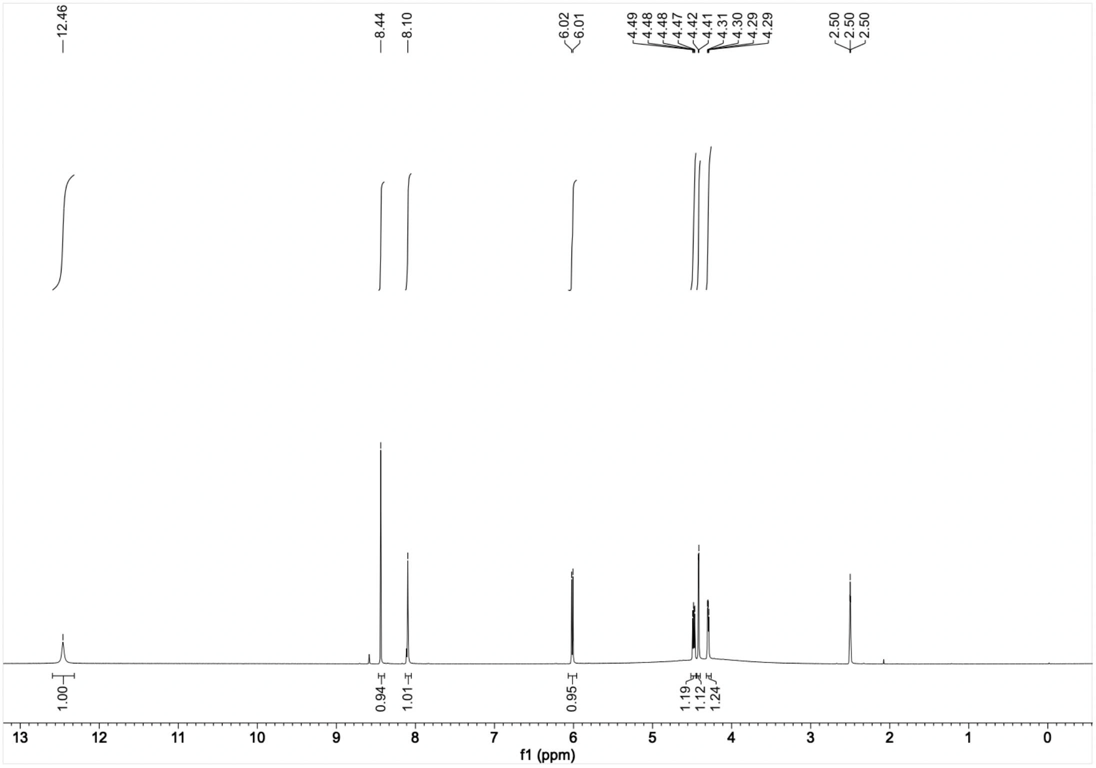

Supplementary Fig. 57. ^{1}H NMR spectrum of inosine-5'-carboxylic acid (10).

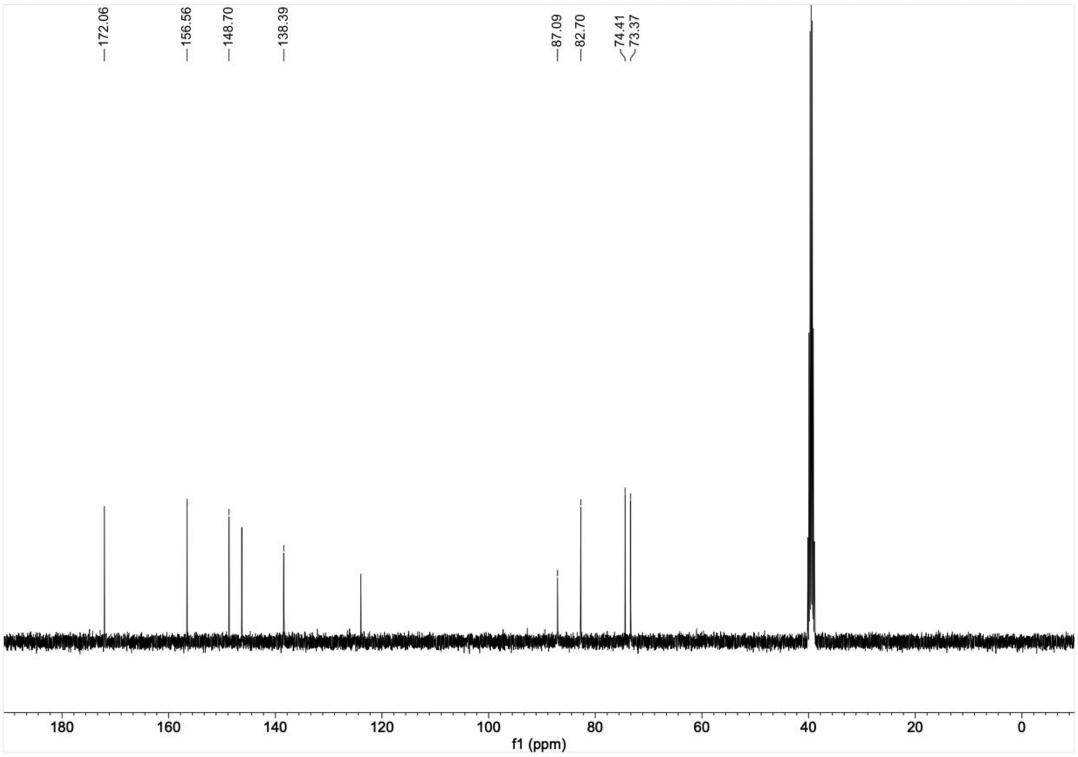

Supplementary Fig. 58. ^{13}C NMR spectrum of inosine-5'-carboxylic acid (10).

4. Supplementary Tables

Supplementary Table 1. Characteristics of the prediction models

Features	Algorithms	Rows	Columns
Molecular Descriptors			
Descriptors-4175	LR, DT, RF, XGBoost#	71*	4175
Descriptors-144	LR, DT, RF, XGBoost	71	144
Descriptors-40	LR, DT, RF, XGBoost	71	40
Descriptors-REF&	LR, DT, RF, XGBoost	71	16-37&

Notes: * Rows: Whether the nucleoside derivatives have the hydrogel-forming ability.

#Algorithms: Logistic regression (LR), decision tree (DT), random forest (RF), and extreme gradient boosting (XGBoost);

&Descriptors-REF: Recursive feature elimination (REF) has different optimal descriptors for different Algorithms: LR, n=24; XGBoost, n=16; DT, n= 30; RF, n=37.

Supplementary Table 2. The results of feature importance of 24 descriptors for logistic regression.

Descriptors Information		Feature importance		
Name	Description	PFI		Coefficient*
		Mean	SEM	
CATS2D_06_DL	Pharmacophore descriptors	0.018	0.005	-0.090
B09[O-O]	2D Atom Pairs	0.018	0.003	0.155
P_VSA_charge_7	P_VSA-like descriptors	0.014	0.003	-0.083
H-052	Atom-centred fragments	0.014	0.003	-0.133
CATS2D_03_DL	Pharmacophore descriptors	0.011	0.004	-0.072
nN(CO)2	Functional group counts	0.009	0.004	0.131
CATS2D_04_AA	Pharmacophore descriptors	0.006	0.005	0.053
CATS2D_05_DA	Pharmacophore descriptors	0.006	0.005	0.057
C-016	Atom-centred fragments	0.004	0.005	-0.099
F07[N-O]	2D Atom Pairs	0.001	0.003	0.085
VE1sign_Dz(v)	2D matrix-based descriptors	0.000	0.006	-0.054
CATS2D_05_DL	Pharmacophore descriptors	-0.001	0.003	-0.087
F05[N-N]	2D Atom Pairs	-0.001	0.003	0.092
P_VSA_charge_4	P_VSA-like descriptors	-0.001	0.003	-0.103
F10[O-O]	2D Atom Pairs	-0.002	0.005	-0.083
MATS3p	2D autocorrelations	-0.004	0.005	-0.055
VE3sign_D/Dt	2D matrix-based descriptors	-0.005	0.004	-0.082
SM10_AEA (dm)	Edge adjacency indices	-0.005	0.005	-0.076
SpDiam_AEA(ed)	Edge adjacency indices	-0.006	0.005	-0.068
VE1sign_B(p)	2D matrix-based descriptors	-0.008	0.003	0.057
GATS6i	2D autocorrelations	-0.008	0.003	0.059
SpMAD_EA(ri)	Edge adjacency indices	-0.012	0.003	0.067
GATS7s	2D autocorrelations	-0.014	0.007	0.077
CATS2D_09_DA	Pharmacophore descriptors	-0.016	0.008	0.076

Notes: *: Permutation feature importance (PFI), logistic regression (LR), decision tree (DT), random forest (RF), and extreme gradient boosting (XGBoost), standard error of the mean (SEM).

&

Supplementary Table 3. The result of AUC (Area under Curve) and validation accuracy for all models based on training set.

Models	Features	Training set performance			
		Validation accuracy		AUC	
		Mean	SEM	Mean	SEM
DT*	Descriptor_4175	0.68	0.02	0.68	0.02
LR	Descriptor_4175	0.58	0.02	0.58	0.03
RF	Descriptor_4175	0.64	0.02	0.72	0.02
XGBoost	Descriptor_4175	0.63	0.02	0.66	0.02
DT	Descriptor_119	0.62	0.02	0.62	0.01
LR	Descriptor_119	0.64	0.02	0.80	0.02
RF	Descriptor_119	0.67	0.02	0.74	0.02
XGBoost	Descriptor_119	0.64	0.02	0.68	0.02
DT	Descriptor_34	0.67	0.02	0.67	0.02
LR	Descriptor_34	0.70	0.01	0.84	0.02
RF	Descriptor_34	0.68	0.01	0.75	0.02
XGBoost	Descriptor_34	0.67	0.01	0.73	0.02
DT	Descriptor_ REF[#]	0.63	0.02	0.65	0.02
LR	Descriptor_ REF[#]	0.70	0.01	0.84	0.02
RF	Descriptor_ REF[#]	0.67	0.02	0.75	0.02
XGBoost	Descriptor_ REF[#]	0.67	0.02	0.74	0.02

Notes: *: Logistic regression (LR), decision tree (DT), random forest (RF), and extreme gradient boosting (XGBoost), standard error of the mean (SEM).
#: Descriptors-REF: Recursive feature elimination (REF) has different optimal descriptors for different Algorithms: LR, n=34; XGBoost, n=33; DT, n= 23; RF, n=26.

Supplementary Table 4. The *g*-factor (ratio between CD and absorption intensities) of hydrogels at the wavelength of 219 nm.

Hydrogels	Wavelength (nm)	θ (mdeg)	A	*g*-factor
8AG-T	219	2.41	0.23	0.31
8AG-Na^{+}	219	3.03	0.18	0.51
8AG-K^{+}	219	2.78	0.15	0.57
8OHG-T	219	6.06	0.20	0.93
8OHG-Na^{+}	219	7.87	0.24	1.02
8OHG-K^{+}	219	2.32	0.28	0.26
G-T	219	15.42	0.18	2.65
G-Na^{+}	219	6.48	0.15	1.30
G-K^{+}	219	7.06	0.17	1.26

Supplementary Table 5. The hydrogen-bond geometry for **6** (Å, °).

D-H-A	*d* (D-H)	*d* (H-A)	<DHA	*d* (D…A)
N2-H2A…O11	0.88	2.17	174	3.046
N2-H2B…O6	0.88	2.13	135	2.819
N1-H1…N7	0.88	2.04	163	2.896
O3'-H3'…O2'	0.84	2.28	111	2.704
O3'-H3'…O11	0.84	2.07	143	2.788
O5'-H5'…O11	0.84	1.91	159	2.711
O2'-H2'…O15	0.84	1.87	166	2.692
N8-H8A…O6	0.88	2.00	163	2.855
N8-H8B…O4'	0.89	2.57	129	3.207
N8-H8B…O5'	0.89	2.23	148	3.020
C1'-H1'…N3	1.00	2.53	106	2.973
C2'-H2'…O5'	1.00	2.59	112	3.096
C2'-H2'…N8	1.00	2.50	124	3.175
C13-H13A…O2'	0.98	2.54	146	3.396
C16-H16B…O5'	0.98	2.53	121	3.147

Supplementary Table 6. The pseudorotational phase angle and puckering amplitude of **6**. The intermolecular intermolecular hydrogen bonds (HBs), C2'-H2' ···O5' and C2'-H2'···N8, led to C2' folding in the endo direction, resulting in the sugar puckering mode exhibiting C2'-endo conformation.

Name	**6**
Conformer Torsion angle χ (O4′-C1′-N9-C4)	χ=-117.78°, anti
Sugar puckering	C2'-endo (P=155.29°, τm=40.40°)
Torsion angle γ (O5′-C5′-C4′-C3′)	γ=53.02°, +sc (gauche, gauche)

Supplementary Table 7. The crystallographic data of **6**.

Empirical formula	$C_{14}H_{26}N_6O_7S_2$
Formula weight	454.53
Temperature/K	150.0
Crystal system	orthorhombic
Space group	$P2_12_12_1$
a/Å	10.8433(14)
b/Å	10.9433(11)
c/Å	17.7572(12)
α/°	90
β/°	90
γ/°	90
Volume/Å^3	2107.1(4)
Z	4
ρ_{calc}g/cm^3	1.433
μ/mm^{-1}	0.301
F(000)	960.0
Crystal size/mm^3	0.12 × 0.04 × 0.02
Radiation	MoKα (λ = 0.71073)
2θ range for data collection/°	4.372 to 55.518
Index ranges	$-14 \le h \le 12$, $-14 \le k \le 11$, $-23 \le l \le 23$
Reflections collected	16381
Independent reflections	4859 [R_{int} = 0.0980, R_{sigma} = 0.1135]
Data/restraints/parameters	4859/0/271
Goodness-of-fit on F^2	1.050
Final R indexes [I>=2σ (I)]	R_1 = 0.0578, wR_2 = 0.0954
Final R indexes [all data]	R_1 = 0.1201, wR_2 = 0.1211
CCDC	2253566

Supplementary Table 8. Characteristics of the models constructed by LASSO and MLREM for feature selection.

	LR		RF		DT		XGBoost	
	Mean	SEM	Mean	SEM	Mean	SEM	Mean	SEM
LASSO								
Accuracy	0.70	0.01	0.64	0.01	0.68	0.01	0.67	0.02
F1 score	0.73	0.02	0.68	0.02	0.72	0.01	0.71	0.01
Precision	0.72	0.02	0.66	0.01	0.69	0.02	0.68	0.02
Recall	0.77	0.02	0.72	0.02	0.78	0.02	0.75	0.02
AUC	0.74	0.02	0.73	0.02	0.69	0.02	0.74	0.02
MLREM								
Accuracy	0.63	0.01	0.67	0.01	0.68	0.01	0.68	0.02
F1 score	0.69	0.01	0.70	0.00	0.71	0.01	0.71	0.02
Precision	0.63	0.01	0.68	0.01	0.70	0.01	0.69	0.01
Recall	0.79	0.02	0.75	0.02	0.74	0.02	0.75	0.02
AUC	0.67	0.02	0.75	0.02	0.96	0.02	0.75	0.02

Notes: *: Logistic regression (LR), decision tree (DT), random forest (RF), and extreme gradient boosting (XGBoost); Standard Error of the Mean (SEM)
#: LASSO: Least Absolute Shrinkage and Selection Operator, AUC: Area Under Curve, MLREM: Multiple linear regression with expectation maximization.

5. Supplementary References

1 Xu, P. *et al*. A nonswellable gradient hydrogel with tunable mechanical properties *J. Mater. Chem. B* **1**, 2702-2708 (2020).

2 Dhar, J., Swathi, K., Karothu, D. P., Narayan, K. S. & Patil, S. Modulation of Electronic and Self-Assembly Properties of a Donor–Acceptor–Donor-Based Molecular Materials via Atomistic Approach. *ACS Appl. Mater. Interfaces* **7**, 670-681 (2015).

3 Gupta, J. K., Adams, D. J. & Berry, N. G. Will it gel? Successful computational prediction of peptide gelators using physicochemical properties and molecular fingerprints. *Chem. Sci.* **7**, 4713-4719 (2016).

4 Li, F. *et al.* Design of self-assembly dipeptide hydrogels and machine learning via their chemical features. *Proc. Natl. Acad. Sci. U. S. A.* **116**, 11259-11264 (2019).

5 Van Lommel, R., Zhao, J., De Borggraeve, W. M., De Proft, F. & Alonso, M. Molecular dynamics based descriptors for predicting supramolecular gelation. *Chem. Sci.* **11**, 4226-4238 (2020).

6 Peters, G. M. *et al.* G4-Quartet·M+ Borate Hydrogels. *J. Am. Chem. Soc* **137**, 5819-5827 (2015).

7 Peters, G. M., Skala, L. P. & Davis, J. T. A Molecular Chaperone for G4-Quartet Hydrogels. *J. Am. Chem. Soc* **138**, 134-139 (2016).
