# Supplementary material for: Developing a machine learning model for accurate nucleoside hydrogels prediction based on descriptors
Source: Nat Commun. 2024 Mar 23;15:2603. doi: 10.1038/s41467-024-46866-9 (PMC10960799; doi:10.1038/s41467-024-46866-9)
Supplement: Supplementary file 3 — Description of Additional Supplementary Files [file 41467_2024_46866_MOESM3_ESM.pdf]

Description of Additional Supplementary Files

File Name: Supplementary Data 1
Description: Structure of 71 nucleoside derivatives.

File Name: Supplementary Data 2
Description: Structure of 71 nucleoside derivatives.

File Name: Supplementary Data 3
Description: The details of 5566 molecular descriptors.

File Name: Supplementary Data 4
Description: The optimal combination of descriptors of each algorithm.

File Name: Supplementary Data 5
Description: Information and feature importance of the optimal model features (LR with 24 descriptors).

File Name: Supplementary Data 6
Description: The feature importance of 70 molecular descriptors based on LASSO regression.

File Name: Supplementary Data 7
Description: The predictions of optimal model for nucleoside derivatives from PubChem database.

File Name: Supplementary Data 8
Description: The validation for the hydrogel-forming ability of the 24 nucleoside derivatives.

File Name: Supplementary Data 9
Description: Search terms and search strategy of system-wide search.

File Name: Supplementary Data 10
Description: The atomic coordinates of the optimized computational models.
