# Supplementary material for: Developing a machine learning model for accurate nucleoside hydrogels prediction based on descriptors
Source: Nat Commun. 2024 Mar 23;15:2603. doi: 10.1038/s41467-024-46866-9 (PMC10960799; doi:10.1038/s41467-024-46866-9)
Supplement: Supplementary file 11 — Supplementary Data 8 [file 41467_2024_46866_MOESM11_ESM.docx]

**Supplementary Data 8.** The validation for the hydrogel-forming ability of the 24 nucleoside derivatives

| No. | Nucleoside derivatives | | PMID | Prediction probability for hydrogel-forming ability | Rank for prediction probability | Test Result |
| --- | --- | --- | --- | --- | --- | --- |
| **1** | DTT* | 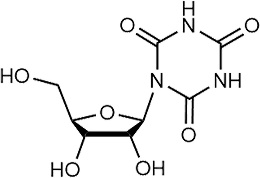 | 21826754 | 0.680 | 2 (0.1%) | Gel (+) |
| **2** | XTS | 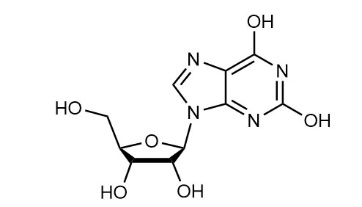 | 77518952 | 0.621 | 57 (0.7%) | Gel (-) |
| **3** | GMP | 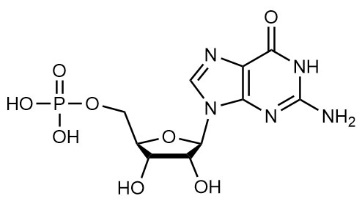 | 135398631 | 0.610 | 118 (1.6%) | Gel (+) |
| **4** | IMP | 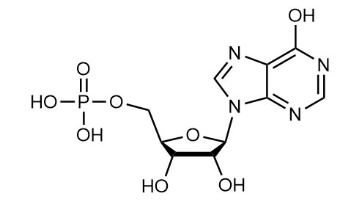 | 135398640 | 0.593 | 365 (5.0%) | Gel (+) |
| **5** | 5-FUR | 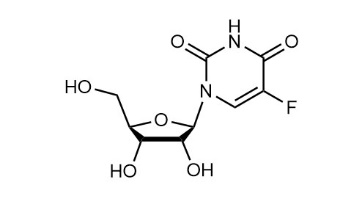 | 9427 | 0.591 | 409 (5.6%) | Gel (-) |
| **6** | 8-AG | 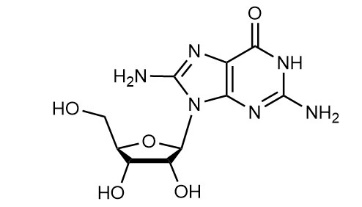 | 135518164 | 0.590 | 454 (6.3%) | Gel (+) |
| **7** | dGMP | 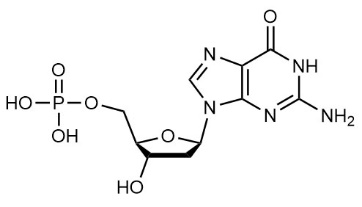 | 135596592 | 0.590 | 454 (6.3%) | Gel (+) |
| **8** | 8-OHG | 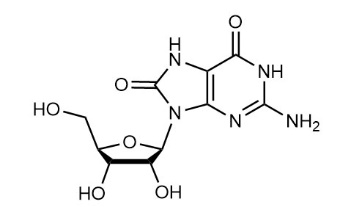 | 135407175 | 0.590 | 454 (6.3%) | Gel (+) |
| **9** | 8-AzaG | 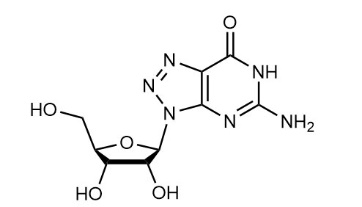 | 135763231 | 0.587 | 553 (7.6%) | Gel (+) |
| **10** | I-5’-CA | 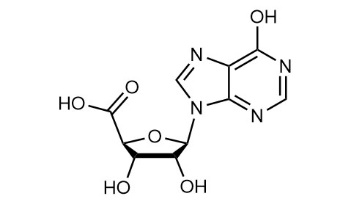 | 13542524 | 0.585 | 599 (8.3%) | Gel (+) |
| **11** | 2’-NH_2_-dG | 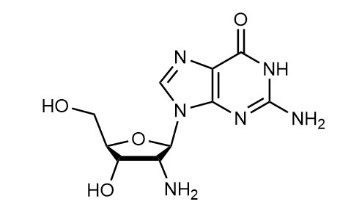 | 135491415 | 0.585 | 599 (8.3%) | Gel (+) |
| **12** | 2’-OMe-dG | 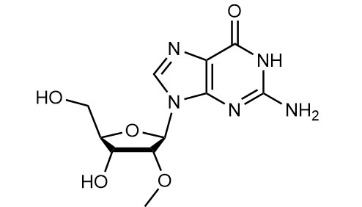 | 136441961 | 0.582 | 680 (9.4%) | Gel (+) |
| **13** | DRB | 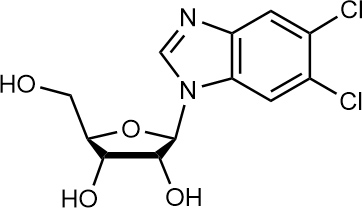 | 5894 | 0.452 | 6400 (88.2%) | Gel (+) |
| **14** | 9-THPA | 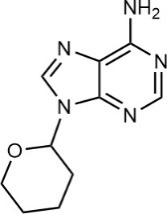 | 1932 | 0.449 | 6473 (89.2%) | Gel (-) |
| **15** | 9-THFA | 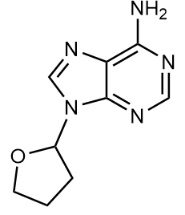 | 5270 | 0.448 | 6507 (89.7%) | Gel (-) |
| **16** | 2-TC | 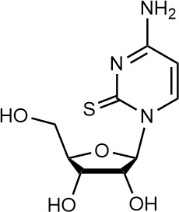 | 3011746 | 0.447 | 6534 (90.0%) | Gel (-) |
| **17** | 2’,3’-DA | 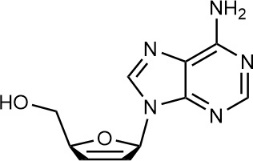 | 64975 | 0.444 | 6585 (90.7%) | Gel (-) |
| **18** | 2’,5’-DA | 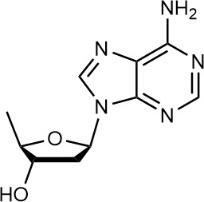 | 65166 | 0.443 | 6592 (90.8%) | Gel (-) |
| **19** | 2’-MeA | 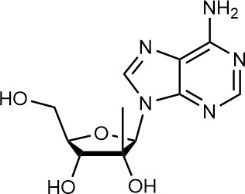 | 500900 | 0.430 | 6803 (93.7%) | Gel (-) |
| **20** | GCTB | 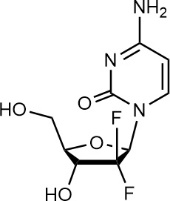 | 60750 | 0.424 | 6872 (94.7%) | Gel (-) |
| **21** | 2-ClA | 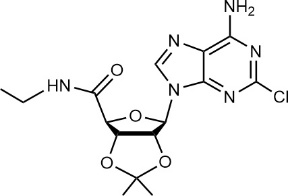 | 14775452 | 0.418 | 6949 (95.8%) | Gel (-) |
| **22** | 2-Cl-9-THPA | 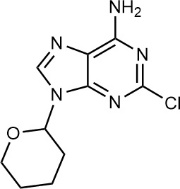 | 12777819 | 0.415 | 6980 (96.2%) | Gel (-) |
| **23** | 7-D-2’-MeA | 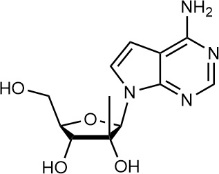 | 3011893 | 0.391 | 7144 (98.4%) | Gel (-) |
| **24** | 2’-MeC | 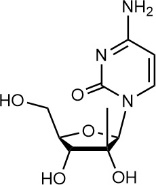 | 500902 | 0.379 | 7188 (99.1%) | Gel (+) |

Notes: *: **1**, 1-[3,4-Dihydroxy-5-(hydroxymethyl)oxolan-2-yl]-1,3,5-triazinane-2,4,6-trione, DTT; **2**, xanthosine, XTS; **3**, guanine 5'-monophosphate, GMP; **4**, inosine 5’-monophosphate, IMP; **5**, 5-fluorouridine, 5-FUR; **6**, 8-aminoguanosine, 8-AG; **7**, 2’-deoxyguanosine 5’-monophosphate, dGMP; **8**, 8-hydroxyguanosine, 8-OHG; **9**, 8-azaguanosine, 8-azaG; **10**, inosine-5’-carboxylic acid; I-5’-CA; **11**, 2’-amino-2’-deoxyguanosine, 2’-NH_2_-dG;**12**, 2’-O-Methylguanosine, 2’-OMe-dG; **13**, 5,6-Dichlorobenzimidazole riboside, DRB; **14**, 9-(2-tetrahydropyranyl)adenine, 9-THPA; **15**, 9-(2-tetrahydrofuryl)adenine, 9-THFA; **16**, 2-thiocytidine, 2-TC; **17**, 2’,3’-dideoxy-2’3’-didehydroadenosine, 2’,3’-DA; **18**, 2',5'-dideoxyadenosine, 2’,5’-DA; **19**, 2'-C-methyladenosine, 2’-MeA; **20**, gemcitabine, GCTB; **21**, 2-chloro-2',3'-O-isopropylideneadenosine-5'-N-ethylcarboxamide, 2-ClA; **22**, 2-chloro-9-(2-tetrahydropyranyl)adenine, 2-Cl-9-THPA; **23**, 7-deaza-2′-C-methyladenosine, 7-D-2’-MeA; **24**, 2'-C-methylcytidine, 2’-MeC**.**
