# Supplementary material for: Developing a machine learning model for accurate nucleoside hydrogels prediction based on descriptors
Source: Nat Commun. 2024 Mar 23;15:2603. doi: 10.1038/s41467-024-46866-9 (PMC10960799; doi:10.1038/s41467-024-46866-9)
Supplement: Supplementary file 12 — Supplementary Data 9 [file 41467_2024_46866_MOESM12_ESM.docx]

| **Supplementary Data 9. Search terms and search strategy of system-wide search** | | | |
| --- | --- | --- | --- |
| Part 1.Web of Science |  |  | |
| Description | Code | Search type | No. |
| Hydrogels | #1 | 'Hydrogels' OR 'Hydrogel' OR 'In Situ Hydrogels' OR 'In Situ Hydrogel' OR 'Hydrogel, In Situ' OR 'Patterned Hydrogels' OR 'Patterned Hydrogel' OR 'Hydrogel, Patterned' | 114,564 |
| Nucleosides | #2 | 'Nucleosides' OR 'Nucleoside' OR 'Nucleoside Analogs' OR 'Analogs, Nucleoside' OR 'Nucleoside Analog' OR 'Analog, Nucleoside' | 69,682 |
| Arabinonucleosides | #3 | 'Arabinonucleosides' OR 'Arabinofuranosylnucleosides' | 89 |
| Arabinofuranosyluracil | #4 | 'Arabinofuranosyluracil' OR 'Ara-U' OR 'Ara U' OR 'Uracil Arabinoside' OR 'Arabinoside, Uracil' OR 'Arauridine' OR 'Sponguridine' OR 'Uracil Arabinofuranoside' OR 'Arabinofuranoside, Uracil' OR '1-beta-D-Arabinofuranosyl Uracil' OR '1 beta D Arabinofuranosyl Uracil' OR 'Uracil, 1-beta-D-Arabinofuranosyl' OR 'Arabinosyluracil' OR 'NSC 68928' | 653 |
| Clofarabine | #5 | 'Clofarabine' OR '2-Chloro-9-(2-deoxy-2-fluoroarabinofuranosyl)adenine' OR 'Cl-F-ara-A' OR '2-Chloro-9-(2-deoxy-2-fluoro-beta-D-arbinofuranosyl)adenine' OR '2-Chloro-2'-arabino-fluoro-2'-deoxyadenosine' OR '2 Chloro 2' arabino fluoro 2' deoxyadenosine' OR '2-Chloro-2'-fluoroarabino-2'-deoxyadenosine' OR '2 Chloro 2' fluoroarabino 2' deoxyadenosine' OR '9H-Purin-6-amine, 2-chloro-9-(2-deoxy-2-fluoro-beta-D-arabinofuranosyl)-' OR 'Clolar' OR 'Evoltra' OR 'Clofarex' | 992 |
| Cytarabine | #6 | 'Cytarabine ' OR 'Arabinosylcytosine' OR 'Cytosine Arabinoside' OR 'Arabinoside, Cytosine' OR 'Arabinofuranosylcytosine' OR 'Aracytidine' OR 'beta-Ara C' OR 'beta Ara C' OR 'Cytarabine Hydrochloride' OR 'Cytosar' OR 'Cytosar-U' OR 'Cytosar U' OR 'Ara-C' OR 'Ara C' OR 'Aracytine' OR 'Cytonal' OR 'Ancitabine' OR 'Cyclocytidine' OR 'Anhydro-Ara-C' OR 'NSC-145,668' OR 'NSC 145,668' OR 'NSC145,668' OR 'NSC-145668' OR 'NSC 145668' OR 'NSC145668' OR 'U-33,624A' OR 'U 33,624A' OR 'U33,624A' OR 'U-33624A' OR 'U 33624A' OR 'U33624A' OR 'Cyclo-C' OR 'Cyclo C' OR 'CycloC' | 27,924 |
| Vidarabine | #7 | 'Vidarabine' OR 'beta-Ara A' OR 'beta Ara A' OR 'Adenine Arabinoside' OR 'Arabinoside, Adenine' OR 'alpha-D-Arabinofuranosyladenine' OR 'alpha D Arabinofuranosyladenine' OR 'Ara A' OR 'alpha-Ara A' OR 'alpha Ara A' OR 'Arabinofuranosyladenine' OR 'Arabinosyladenine' OR '9-beta-D-Arabinofuranosyladenine' OR '9 beta D Arabinofuranosyladenine' OR '9-beta-Arabinofuranosyladenine' OR '9 beta Arabinofuranosyladenine' OR 'Ara-A' OR 'Vira-A' OR 'Vira A' OR 'ViraA' | 11,610 |
| Arabinonucleosides combine | #8 | #3 OR #4 OR #5 OR #6 OR #7 | 35,174 |
| Deoxyribonucleosides | #9 | 'Deoxyribonucleosides' | 941 |
| Deoxyadenosines | #10 | 'Deoxyadenosines' OR 'Deoxyadenosine Derivatives' OR 'Derivatives, Deoxyadenosine' OR 'Adenine Deoxyribonucleosides' OR 'Deoxyribonucleosides, Adenine' OR 'Adenylyldeoxyribonucleosides' OR 'Cladribine' OR '2-Chlorodeoxyadenosine' OR '2-Chloro-2'-deoxyadenosine' OR '2'-Deoxy-2-chloroadenosine' OR 'Leustatin' OR 'Dideoxyadenosine' OR '2',3'-Dideoxyadenosine' OR '2',3' Dideoxyadenosine' OR 'ddA (Antiviral)' OR 'Puromycin Aminonucleoside' OR 'Aminonucleoside, Puromycin' OR '3'-Amino-3'-deoxy-N,N-dimethyladenosine' OR '3' Amino 3' deoxy N,N dimethyladenosine' OR 'Aminonucleoside' | 6,569 |
| Deoxycytidine | #11 | 'Deoxycytidine ' OR 'Bromodeoxycytidine' OR '5-Bromo-2'-Deoxycytidine' OR '5 Bromo 2' Deoxycytidine' OR 'Capecitabine' OR 'Emtricitabine' OR 'Beta-L-2',3'-dideoxy-5-fluoro-3'-thiacytidine' OR 'Beta L 2',3' dideoxy 5 fluoro 3' thiacytidine' OR 'Coviracil' OR 'Emtriva' OR 'Efavirenz, Emtricitabine, Tenofovir Disoproxil Fumarate Drug Combination' OR 'Atripla' OR 'Elvitegravir, Cobicistat, Emtricitabine, Tenofovir Disoproxil Fumarate Drug Combination' OR 'Elvitegravir, Cobicistat, Emtricitabine, and Tenofovir Disoproxil Fumarate' OR 'Elvitegravir-Cobicistat-Emtricitabine-Tenofovir Disoproxil Fumarate Drug Combination' OR 'Elvitegravir Cobicistat Emtricitabine Tenofovir Disoproxil Fumarate Drug Combination' OR 'Elvitegravir, Cobicistat, Emtricitabine, and Tenofovir Disoproxil Fumarate Drug Combination' OR 'Stribild' OR 'Genvoya' OR 'Quad Pill' OR 'Pill, Quad' OR 'Emtricitabine, Rilpivirine, Tenofovir Drug Combination' OR 'Emtricitabine Rilpivirine Tenofovir Drug Combination' OR 'Emtricitabine-Rilpivirine-Tenofovir Drug Combination' OR 'Combination, Emtricitabine-Rilpivirine-Tenofovir Drug' OR 'Drug Combination, Emtricitabine-Rilpivirine-Tenofovir' OR 'Complera' OR 'Emtricitabine, Tenofovir Disoproxil Fumarate Drug Combination' OR 'Emtricitabine-Tenofovir Disoproxil Fumarate Drug Combination' OR 'Emtricitabine Tenofovir Disoproxil Fumarate Drug Combination' OR 'Truvada' OR 'Zalcitabine' OR 'Dideoxycytidine' OR '2',3'-Dideoxycytidine' OR '2',3' Dideoxycytidine' OR 'ddC (Antiviral)' OR 'NSC-606170' OR 'NSC 606170' OR 'NSC606170' OR 'Hivid' OR 'HIVID Roche' OR 'Lamivudine' OR '3TC' OR '2',3'-Dideoxy-3'-thiacytidine' OR '2',3' Dideoxy 3' thiacytidine' OR 'Epivir' OR 'Lamivudine, (2S-cis)-Isomer' OR 'BCH-189' OR 'BCH 189' OR 'BCH189' OR 'GR-109714X' OR 'GR109714X' | 40,548 |
| Deoxyguanosine | #12 | 'Deoxyguanosine' OR '8-Hydroxy-2'-Deoxyguanosine' OR '8 Hydroxy 2' Deoxyguanosine' OR '8OHdG' OR '8-Hydroxydeoxyguanosine' OR '8 Hydroxydeoxyguanosine' OR '8-Oxo-2'-Deoxyguanosine' OR '8 Oxo 2' Deoxyguanosine' OR '2'-Deoxy-8-Oxoguanosine' OR '2' Deoxy 8 Oxoguanosine' OR '8-oxodG' OR '8-oxodGuo' OR '8-oxo-dG' OR '8-OH-dG' OR '8-Oxo-Deoxyguanosine' OR '8 Oxo Deoxyguanosine' OR '2'-Deoxy-8-Hydroxyguanosine' OR '2' Deoxy 8 Hydroxyguanosine' OR '8-oxo-dGuo' OR '8-Oxo-7-Hydrodeoxyguanosine' OR '8 Oxo 7 Hydrodeoxyguanosine' OR '7-Hydro-8-Oxodeoxyguanosine' OR '7 Hydro 8 Oxodeoxyguanosine' OR '8-Oxo-7,8-Dihydrodeoxyguanosine' OR '8 Oxo 7,8 Dihydrodeoxyguanosine' OR '2'-Deoxy-8-Oxo-7,8-Dihydroguanosine' OR '2' Deoxy 8 Oxo 7,8 Dihydroguanosine' OR '2'-Deoxy-7,8-Dihydro-8-Oxoguanosine' OR '2' Deoxy 7,8 Dihydro 8 Oxoguanosine' OR '7,8-Dihydro-8-Oxo-2'-Deoxyguanosine' OR '7,8 Dihydro 8 Oxo 2' Deoxyguanosine' OR '8-Oxo-7,8-Dihydro-2'-Deoxyguanosine' OR '8 Oxo 7,8 Dihydro 2' Deoxyguanosine' | 13,634 |
| Deoxyuridine | #13 | 'Deoxyuridine' OR 'Bromodeoxyuridine' OR 'Bromouracil Deoxyriboside' OR 'Deoxyriboside, Bromouracil' OR '5-Bromo-2'-deoxyuridine' OR '5 Bromo 2' deoxyuridine' OR 'BrdU' OR 'BUdR' OR '5-Bromodeoxyuridine' OR '5 Bromodeoxyuridine' OR 'Broxuridine' OR 'NSC-38297' OR 'Floxuridine' OR '5-FUdR' OR 'FUdR' OR 'Fluorodeoxyuridine' OR '5-Fluorodeoxyuridine' OR '5 Fluorodeoxyuridine' OR 'Idoxuridine' OR '5-Iodo-2'-deoxyuridine' OR '5 Iodo 2' deoxyuridine' OR 'IUdR' OR '5-Iododeoxyuridine' OR '5 Iododeoxyuridine' OR 'Iododeoxyuridine' OR 'Herplex Liquifilm' OR 'Liquifilm, Herplex' OR 'Idoxuridine, 123I-Labeled' OR '123I-Labeled Idoxuridine' OR 'Idoxuridine, 123I Labeled' OR 'Idoxuridine, 125I-Labeled' OR '125I-Labeled Idoxuridine' OR 'Idoxuridine, 125I Labeled' OR 'Idoxuridine, 131I-Labeled' OR '131I-Labeled Idoxuridine' OR 'Idoxuridine, 131I Labeled' OR 'Idoxuridine, 3H-Labeled' OR '3H-Labeled Idoxuridine' OR 'Idoxuridine, 3H Labeled' OR 'Stoxil' OR 'Idoxuridine, Radical Ion (1-)' OR 'Kerecide' OR 'NSC-39661' OR 'NSC 39661' OR 'NSC39661' OR 'Oftan-IDU' OR 'Oftan IDU' OR 'OftanIDU' OR 'SK and F-14287' OR 'Allergan 211' OR 'Idoxuridine, Radical Ion (+1)' | 32,335 |
| Dideoxynucleosides | #14 | 'Dideoxynucleosides' OR 'ddNus' OR '2',3'-Dideoxynucleosides' OR '2',3' Dideoxynucleosides' OR 'Dideoxyribonucleosides' OR 'Didanosine' OR 'Dideoxyinosine' OR '2',3'-Dideoxyinosine' OR '2',3' Dideoxyinosine' OR 'ddI (Antiviral)' OR 'Videx' OR 'NSC-612049' OR 'NSC 612049' OR 'NSC612049' OR 'Dideoxyadenosine' OR 'Stavudine' OR 'D4T' OR '2',3'-Didehydro-3'-deoxythymidine' OR '2',3' Didehydro 3' deoxythymidine' OR '2',3'-Didehydro-2',3'-dideoxythmidine' OR 'Zerit' OR 'BMY-27857' OR 'BMY 27857' OR 'BMY27857' OR 'Stavudine, Monosodium Salt' OR 'Zalcitabine' OR 'Dideoxycytidine' OR '2',3'-Dideoxycytidine' OR '2',3' Dideoxycytidine' OR 'ddC (Antiviral)' OR 'NSC-606170' OR 'NSC 606170' OR 'NSC606170' OR 'Hivid' OR 'HIVID Roche' OR 'Lamivudine' OR '3TC' OR '2',3'-Dideoxy-3'-thiacytidine' OR '2',3' Dideoxy 3' thiacytidine' OR 'Epivir' OR 'Lamivudine, (2S-cis)-Isomer' OR 'BCH-189' OR 'BCH 189' OR 'BCH189' OR 'GR-109714X' OR 'GR109714X' OR 'Zidovudine' OR 'Azidothymidine' OR 'AZT Antiviral' OR '3'-Azido-3'-deoxythymidine' OR '3' Azido 3' deoxythymidine' OR 'AZT (Antiviral)' OR '3'-Azido-2',3'-Dideoxythymidine' OR '3' Azido 2',3' Dideoxythymidine' OR 'AZT, Antiviral' OR 'Antiviral AZT' OR 'Retrovir' OR 'BW A509U' OR 'BWA-509U' OR 'BWA 509U' OR 'BWA509U' | 31,026 |
| Pentostatin | #15 | 'Pentostatin' OR 'Deoxycoformycin' OR 'Imidazo(4,5-d)(1,3)diazepin-8-ol, 3-(2-deoxy-beta-D-erythro-pentofuranosyl)-3,4,7,8-tetrahydro-, (R)-' OR '2'-Deoxycoformycin' OR '2' Deoxycoformycin' OR 'NSC-218321' OR 'NSC 218321' OR 'NSC218321' OR 'Co-Vidarabine' OR 'CI-825' OR 'CI 825' OR 'CI825' OR 'Nipent' | 2,915 |
| Thymidine | #16 | 'Thymidine' OR '2'-Deoxythymidine' OR '2' Deoxythymidine' OR 'Deoxythymidine' OR 'Stavudine' OR 'D4T' OR '2',3'-Didehydro-3'-deoxythymidine' OR '2',3' Didehydro 3' deoxythymidine' OR '2',3'-Didehydro-2',3'-dideoxythmidine' OR 'Zerit' OR 'BMY-27857' OR 'BMY 27857' OR 'BMY27857' OR 'Stavudine, Monosodium Salt' OR 'Telbivudine' OR 'Trifluridine' OR 'Trifluorothymidine' OR '2'-deoxy-5-(trifluoromethyl)uridine' OR 'Trifluoridine' OR '5-Trifluoromethyl-2'-deoxyuridine' OR '5 Trifluoromethyl 2' deoxyuridine' OR 'Triflumann' OR 'Virophta' OR 'Viroptic' OR 'TFT Ophtiole' OR 'Viromidin' OR 'Zidovudine' OR 'Azidothymidine' OR 'AZT Antiviral' OR '3'-Azido-3'-deoxythymidine' OR '3' Azido 3' deoxythymidine' OR 'AZT (Antiviral)' OR '3'-Azido-2',3'-Dideoxythymidine' OR '3' Azido 2',3' Dideoxythymidine' OR 'AZT, Antiviral' OR 'Antiviral AZT' OR 'Retrovir' OR 'BW A509U' OR 'BWA-509U' OR 'BWA 509U' OR 'BWA509U' | 62,025 |
| Deoxyribonucleosides conbine | #17 | #9 OR #10 OR #11 OR #12 OR #13 OR #14 OR #15 OR #16 | 147,740 |
| Purine Nucleosides | #18 | 'Purine Nucleosides' OR 'Nucleosides, Purine' OR 'Purine Nucleoside' OR 'Nucleoside, Purine' | 9,926 |
| Adenosine | #19 | TS=('Adenosine' OR 'Adenocard' OR 'Adenoscan' OR '2-Chloroadenosine' OR '2 Chloroadenosine' OR 'Cladribine' OR '2-Chlorodeoxyadenosine' OR '2-Chloro-2'-deoxyadenosine' OR '2'-Deoxy-2-chloroadenosine' OR 'Leustatin' OR 'Adenosine-5'-(N-ethylcarboxamide)' OR 'N6-Ethyl-carboxamido Adenosine' OR 'Adenosine, N6-Ethyl-carboxamido' OR 'N6 Ethyl carboxamido Adenosine' OR 'NECA' OR 'Adenosine-5'-N-ethyluronamide' OR 'Adenosine 5' N ethyluronamide' OR '5'-N-Ethylcarboxamidoadenosine' OR '5' N Ethylcarboxamidoadenosine' OR 'N-Ethylcarboxamidoadenosine' OR 'N Ethylcarboxamidoadenosine' OR 'Deoxyadenosines' OR 'Cladribine' OR '2-Chlorodeoxyadenosine' OR '2-Chloro-2'-deoxyadenosine' OR '2'-Deoxy-2-chloroadenosine' OR 'Leustatin' OR 'Dideoxyadenosine' OR '2',3'-Dideoxyadenosine' OR '2',3' Dideoxyadenosine' OR 'ddA (Antiviral)' OR 'Puromycin Aminonucleoside' OR 'Aminonucleoside, Puromycin' OR '3'-Amino-3'-deoxy-N,N-dimethyladenosine' OR '3' Amino 3' deoxy N,N dimethyladenosine' OR 'Aminonucleoside' OR 'Isopentenyladenosine' OR 'Phenylisopropyladenosine' OR 'N(6)-Phenylisopropyl-Adenosine' OR 'Isopropylphenyladenosine' OR 'L-Phenylisopropyladenosine' OR 'L Phenylisopropyladenosine' OR 'Puromycin' OR 'P-638' OR 'P 638' OR 'P638' OR 'Puromycin Hydrochloride' OR 'Stylomycin' OR 'CL-13900' OR 'CL 13900' OR 'CL13900' OR 'Puromycin Dihydrochloride' OR 'Puromycin Aminonucleoside' OR 'Aminonucleoside, Puromycin' OR '3'-Amino-3'-deoxy-N,N-dimethyladenosine' OR '3' Amino 3' deoxy N,N dimethyladenosine' OR 'Aminonucleoside' OR 'S-Adenosylhomocysteine' OR 'S Adenosylhomocysteine' OR 'Adenosylhomocysteine, S' OR 'S-Adenosylmethionine' OR 'S Adenosylmethionine' OR 'SAM-e' OR 'AdoMet' OR 'S-Adenosyl-L-Methionine' OR 'S Adenosyl L Methionine' OR 'Ademetionine' OR 'S Amet' OR 'Samyr' OR 'S-Adenosylmethionine Sulfate Tosylate' OR 'S Adenosylmethionine Sulfate Tosylate' OR 'Gumbaral' OR 'FO-1561' OR 'FO 1561' OR 'FO1561' OR 'Ticagrelor' OR 'Brilique' OR 'AZD 6140' OR 'AZD6140' OR 'AZD-6140' OR 'Brilinta' OR '3-(7-((2-(3,4-Difluorophenyl)cyclopropyl)amino)-5-(propylthio)-3H-(1-3)-triazolo(4,5-d)pyrimidin-3-yl)-5-(2-hydroxyethoxy)cyclopentane-1,2-diol' OR 'Vidarabine' OR 'beta-Ara A' OR 'beta Ara A' OR 'Adenine Arabinoside' OR 'Arabinoside, Adenine' OR 'alpha-D-Arabinofuranosyladenine' OR 'alpha D Arabinofuranosyladenine' OR 'Ara A' OR 'alpha-Ara A' OR 'alpha Ara A' OR 'Arabinofuranosyladenine' OR 'Arabinosyladenine' OR '9-beta-D-Arabinofuranosyladenine' OR '9 beta D Arabinofuranosyladenine' OR '9-beta-Arabinofuranosyladenine' OR '9 beta Arabinofuranosyladenine' OR 'Ara-A' OR 'Vira-A' OR 'Vira A' OR 'ViraA') | 181,428 |
| Guanosine | #20 | TS=('Guanosine' OR 'Deoxyguanosine' OR '8-Hydroxy-2'-Deoxyguanosine' OR '8 Hydroxy 2' Deoxyguanosine' OR '8OHdG' OR '8-Hydroxydeoxyguanosine' OR '8 Hydroxydeoxyguanosine' OR '8-Oxo-2'-Deoxyguanosine' OR '8 Oxo 2' Deoxyguanosine' OR '2'-Deoxy-8-Oxoguanosine' OR '2' Deoxy 8 Oxoguanosine' OR '8-oxodG' OR '8-oxodGuo' OR '8-oxo-dG' OR '8-OH-dG' OR '8-Oxo-Deoxyguanosine' OR '8 Oxo Deoxyguanosine' OR '2'-Deoxy-8-Hydroxyguanosine' OR '2' Deoxy 8 Hydroxyguanosine' OR '8-oxo-dGuo' OR '8-Oxo-7-Hydrodeoxyguanosine' OR '8 Oxo 7 Hydrodeoxyguanosine' OR '7-Hydro-8-Oxodeoxyguanosine' OR '7 Hydro 8 Oxodeoxyguanosine' OR '8-Oxo-7,8-Dihydrodeoxyguanosine' OR '8 Oxo 7,8 Dihydrodeoxyguanosine' OR '2'-Deoxy-8-Oxo-7,8-Dihydroguanosine' OR '2' Deoxy 8 Oxo 7,8 Dihydroguanosine' OR '2'-Deoxy-7,8-Dihydro-8-Oxoguanosine' OR '2' Deoxy 7,8 Dihydro 8 Oxoguanosine' OR '7,8-Dihydro-8-Oxo-2'-Deoxyguanosine' OR '7,8 Dihydro 8 Oxo 2' Deoxyguanosine' OR '8-Oxo-7,8-Dihydro-2'-Deoxyguanosine' OR '8 Oxo 7,8 Dihydro 2' Deoxyguanosine' OR 'Nucleoside Q' OR 'Queuosine' OR 'Q-Ribonucleoside' OR 'Q Ribonucleoside' OR 'Q Nucleoside' OR 'Nucleoside Q*') | 47,343 |
| Inosine | #21 | TS=('Inosine' OR 'Didanosine' OR 'Dideoxyinosine' OR '2',3'-Dideoxyinosine' OR '2',3' Dideoxyinosine' OR 'ddI (Antiviral)' OR 'Videx' OR 'NSC-612049' OR 'NSC 612049' OR 'NSC612049' OR 'Inosine Pranobex' OR 'Pranobex, Inosine' OR 'Metisoprinol' OR 'Methysoprinol' OR 'Inosiplex' OR 'Methisoprinol' OR 'Isoprinosin' OR 'Isoprinosine' OR 'NPT-10381' OR 'NPT 10381' OR 'NPT10381' OR 'Imunovir' OR 'Thioinosine' OR '6-Mercaptopurine Riboside' OR '6 Mercaptopurine Riboside' OR 'Riboside, 6-Mercaptopurine' OR 'Ribosyl-6-mercaptopurine' OR 'Ribosyl 6 mercaptopurine' OR 'NSC-4911' OR 'Methylthioinosine' OR '6-Methylmercaptopurine Riboside' OR '6 Methylmercaptopurine Riboside' OR 'Riboside, 6-Methylmercaptopurine' OR '6-Methylthiopurine Riboside' OR '6 Methylthiopurine Riboside' OR 'Riboside, 6-Methylthiopurine') | 12,480 |
| Tubercidin | #22 | TS=('Tubercidin' OR '4-Amino-7 beta-D-ribofuranosyl-7H-pyrrolo(2,3-d)pyrimidine' OR '7-Deazaadenosine' OR '7 Deazaadenosine' OR 'Deazaadenosine') | 804 |
| Purine Nucleosides combine | #23 | #17 OR #18 OR #19 OR #20 OR #21 OR #22 | 235,895 |
| Pyrimidine Nucleosides | #24 | TS=('Pyrimidine Nucleosides') | 6,637 |
| Cytidine | #25 | TS=('Cytidine' OR 'Cytosine Riboside' OR 'Riboside, Cytosine' OR 'Cytosine Ribonucleoside' OR 'Ribonucleoside, Cytosine' OR 'Azacitidine' OR '5-Azacytidine' OR '5 Azacytidine' OR 'Azacytidine' OR 'Vidaza' OR 'NSC-102816' OR 'NSC 102816' OR 'NSC102816' OR 'Decitabine' OR '5-Aza-2'-deoxycytidine' OR '5 Aza 2' deoxycytidine' OR '5-AzadC' OR 'AzadC Compound' OR 'Compound, AzadC' OR '5AzadC' OR '2'-Deoxy-5-azacytidine' OR '2' Deoxy 5 azacytidine' OR '5-Azadeoxycytidine' OR '5 Azadeoxycytidine' OR 'Dacogen' OR '5-Deoxyazacytidine' OR '5 Deoxyazacytidine' OR 'NSC 127716' OR 'NSC-127716' OR 'NSC127716' OR 'Decitabine Mesylate' OR 'Mesylate, Decitabine' OR 'Cytarabine' OR 'Arabinosylcytosine' OR 'Cytosine Arabinoside' OR 'Arabinoside, Cytosine' OR 'Arabinofuranosylcytosine' OR 'Aracytidine' OR 'beta-Ara C' OR 'beta Ara C' OR 'Cytarabine Hydrochloride' OR 'Cytosar' OR 'Cytosar-U' OR 'Cytosar U' OR 'Ara-C' OR 'Ara C' OR 'Aracytine' OR 'Cytonal' OR 'Ancitabine' OR 'Cyclocytidine' OR 'Anhydro-Ara-C' OR 'NSC-145,668' OR 'NSC 145,668' OR 'NSC145,668' OR 'NSC-145668' OR 'NSC 145668' OR 'NSC145668' OR 'U-33,624A' OR 'U 33,624A' OR 'U33,624A' OR 'U-33624A' OR 'U 33624A' OR 'U33624A' OR 'Cyclo-C' OR 'Cyclo C' OR 'CycloC' OR 'Deoxycytidine ' OR 'Bromodeoxycytidine' OR '5-Bromo-2'-Deoxycytidine' OR '5 Bromo 2' Deoxycytidine' OR 'Capecitabine' OR 'Emtricitabine' OR 'Beta-L-2',3'-dideoxy-5-fluoro-3'-thiacytidine' OR 'Beta L 2',3' dideoxy 5 fluoro 3' thiacytidine' OR 'Coviracil' OR 'Emtriva' OR 'Efavirenz, Emtricitabine, Tenofovir Disoproxil Fumarate Drug Combination' OR 'Atripla' OR 'Elvitegravir, Cobicistat, Emtricitabine, Tenofovir Disoproxil Fumarate Drug Combination' OR 'Elvitegravir, Cobicistat, Emtricitabine, and Tenofovir Disoproxil Fumarate' OR 'Elvitegravir-Cobicistat-Emtricitabine-Tenofovir Disoproxil Fumarate Drug Combination' OR 'Elvitegravir Cobicistat Emtricitabine Tenofovir Disoproxil Fumarate Drug Combination' OR 'Elvitegravir, Cobicistat, Emtricitabine, and Tenofovir Disoproxil Fumarate Drug Combination' OR 'Stribild' OR 'Genvoya' OR 'Quad Pill' OR 'Pill, Quad' OR 'Emtricitabine, Rilpivirine, Tenofovir Drug Combination' OR 'Emtricitabine Rilpivirine Tenofovir Drug Combination' OR 'Emtricitabine-Rilpivirine-Tenofovir Drug Combination' OR 'Combination, Emtricitabine-Rilpivirine-Tenofovir Drug' OR 'Drug Combination, Emtricitabine-Rilpivirine-Tenofovir' OR 'Complera' OR 'Emtricitabine, Tenofovir Disoproxil Fumarate Drug Combination' OR 'Emtricitabine-Tenofovir Disoproxil Fumarate Drug Combination' OR 'Emtricitabine Tenofovir Disoproxil Fumarate Drug Combination' OR 'Truvada' OR 'Zalcitabine' OR 'Dideoxycytidine' OR '2',3'-Dideoxycytidine' OR '2',3' Dideoxycytidine' OR 'ddC (Antiviral)' OR 'NSC-606170' OR 'NSC 606170' OR 'NSC606170' OR 'Hivid' OR 'HIVID Roche' OR 'Lamivudine' OR '3TC' OR '2',3'-Dideoxy-3'-thiacytidine' OR '2',3' Dideoxy 3' thiacytidine' OR 'Epivir' OR 'Lamivudine, (2S-cis)-Isomer' OR 'BCH-189' OR 'BCH 189' OR 'BCH189' OR 'GR-109714X' OR 'GR109714X') | 86,210 |
| Formycins | #26 | TS=('Formycins' OR 'Coformycin' OR 'Pentostatin' OR 'Deoxycoformycin' OR 'Imidazo(4,5-d)(1,3)diazepin-8-ol, 3-(2-deoxy-beta-D-erythro-pentofuranosyl)-3,4,7,8-tetrahydro-, (R)-' OR '2'-Deoxycoformycin' OR '2' Deoxycoformycin' OR 'NSC-218321' OR 'NSC 218321' OR 'NSC218321' OR 'Co-Vidarabine' OR 'CI-825' OR 'CI 825' OR 'CI825' OR 'Nipent') | 3,034 |
| Thymidine | #27 | TS=('Thymidine' OR '2'-Deoxythymidine' OR '2' Deoxythymidine' OR 'Deoxythymidine' OR 'Stavudine' OR 'D4T' OR '2',3'-Didehydro-3'-deoxythymidine' OR '2',3' Didehydro 3' deoxythymidine' OR '2',3'-Didehydro-2',3'-dideoxythmidine' OR 'Zerit' OR 'BMY-27857' OR 'BMY 27857' OR 'BMY27857' OR 'Stavudine, Monosodium Salt' OR 'Telbivudine' OR 'Trifluridine' OR 'Trifluorothymidine' OR '2'-deoxy-5-(trifluoromethyl)uridine' OR 'Trifluoridine' OR '5-Trifluoromethyl-2'-deoxyuridine' OR '5 Trifluoromethyl 2' deoxyuridine' OR 'Triflumann' OR 'Virophta' OR 'Viroptic' OR 'TFT Ophtiole' OR 'Viromidin' OR 'Zidovudine' OR 'Azidothymidine' OR 'AZT Antiviral' OR '3'-Azido-3'-deoxythymidine' OR '3' Azido 3' deoxythymidine' OR 'AZT (Antiviral)' OR '3'-Azido-2',3'-Dideoxythymidine' OR '3' Azido 2',3' Dideoxythymidine' OR 'AZT, Antiviral' OR 'Antiviral AZT' OR 'Retrovir' OR 'BW A509U' OR 'BWA-509U' OR 'BWA 509U' OR 'BWA509U') | 62,025 |
| Tunicamycin | #28 | TS=('Tunicamycin') | 4,676 |
| Uridine | #29 | TS=('Uridine' OR 'Allouridine' OR 'Allo-Uridine' OR 'Allo Uridine' OR '3-Deazauridine' OR '3 Deazauridine' OR '4-Hydroxy-1-(beta-D-Ribopentofuranosyl)-2-Pyridone' OR 'Arabinofuranosyluracil' OR 'Ara-U' OR 'Ara U' OR 'Uracil Arabinoside' OR 'Arabinoside, Uracil' OR 'Arauridine' OR 'Sponguridine' OR 'Uracil Arabinofuranoside' OR 'Arabinofuranoside, Uracil' OR '1-beta-D-Arabinofuranosyl Uracil' OR '1 beta D Arabinofuranosyl Uracil' OR 'Uracil, 1-beta-D-Arabinofuranosyl' OR 'Arabinosyluracil' OR 'NSC 68928' OR 'Azauridine' OR '2-beta-D-Ribofuranosyl-1,2,4-triazine-3,5(2H,4H)-dione' OR '6-Azauridine' OR '6 Azauridine' OR 'Deoxyuridine' OR 'Bromodeoxyuridine' OR 'Bromouracil Deoxyriboside' OR 'Deoxyriboside, Bromouracil' OR '5-Bromo-2'-deoxyuridine' OR '5 Bromo 2' deoxyuridine' OR 'BrdU' OR 'BUdR' OR '5-Bromodeoxyuridine' OR '5 Bromodeoxyuridine' OR 'Broxuridine' OR 'NSC-38297' OR 'Floxuridine' OR '5-FUdR' OR 'FUdR' OR 'Fluorodeoxyuridine' OR '5-Fluorodeoxyuridine' OR '5 Fluorodeoxyuridine' OR 'Idoxuridine' OR '5-Iodo-2'-deoxyuridine' OR '5 Iodo 2' deoxyuridine' OR 'IUdR' OR '5-Iododeoxyuridine' OR '5 Iododeoxyuridine' OR 'Iododeoxyuridine' OR 'Herplex Liquifilm' OR 'Liquifilm, Herplex' OR 'Idoxuridine, 123I-Labeled' OR '123I-Labeled Idoxuridine' OR 'Idoxuridine, 123I Labeled' OR 'Idoxuridine, 125I-Labeled' OR '125I-Labeled Idoxuridine' OR 'Idoxuridine, 125I Labeled' OR 'Idoxuridine, 131I-Labeled' OR '131I-Labeled Idoxuridine' OR 'Idoxuridine, 131I Labeled' OR 'Idoxuridine, 3H-Labeled' OR '3H-Labeled Idoxuridine' OR 'Idoxuridine, 3H Labeled' OR 'Stoxil' OR 'Idoxuridine, Radical Ion (1-)' OR 'Kerecide' OR 'NSC-39661' OR 'NSC 39661' OR 'NSC39661' OR 'Oftan-IDU' OR 'Oftan IDU' OR 'OftanIDU' OR 'SK and F-14287' OR 'Allergan 211' OR 'Idoxuridine, Radical Ion (+1)' OR 'Pseudouridine' OR 'Tetrahydrouridine' OR 'NSC-112907' OR 'NSC 112907' OR 'NSC112907' OR 'Thiouridine' OR '4-Thiouridine' OR '4 Thiouridine') | 51,634 |
| Pyrimidine Nucleosides combine | #30 | #24 OR #25 OR #26 OR #27 OR #28 OR #29 | 197,467 |
| Ribonucleosides | #31 | TS=('Ribonucleosides') | 1,809 |
| Adenosine | #32 | TS=('Adenosine' OR 'Adenocard' OR 'Adenoscan' OR '2-Chloroadenosine' OR '2 Chloroadenosine' OR 'Cladribine' OR '2-Chlorodeoxyadenosine' OR '2-Chloro-2'-deoxyadenosine' OR '2'-Deoxy-2-chloroadenosine' OR 'Leustatin' OR 'Adenosine-5'-(N-ethylcarboxamide)' OR 'N6-Ethyl-carboxamido Adenosine' OR 'Adenosine, N6-Ethyl-carboxamido' OR 'N6 Ethyl carboxamido Adenosine' OR 'NECA' OR 'Adenosine-5'-N-ethyluronamide' OR 'Adenosine 5' N ethyluronamide' OR '5'-N-Ethylcarboxamidoadenosine' OR '5' N Ethylcarboxamidoadenosine' OR 'N-Ethylcarboxamidoadenosine' OR 'N Ethylcarboxamidoadenosine' OR 'Deoxyadenosines' OR 'Cladribine' OR '2-Chlorodeoxyadenosine' OR '2-Chloro-2'-deoxyadenosine' OR '2'-Deoxy-2-chloroadenosine' OR 'Leustatin' OR 'Dideoxyadenosine' OR '2',3'-Dideoxyadenosine' OR '2',3' Dideoxyadenosine' OR 'ddA (Antiviral)' OR 'Puromycin Aminonucleoside' OR 'Aminonucleoside, Puromycin' OR '3'-Amino-3'-deoxy-N,N-dimethyladenosine' OR '3' Amino 3' deoxy N,N dimethyladenosine' OR 'Aminonucleoside' OR 'Isopentenyladenosine' OR 'Phenylisopropyladenosine' OR 'N(6)-Phenylisopropyl-Adenosine' OR 'Isopropylphenyladenosine' OR 'L-Phenylisopropyladenosine' OR 'L Phenylisopropyladenosine' OR 'Puromycin' OR 'P-638' OR 'P 638' OR 'P638' OR 'Puromycin Hydrochloride' OR 'Stylomycin' OR 'CL-13900' OR 'CL 13900' OR 'CL13900' OR 'Puromycin Dihydrochloride' OR 'Puromycin Aminonucleoside' OR 'Aminonucleoside, Puromycin' OR '3'-Amino-3'-deoxy-N,N-dimethyladenosine' OR '3' Amino 3' deoxy N,N dimethyladenosine' OR 'Aminonucleoside' OR 'S-Adenosylhomocysteine' OR 'S Adenosylhomocysteine' OR 'Adenosylhomocysteine, S' OR 'S-Adenosylmethionine' OR 'S Adenosylmethionine' OR 'SAM-e' OR 'AdoMet' OR 'S-Adenosyl-L-Methionine' OR 'S Adenosyl L Methionine' OR 'Ademetionine' OR 'S Amet' OR 'Samyr' OR 'S-Adenosylmethionine Sulfate Tosylate' OR 'S Adenosylmethionine Sulfate Tosylate' OR 'Gumbaral' OR 'FO-1561' OR 'FO 1561' OR 'FO1561' OR 'Ticagrelor' OR 'Brilique' OR 'AZD 6140' OR 'AZD6140' OR 'AZD-6140' OR 'Brilinta' OR '3-(7-((2-(3,4-Difluorophenyl)cyclopropyl)amino)-5-(propylthio)-3H-(1-3)-triazolo(4,5-d)pyrimidin-3-yl)-5-(2-hydroxyethoxy)cyclopentane-1,2-diol' OR 'Vidarabine' OR 'beta-Ara A' OR 'beta Ara A' OR 'Adenine Arabinoside' OR 'Arabinoside, Adenine' OR 'alpha-D-Arabinofuranosyladenine' OR 'alpha D Arabinofuranosyladenine' OR 'Ara A' OR 'alpha-Ara A' OR 'alpha Ara A' OR 'Arabinofuranosyladenine' OR 'Arabinosyladenine' OR '9-beta-D-Arabinofuranosyladenine' OR '9 beta D Arabinofuranosyladenine' OR '9-beta-Arabinofuranosyladenine' OR '9 beta Arabinofuranosyladenine' OR 'Ara-A' OR 'Vira-A' OR 'Vira A' OR 'ViraA') | 181,428 |
| Cytidine | #33 | TS=('Cytidine' OR 'Azacitidine' OR '5-Azacytidine' OR '5 Azacytidine' OR 'Azacytidine' OR 'Vidaza' OR 'NSC-102816' OR 'NSC 102816' OR 'NSC102816' OR 'Decitabine' OR '5-Aza-2'-deoxycytidine' OR '5 Aza 2' deoxycytidine' OR '5-AzadC' OR 'AzadC Compound' OR 'Compound, AzadC' OR '5AzadC' OR '2'-Deoxy-5-azacytidine' OR '2' Deoxy 5 azacytidine' OR '5-Azadeoxycytidine' OR '5 Azadeoxycytidine' OR 'Dacogen' OR '5-Deoxyazacytidine' OR '5 Deoxyazacytidine' OR 'NSC 127716' OR 'NSC-127716' OR 'NSC127716' OR 'Decitabine Mesylate' OR 'Mesylate, Decitabine') | 24,915 |
| Dichlororibofuranosylbenzimidazole | #34 | TS=('Dichlororibofuranosylbenzimidazole' OR 'Dichlorobenzimidazole Riboside' OR 'Riboside, Dichlorobenzimidazole' OR '5,6-Dichloro-1-beta-D-ribofuranosyl-1-H-benzimidazol' OR '5,6 Dichloro 1 beta D ribofuranosyl 1 H benzimidazol' OR 'DRB' OR '5,6-Dichloro-1-beta-ribofuranosylbenzimidazole' OR '5,6 Dichloro 1 beta ribofuranosylbenzimidazole') | 2,796 |
| Formycins | #35 | TS=('Formycins' OR 'Coformycin') | 164 |
| Guanosine | #36 | TS=('Guanosine' OR 'Nucleoside Q' OR 'Queuosine' OR 'Q-Ribonucleoside' OR 'Q Ribonucleoside' OR 'Q Nucleoside' OR 'Nucleoside Q*') | 34,809 |
| Inosine | #37 | TS=('Inosine' OR 'Didanosine' OR 'Dideoxyinosine' OR '2',3'-Dideoxyinosine' OR '2',3' Dideoxyinosine' OR 'ddI (Antiviral)' OR 'Videx' OR 'NSC-612049' OR 'NSC 612049' OR 'NSC612049' OR 'Inosine Pranobex' OR 'Pranobex, Inosine' OR 'Metisoprinol' OR 'Methysoprinol' OR 'Inosiplex' OR 'Methisoprinol' OR 'Isoprinosin' OR 'Isoprinosine' OR 'NPT-10381' OR 'NPT 10381' OR 'NPT10381' OR 'Imunovir' OR 'Thioinosine' OR '6-Mercaptopurine Riboside' OR '6 Mercaptopurine Riboside' OR 'Riboside, 6-Mercaptopurine' OR 'Ribosyl-6-mercaptopurine' OR 'Ribosyl 6 mercaptopurine' OR 'NSC-4911' OR 'Methylthioinosine' OR '6-Methylmercaptopurine Riboside' OR '6 Methylmercaptopurine Riboside' OR 'Riboside, 6-Methylmercaptopurine' OR '6-Methylthiopurine Riboside' OR '6 Methylthiopurine Riboside' OR 'Riboside, 6-Methylthiopurine') | 12,480 |
| Ribavirin | #38 | TS=('Ribavirin' OR 'Ribovirin' OR 'Tribavirin' OR 'Rebetol' OR 'Virazole' OR 'Vilona' OR 'Ribasphere' OR 'Viramide' OR 'Virazide' OR 'ICN-1229' OR 'ICN 1229' OR 'ICN1229' OR 'Ribamide' OR 'Ribamidil' OR 'Ribamidyl') | 25,842 |
| Showdomycin | #39 | TS=('Showdomycin') | 158 |
| Toyocamycin | #40 | TS=('Toyocamycin' OR 'Toyokamycin' OR 'Deazacyanoadenosine') | 244 |
| Tubercidin | #41 | TS=('Tubercidin' OR '4-Amino-7 beta-D-ribofuranosyl-7H-pyrrolo(2,3-d)pyrimidine' OR '7-Deazaadenosine' OR '7 Deazaadenosine' OR 'Deazaadenosine') | 804 |
| Uridine | #42 | TS=('3-Deazauridine' OR '3 Deazauridine' OR '4-Hydroxy-1-(beta-D-Ribopentofuranosyl)-2-Pyridone' OR 'Azauridine' OR '2-beta-D-Ribofuranosyl-1,2,4-triazine-3,5(2H,4H)-dione' OR '6-Azauridine' OR '6 Azauridine' OR 'Pseudouridine' OR 'Tetrahydrouridine' OR 'NSC-112907' OR 'NSC 112907' OR 'NSC112907' OR 'Thiouridine' OR '4-Thiouridine' OR '4 Thiouridine') | 3,195 |
| Ribonucleosides combine | #43 | #31 OR #32 OR #33 OR #34 OR #35 OR #36 OR #37 OR #38 OR #39 OR #40 OR #41 OR #42 | 272,871 |
| Thionucleosides | #44 | TS=('Thionucleosides') | 200 |
| Azathioprine | #45 | TS=('Azathioprine' OR 'Azothioprine' OR 'Imurel' OR 'Imuran' OR 'Immuran' OR 'Azathioprine Sodium' OR 'Sodium, Azathioprine' OR 'Azathioprine Sodium Salt' OR 'Azathioprine Sulfate' ) | 17,825 |
| Thioinosine | #46 | TS=('Thioinosine' OR '6-Mercaptopurine Riboside' OR '6 Mercaptopurine Riboside' OR 'Riboside, 6-Mercaptopurine' OR 'Ribosyl-6-mercaptopurine' OR 'Ribosyl 6 mercaptopurine' OR 'NSC-4911' OR 'Methylthioinosine' OR '6-Methylmercaptopurine Riboside' OR '6 Methylmercaptopurine Riboside' OR 'Riboside, 6-Methylmercaptopurine' OR '6-Methylthiopurine Riboside' OR '6 Methylthiopurine Riboside' OR 'Riboside, 6-Methylthiopurine' ) | 418 |
| Thiouridine | #47 | TS=('Thiouridine' OR '4-Thiouridine' OR '4 Thiouridine') | 849 |
| Thionucleosides combine | #48 | #44 OR #45 OR #46 OR #47 | 19,225 |
| combine | #49 | #2 OR #8 OR #17 OR #23 OR #30 OR #43 OR #48 | 488,766 |
| combine | #50 | #1 AND #49 and Articles (Document Types) | 511 |
| Part 2. Medline | | | |
| Hydrogels | #1 | 'Hydrogels' OR 'Hydrogel' OR 'In Situ Hydrogels' OR 'In Situ Hydrogel' OR 'Hydrogel, In Situ' OR 'Patterned Hydrogels' OR 'Patterned Hydrogel' OR 'Hydrogel, Patterned' | 57,548 |
| Nucleosides | #2 | 'Nucleosides' OR 'Nucleoside' OR 'Nucleoside Analogs' OR 'Analogs, Nucleoside' OR 'Nucleoside Analog' OR 'Analog, Nucleoside' | 271,567 |
| Arabinonucleosides | #3 | 'Arabinonucleosides' OR 'Arabinofuranosylnucleosides' | 21,004 |
| Arabinofuranosyluracil | #4 | 'Arabinofuranosyluracil' OR 'Ara-U' OR 'Ara U' OR 'Uracil Arabinoside' OR 'Arabinoside, Uracil' OR 'Arauridine' OR 'Sponguridine' OR 'Uracil Arabinofuranoside' OR 'Arabinofuranoside, Uracil' OR '1-beta-D-Arabinofuranosyl Uracil' OR '1 beta D Arabinofuranosyl Uracil' OR 'Uracil, 1-beta-D-Arabinofuranosyl' OR 'Arabinosyluracil' OR 'NSC 68928' | 784 |
| Clofarabine | #5 | 'Clofarabine' OR '2-Chloro-9-(2-deoxy-2-fluoroarabinofuranosyl)adenine' OR 'Cl-F-ara-A' OR '2-Chloro-9-(2-deoxy-2-fluoro-beta-D-arbinofuranosyl)adenine' OR '2-Chloro-2'-arabino-fluoro-2'-deoxyadenosine' OR '2 Chloro 2' arabino fluoro 2' deoxyadenosine' OR '2-Chloro-2'-fluoroarabino-2'-deoxyadenosine' OR '2 Chloro 2' fluoroarabino 2' deoxyadenosine' OR '9H-Purin-6-amine, 2-chloro-9-(2-deoxy-2-fluoro-beta-D-arabinofuranosyl)-' OR 'Clolar' OR 'Evoltra' OR 'Clofarex' | 607 |
| Cytarabine | #6 | 'Cytarabine ' OR 'Arabinosylcytosine' OR 'Cytosine Arabinoside' OR 'Arabinoside, Cytosine' OR 'Arabinofuranosylcytosine' OR 'Aracytidine' OR 'beta-Ara C' OR 'beta Ara C' OR 'Cytarabine Hydrochloride' OR 'Cytosar' OR 'Cytosar-U' OR 'Cytosar U' OR 'Ara-C' OR 'Ara C' OR 'Aracytine' OR 'Cytonal' OR 'Ancitabine' OR 'Cyclocytidine' OR 'Anhydro-Ara-C' OR 'NSC-145,668' OR 'NSC 145,668' OR 'NSC145,668' OR 'NSC-145668' OR 'NSC 145668' OR 'NSC145668' OR 'U-33,624A' OR 'U 33,624A' OR 'U33,624A' OR 'U-33624A' OR 'U 33624A' OR 'U33624A' OR 'Cyclo-C' OR 'Cyclo C' OR 'CycloC' | 21,716 |
| Vidarabine | #7 | 'Vidarabine' OR 'beta-Ara A' OR 'beta Ara A' OR 'Adenine Arabinoside' OR 'Arabinoside, Adenine' OR 'alpha-D-Arabinofuranosyladenine' OR 'alpha D Arabinofuranosyladenine' OR 'Ara A' OR 'alpha-Ara A' OR 'alpha Ara A' OR 'Arabinofuranosyladenine' OR 'Arabinosyladenine' OR '9-beta-D-Arabinofuranosyladenine' OR '9 beta D Arabinofuranosyladenine' OR '9-beta-Arabinofuranosyladenine' OR '9 beta Arabinofuranosyladenine' OR 'Ara-A' OR 'Vira-A' OR 'Vira A' OR 'ViraA' | 6,026 |
| Arabinonucleosides combine | #8 | #3 OR #4 OR #5 OR #6 OR #7 | 28,236 |
| Deoxyribonucleosides | #9 | 'Deoxyribonucleosides' | 106,294 |
| Deoxyadenosines | #10 | 'Deoxyadenosines' OR 'Deoxyadenosine Derivatives' OR 'Derivatives, Deoxyadenosine' OR 'Adenine Deoxyribonucleosides' OR 'Deoxyribonucleosides, Adenine' OR 'Adenylyldeoxyribonucleosides' OR 'Cladribine' OR '2-Chlorodeoxyadenosine' OR '2-Chloro-2'-deoxyadenosine' OR '2'-Deoxy-2-chloroadenosine' OR 'Leustatin' OR 'Dideoxyadenosine' OR '2',3'-Dideoxyadenosine' OR '2',3' Dideoxyadenosine' OR 'ddA (Antiviral)' OR 'Puromycin Aminonucleoside' OR 'Aminonucleoside, Puromycin' OR '3'-Amino-3'-deoxy-N,N-dimethyladenosine' OR '3' Amino 3' deoxy N,N dimethyladenosine' OR 'Aminonucleoside' | 11,342 |
| Deoxycytidine | #11 | 'Deoxycytidine ' OR 'Bromodeoxycytidine' OR '5-Bromo-2'-Deoxycytidine' OR '5 Bromo 2' Deoxycytidine' OR 'Capecitabine' OR 'Emtricitabine' OR 'Beta-L-2',3'-dideoxy-5-fluoro-3'-thiacytidine' OR 'Beta L 2',3' dideoxy 5 fluoro 3' thiacytidine' OR 'Coviracil' OR 'Emtriva' OR 'Efavirenz, Emtricitabine, Tenofovir Disoproxil Fumarate Drug Combination' OR 'Atripla' OR 'Elvitegravir, Cobicistat, Emtricitabine, Tenofovir Disoproxil Fumarate Drug Combination' OR 'Elvitegravir, Cobicistat, Emtricitabine, and Tenofovir Disoproxil Fumarate' OR 'Elvitegravir-Cobicistat-Emtricitabine-Tenofovir Disoproxil Fumarate Drug Combination' OR 'Elvitegravir Cobicistat Emtricitabine Tenofovir Disoproxil Fumarate Drug Combination' OR 'Elvitegravir, Cobicistat, Emtricitabine, and Tenofovir Disoproxil Fumarate Drug Combination' OR 'Stribild' OR 'Genvoya' OR 'Quad Pill' OR 'Pill, Quad' OR 'Emtricitabine, Rilpivirine, Tenofovir Drug Combination' OR 'Emtricitabine Rilpivirine Tenofovir Drug Combination' OR 'Emtricitabine-Rilpivirine-Tenofovir Drug Combination' OR 'Combination, Emtricitabine-Rilpivirine-Tenofovir Drug' OR 'Drug Combination, Emtricitabine-Rilpivirine-Tenofovir' OR 'Complera' OR 'Emtricitabine, Tenofovir Disoproxil Fumarate Drug Combination' OR 'Emtricitabine-Tenofovir Disoproxil Fumarate Drug Combination' OR 'Emtricitabine Tenofovir Disoproxil Fumarate Drug Combination' OR 'Truvada' OR 'Zalcitabine' OR 'Dideoxycytidine' OR '2',3'-Dideoxycytidine' OR '2',3' Dideoxycytidine' OR 'ddC (Antiviral)' OR 'NSC-606170' OR 'NSC 606170' OR 'NSC606170' OR 'Hivid' OR 'HIVID Roche' OR 'Lamivudine' OR '3TC' OR '2',3'-Dideoxy-3'-thiacytidine' OR '2',3' Dideoxy 3' thiacytidine' OR 'Epivir' OR 'Lamivudine, (2S-cis)-Isomer' OR 'BCH-189' OR 'BCH 189' OR 'BCH189' OR 'GR-109714X' OR 'GR109714X' | 10,731 |
| Deoxyguanosine | #12 | 'Deoxyguanosine' OR '8-Hydroxy-2'-Deoxyguanosine' OR '8 Hydroxy 2' Deoxyguanosine' OR '8OHdG' OR '8-Hydroxydeoxyguanosine' OR '8 Hydroxydeoxyguanosine' OR '8-Oxo-2'-Deoxyguanosine' OR '8 Oxo 2' Deoxyguanosine' OR '2'-Deoxy-8-Oxoguanosine' OR '2' Deoxy 8 Oxoguanosine' OR '8-oxodG' OR '8-oxodGuo' OR '8-oxo-dG' OR '8-OH-dG' OR '8-Oxo-Deoxyguanosine' OR '8 Oxo Deoxyguanosine' OR '2'-Deoxy-8-Hydroxyguanosine' OR '2' Deoxy 8 Hydroxyguanosine' OR '8-oxo-dGuo' OR '8-Oxo-7-Hydrodeoxyguanosine' OR '8 Oxo 7 Hydrodeoxyguanosine' OR '7-Hydro-8-Oxodeoxyguanosine' OR '7 Hydro 8 Oxodeoxyguanosine' OR '8-Oxo-7,8-Dihydrodeoxyguanosine' OR '8 Oxo 7,8 Dihydrodeoxyguanosine' OR '2'-Deoxy-8-Oxo-7,8-Dihydroguanosine' OR '2' Deoxy 8 Oxo 7,8 Dihydroguanosine' OR '2'-Deoxy-7,8-Dihydro-8-Oxoguanosine' OR '2' Deoxy 7,8 Dihydro 8 Oxoguanosine' OR '7,8-Dihydro-8-Oxo-2'-Deoxyguanosine' OR '7,8 Dihydro 8 Oxo 2' Deoxyguanosine' OR '8-Oxo-7,8-Dihydro-2'-Deoxyguanosine' OR '8 Oxo 7,8 Dihydro 2' Deoxyguanosine' | 15,133 |
| Deoxyuridine | #13 | 'Deoxyuridine' OR 'Bromodeoxyuridine' OR 'Bromouracil Deoxyriboside' OR 'Deoxyriboside, Bromouracil' OR '5-Bromo-2'-deoxyuridine' OR '5 Bromo 2' deoxyuridine' OR 'BrdU' OR 'BUdR' OR '5-Bromodeoxyuridine' OR '5 Bromodeoxyuridine' OR 'Broxuridine' OR 'NSC-38297' OR 'Floxuridine' OR '5-FUdR' OR 'FUdR' OR 'Fluorodeoxyuridine' OR '5-Fluorodeoxyuridine' OR '5 Fluorodeoxyuridine' OR 'Idoxuridine' OR '5-Iodo-2'-deoxyuridine' OR '5 Iodo 2' deoxyuridine' OR 'IUdR' OR '5-Iododeoxyuridine' OR '5 Iododeoxyuridine' OR 'Iododeoxyuridine' OR 'Herplex Liquifilm' OR 'Liquifilm, Herplex' OR 'Idoxuridine, 123I-Labeled' OR '123I-Labeled Idoxuridine' OR 'Idoxuridine, 123I Labeled' OR 'Idoxuridine, 125I-Labeled' OR '125I-Labeled Idoxuridine' OR 'Idoxuridine, 125I Labeled' OR 'Idoxuridine, 131I-Labeled' OR '131I-Labeled Idoxuridine' OR 'Idoxuridine, 131I Labeled' OR 'Idoxuridine, 3H-Labeled' OR '3H-Labeled Idoxuridine' OR 'Idoxuridine, 3H Labeled' OR 'Stoxil' OR 'Idoxuridine, Radical Ion (1-)' OR 'Kerecide' OR 'NSC-39661' OR 'NSC 39661' OR 'NSC39661' OR 'Oftan-IDU' OR 'Oftan IDU' OR 'OftanIDU' OR 'SK and F-14287' OR 'Allergan 211' OR 'Idoxuridine, Radical Ion (+1)' | 53,890 |
| Dideoxynucleosides | #14 | 'Dideoxynucleosides' OR 'ddNus' OR '2',3'-Dideoxynucleosides' OR '2',3' Dideoxynucleosides' OR 'Dideoxyribonucleosides' OR 'Didanosine' OR 'Dideoxyinosine' OR '2',3'-Dideoxyinosine' OR '2',3' Dideoxyinosine' OR 'ddI (Antiviral)' OR 'Videx' OR 'NSC-612049' OR 'NSC 612049' OR 'NSC612049' OR 'Dideoxyadenosine' OR 'Stavudine' OR 'D4T' OR '2',3'-Didehydro-3'-deoxythymidine' OR '2',3' Didehydro 3' deoxythymidine' OR '2',3'-Didehydro-2',3'-dideoxythmidine' OR 'Zerit' OR 'BMY-27857' OR 'BMY 27857' OR 'BMY27857' OR 'Stavudine, Monosodium Salt' OR 'Zalcitabine' OR 'Dideoxycytidine' OR '2',3'-Dideoxycytidine' OR '2',3' Dideoxycytidine' OR 'ddC (Antiviral)' OR 'NSC-606170' OR 'NSC 606170' OR 'NSC606170' OR 'Hivid' OR 'HIVID Roche' OR 'Lamivudine' OR '3TC' OR '2',3'-Dideoxy-3'-thiacytidine' OR '2',3' Dideoxy 3' thiacytidine' OR 'Epivir' OR 'Lamivudine, (2S-cis)-Isomer' OR 'BCH-189' OR 'BCH 189' OR 'BCH189' OR 'GR-109714X' OR 'GR109714X' OR 'Zidovudine' OR 'Azidothymidine' OR 'AZT Antiviral' OR '3'-Azido-3'-deoxythymidine' OR '3' Azido 3' deoxythymidine' OR 'AZT (Antiviral)' OR '3'-Azido-2',3'-Dideoxythymidine' OR '3' Azido 2',3' Dideoxythymidine' OR 'AZT, Antiviral' OR 'Antiviral AZT' OR 'Retrovir' OR 'BW A509U' OR 'BWA-509U' OR 'BWA 509U' OR 'BWA509U' | 20,767 |
| Pentostatin | #15 | 'Pentostatin' OR 'Deoxycoformycin' OR 'Imidazo(4,5-d)(1,3)diazepin-8-ol, 3-(2-deoxy-beta-D-erythro-pentofuranosyl)-3,4,7,8-tetrahydro-, (R)-' OR '2'-Deoxycoformycin' OR '2' Deoxycoformycin' OR 'NSC-218321' OR 'NSC 218321' OR 'NSC218321' OR 'Co-Vidarabine' OR 'CI-825' OR 'CI 825' OR 'CI825' OR 'Nipent' | 1,552 |
| Thymidine | #16 | 'Thymidine' OR '2'-Deoxythymidine' OR '2' Deoxythymidine' OR 'Deoxythymidine' OR 'Stavudine' OR 'D4T' OR '2',3'-Didehydro-3'-deoxythymidine' OR '2',3' Didehydro 3' deoxythymidine' OR '2',3'-Didehydro-2',3'-dideoxythmidine' OR 'Zerit' OR 'BMY-27857' OR 'BMY 27857' OR 'BMY27857' OR 'Stavudine, Monosodium Salt' OR 'Telbivudine' OR 'Trifluridine' OR 'Trifluorothymidine' OR '2'-deoxy-5-(trifluoromethyl)uridine' OR 'Trifluoridine' OR '5-Trifluoromethyl-2'-deoxyuridine' OR '5 Trifluoromethyl 2' deoxyuridine' OR 'Triflumann' OR 'Virophta' OR 'Viroptic' OR 'TFT Ophtiole' OR 'Viromidin' OR 'Zidovudine' OR 'Azidothymidine' OR 'AZT Antiviral' OR '3'-Azido-3'-deoxythymidine' OR '3' Azido 3' deoxythymidine' OR 'AZT (Antiviral)' OR '3'-Azido-2',3'-Dideoxythymidine' OR '3' Azido 2',3' Dideoxythymidine' OR 'AZT, Antiviral' OR 'Antiviral AZT' OR 'Retrovir' OR 'BW A509U' OR 'BWA-509U' OR 'BWA 509U' OR 'BWA509U' | 165,287 |
| Deoxyribonucleosides conbine | #17 | #9 OR #10 OR #11 OR #12 OR #13 OR #14 OR #15 OR #16 | 78,008 |
| Purine Nucleosides | #18 | 'Purine Nucleosides' OR 'Nucleosides, Purine' OR 'Purine Nucleoside' OR 'Nucleoside, Purine' | 9,926 |
| Adenosine | #19 | 'Adenosine' OR 'Adenocard' OR 'Adenoscan' OR '2-Chloroadenosine' OR '2 Chloroadenosine' OR 'Cladribine' OR '2-Chlorodeoxyadenosine' OR '2-Chloro-2'-deoxyadenosine' OR '2'-Deoxy-2-chloroadenosine' OR 'Leustatin' OR 'Adenosine-5'-(N-ethylcarboxamide)' OR 'N6-Ethyl-carboxamido Adenosine' OR 'Adenosine, N6-Ethyl-carboxamido' OR 'N6 Ethyl carboxamido Adenosine' OR 'NECA' OR 'Adenosine-5'-N-ethyluronamide' OR 'Adenosine 5' N ethyluronamide' OR '5'-N-Ethylcarboxamidoadenosine' OR '5' N Ethylcarboxamidoadenosine' OR 'N-Ethylcarboxamidoadenosine' OR 'N Ethylcarboxamidoadenosine' OR 'Deoxyadenosines' OR 'Cladribine' OR '2-Chlorodeoxyadenosine' OR '2-Chloro-2'-deoxyadenosine' OR '2'-Deoxy-2-chloroadenosine' OR 'Leustatin' OR 'Dideoxyadenosine' OR '2',3'-Dideoxyadenosine' OR '2',3' Dideoxyadenosine' OR 'ddA (Antiviral)' OR 'Puromycin Aminonucleoside' OR 'Aminonucleoside, Puromycin' OR '3'-Amino-3'-deoxy-N,N-dimethyladenosine' OR '3' Amino 3' deoxy N,N dimethyladenosine' OR 'Aminonucleoside' OR 'Isopentenyladenosine' OR 'Phenylisopropyladenosine' OR 'N(6)-Phenylisopropyl-Adenosine' OR 'Isopropylphenyladenosine' OR 'L-Phenylisopropyladenosine' OR 'L Phenylisopropyladenosine' OR 'Puromycin' OR 'P-638' OR 'P 638' OR 'P638' OR 'Puromycin Hydrochloride' OR 'Stylomycin' OR 'CL-13900' OR 'CL 13900' OR 'CL13900' OR 'Puromycin Dihydrochloride' OR 'Puromycin Aminonucleoside' OR 'Aminonucleoside, Puromycin' OR '3'-Amino-3'-deoxy-N,N-dimethyladenosine' OR '3' Amino 3' deoxy N,N dimethyladenosine' OR 'Aminonucleoside' OR 'S-Adenosylhomocysteine' OR 'S Adenosylhomocysteine' OR 'Adenosylhomocysteine, S' OR 'S-Adenosylmethionine' OR 'S Adenosylmethionine' OR 'SAM-e' OR 'AdoMet' OR 'S-Adenosyl-L-Methionine' OR 'S Adenosyl L Methionine' OR 'Ademetionine' OR 'S Amet' OR 'Samyr' OR 'S-Adenosylmethionine Sulfate Tosylate' OR 'S Adenosylmethionine Sulfate Tosylate' OR 'Gumbaral' OR 'FO-1561' OR 'FO 1561' OR 'FO1561' OR 'Ticagrelor' OR 'Brilique' OR 'AZD 6140' OR 'AZD6140' OR 'AZD-6140' OR 'Brilinta' OR '3-(7-((2-(3,4-Difluorophenyl)cyclopropyl)amino)-5-(propylthio)-3H-(1-3)-triazolo(4,5-d)pyrimidin-3-yl)-5-(2-hydroxyethoxy)cyclopentane-1,2-diol' OR 'Vidarabine' OR 'beta-Ara A' OR 'beta Ara A' OR 'Adenine Arabinoside' OR 'Arabinoside, Adenine' OR 'alpha-D-Arabinofuranosyladenine' OR 'alpha D Arabinofuranosyladenine' OR 'Ara A' OR 'alpha-Ara A' OR 'alpha Ara A' OR 'Arabinofuranosyladenine' OR 'Arabinosyladenine' OR '9-beta-D-Arabinofuranosyladenine' OR '9 beta D Arabinofuranosyladenine' OR '9-beta-Arabinofuranosyladenine' OR '9 beta Arabinofuranosyladenine' OR 'Ara-A' OR 'Vira-A' OR 'Vira A' OR 'ViraA' | 6,026 |
| Guanosine | #20 | 'Guanosine' OR 'Deoxyguanosine' OR '8-Hydroxy-2'-Deoxyguanosine' OR '8 Hydroxy 2' Deoxyguanosine' OR '8OHdG' OR '8-Hydroxydeoxyguanosine' OR '8 Hydroxydeoxyguanosine' OR '8-Oxo-2'-Deoxyguanosine' OR '8 Oxo 2' Deoxyguanosine' OR '2'-Deoxy-8-Oxoguanosine' OR '2' Deoxy 8 Oxoguanosine' OR '8-oxodG' OR '8-oxodGuo' OR '8-oxo-dG' OR '8-OH-dG' OR '8-Oxo-Deoxyguanosine' OR '8 Oxo Deoxyguanosine' OR '2'-Deoxy-8-Hydroxyguanosine' OR '2' Deoxy 8 Hydroxyguanosine' OR '8-oxo-dGuo' OR '8-Oxo-7-Hydrodeoxyguanosine' OR '8 Oxo 7 Hydrodeoxyguanosine' OR '7-Hydro-8-Oxodeoxyguanosine' OR '7 Hydro 8 Oxodeoxyguanosine' OR '8-Oxo-7,8-Dihydrodeoxyguanosine' OR '8 Oxo 7,8 Dihydrodeoxyguanosine' OR '2'-Deoxy-8-Oxo-7,8-Dihydroguanosine' OR '2' Deoxy 8 Oxo 7,8 Dihydroguanosine' OR '2'-Deoxy-7,8-Dihydro-8-Oxoguanosine' OR '2' Deoxy 7,8 Dihydro 8 Oxoguanosine' OR '7,8-Dihydro-8-Oxo-2'-Deoxyguanosine' OR '7,8 Dihydro 8 Oxo 2' Deoxyguanosine' OR '8-Oxo-7,8-Dihydro-2'-Deoxyguanosine' OR '8 Oxo 7,8 Dihydro 2' Deoxyguanosine' OR 'Nucleoside Q' OR 'Queuosine' OR 'Q-Ribonucleoside' OR 'Q Ribonucleoside' OR 'Q Nucleoside' OR 'Nucleoside Q*' | 317,244 |
| Inosine | #21 | 'Inosine' OR 'Didanosine' OR 'Dideoxyinosine' OR '2',3'-Dideoxyinosine' OR '2',3' Dideoxyinosine' OR 'ddI (Antiviral)' OR 'Videx' OR 'NSC-612049' OR 'NSC 612049' OR 'NSC612049' OR 'Inosine Pranobex' OR 'Pranobex, Inosine' OR 'Metisoprinol' OR 'Methysoprinol' OR 'Inosiplex' OR 'Methisoprinol' OR 'Isoprinosin' OR 'Isoprinosine' OR 'NPT-10381' OR 'NPT 10381' OR 'NPT10381' OR 'Imunovir' OR 'Thioinosine' OR '6-Mercaptopurine Riboside' OR '6 Mercaptopurine Riboside' OR 'Riboside, 6-Mercaptopurine' OR 'Ribosyl-6-mercaptopurine' OR 'Ribosyl 6 mercaptopurine' OR 'NSC-4911' OR 'Methylthioinosine' OR '6-Methylmercaptopurine Riboside' OR '6 Methylmercaptopurine Riboside' OR 'Riboside, 6-Methylmercaptopurine' OR '6-Methylthiopurine Riboside' OR '6 Methylthiopurine Riboside' OR 'Riboside, 6-Methylthiopurine' | 16,403 |
| Tubercidin | #22 | 'Tubercidin' OR '4-Amino-7 beta-D-ribofuranosyl-7H-pyrrolo(2,3-d)pyrimidine' OR '7-Deazaadenosine' OR '7 Deazaadenosine' OR 'Deazaadenosine' | 804 |
| Purine Nucleosides combine | #23 | #17 OR #18 OR #19 OR #20 OR #21 OR #22 | 368,237 |
| Pyrimidine Nucleosides | #24 | 'Pyrimidine Nucleosides' | 130,497 |
| Cytidine | #25 | 'Cytidine' OR 'Riboside, Cytosine' OR 'Cytosine Ribonucleoside' OR 'Ribonucleoside, Cytosine' OR 'Azacitidine' OR '5-Azacytidine' OR '5 Azacytidine' OR 'Azacytidine' OR 'Vidaza' OR 'NSC-102816' OR 'NSC 102816' OR 'NSC102816' OR 'Decitabine' OR '5-Aza-2'-deoxycytidine' OR '5 Aza 2' deoxycytidine' OR '5-AzadC' OR 'AzadC Compound' OR 'Compound, AzadC' OR '5AzadC' OR '2'-Deoxy-5-azacytidine' OR '2' Deoxy 5 azacytidine' OR '5-Azadeoxycytidine' OR '5 Azadeoxycytidine' OR 'Dacogen' OR '5-Deoxyazacytidine' OR '5 Deoxyazacytidine' OR 'NSC 127716' OR 'NSC-127716' OR 'NSC127716' OR 'Decitabine Mesylate' OR 'Mesylate, Decitabine' OR 'Cytarabine' OR 'Arabinosylcytosine' OR 'Cytosine Arabinoside' OR 'Arabinoside, Cytosine' OR 'Arabinofuranosylcytosine' OR 'Aracytidine' OR 'beta-Ara C' OR 'beta Ara C' OR 'Cytarabine Hydrochloride' OR 'Cytosar' OR 'Cytosar-U' OR 'Cytosar U' OR 'Ara-C' OR 'Ara C' OR 'Aracytine' OR 'Cytonal' OR 'Ancitabine' OR 'Cyclocytidine' OR 'Anhydro-Ara-C' OR 'NSC-145,668' OR 'NSC 145,668' OR 'NSC145,668' OR 'NSC-145668' OR 'NSC 145668' OR 'NSC145668' OR 'U-33,624A' OR 'U 33,624A' OR 'U33,624A' OR 'U-33624A' OR 'U 33624A' OR 'U33624A' OR 'Cyclo-C' OR 'Cyclo C' OR 'CycloC' OR 'Deoxycytidine ' OR 'Bromodeoxycytidine' OR '5-Bromo-2'-Deoxycytidine' OR '5 Bromo 2' Deoxycytidine' OR 'Capecitabine' OR 'Emtricitabine' OR 'Beta-L-2',3'-dideoxy-5-fluoro-3'-thiacytidine' OR 'Beta L 2',3' dideoxy 5 fluoro 3' thiacytidine' OR 'Coviracil' OR 'Emtriva' OR 'Efavirenz, Emtricitabine, Tenofovir Disoproxil Fumarate Drug Combination' OR 'Atripla' OR 'Elvitegravir, Cobicistat, Emtricitabine, Tenofovir Disoproxil Fumarate Drug Combination' OR 'Elvitegravir, Cobicistat, Emtricitabine, and Tenofovir Disoproxil Fumarate' OR 'Elvitegravir-Cobicistat-Emtricitabine-Tenofovir Disoproxil Fumarate Drug Combination' OR 'Elvitegravir Cobicistat Emtricitabine Tenofovir Disoproxil Fumarate Drug Combination' OR 'Elvitegravir, Cobicistat, Emtricitabine, and Tenofovir Disoproxil Fumarate Drug Combination' OR 'Stribild' OR 'Genvoya' OR 'Quad Pill' OR 'Pill, Quad' OR 'Emtricitabine, Rilpivirine, Tenofovir Drug Combination' OR 'Emtricitabine Rilpivirine Tenofovir Drug Combination' OR 'Emtricitabine-Rilpivirine-Tenofovir Drug Combination' OR 'Combination, Emtricitabine-Rilpivirine-Tenofovir Drug' OR 'Drug Combination, Emtricitabine-Rilpivirine-Tenofovir' OR 'Complera' OR 'Emtricitabine, Tenofovir Disoproxil Fumarate Drug Combination' OR 'Emtricitabine-Tenofovir Disoproxil Fumarate Drug Combination' OR 'Emtricitabine Tenofovir Disoproxil Fumarate Drug Combination' OR 'Truvada' OR 'Zalcitabine' OR 'Dideoxycytidine' OR '2',3'-Dideoxycytidine' OR '2',3' Dideoxycytidine' OR 'ddC (Antiviral)' OR 'NSC-606170' OR 'NSC 606170' OR 'NSC606170' OR 'Hivid' OR 'HIVID Roche' OR 'Lamivudine' OR '3TC' OR '2',3'-Dideoxy-3'-thiacytidine' OR '2',3' Dideoxy 3' thiacytidine' OR 'Epivir' OR 'Lamivudine, (2S-cis)-Isomer' OR 'BCH-189' OR 'BCH 189' OR 'BCH189' OR 'GR-109714X' OR 'GR109714X' | 10,731 |
| Formycins | #26 | 'Formycins' OR 'Coformycin' OR 'Pentostatin' OR 'Deoxycoformycin' OR 'Imidazo(4,5-d)(1,3)diazepin-8-ol, 3-(2-deoxy-beta-D-erythro-pentofuranosyl)-3,4,7,8-tetrahydro-, (R)-' OR '2'-Deoxycoformycin' OR '2' Deoxycoformycin' OR 'NSC-218321' OR 'NSC 218321' OR 'NSC218321' OR 'Co-Vidarabine' OR 'CI-825' OR 'CI 825' OR 'CI825' OR 'Nipent' | 1,552 |
| Thymidine | #27 | 'Thymidine' OR '2'-Deoxythymidine' OR '2' Deoxythymidine' OR 'Deoxythymidine' OR 'Stavudine' OR 'D4T' OR '2',3'-Didehydro-3'-deoxythymidine' OR '2',3' Didehydro 3' deoxythymidine' OR '2',3'-Didehydro-2',3'-dideoxythmidine' OR 'Zerit' OR 'BMY-27857' OR 'BMY 27857' OR 'BMY27857' OR 'Stavudine, Monosodium Salt' OR 'Telbivudine' OR 'Trifluridine' OR 'Trifluorothymidine' OR '2'-deoxy-5-(trifluoromethyl)uridine' OR 'Trifluoridine' OR '5-Trifluoromethyl-2'-deoxyuridine' OR '5 Trifluoromethyl 2' deoxyuridine' OR 'Triflumann' OR 'Virophta' OR 'Viroptic' OR 'TFT Ophtiole' OR 'Viromidin' OR 'Zidovudine' OR 'Azidothymidine' OR 'AZT Antiviral' OR '3'-Azido-3'-deoxythymidine' OR '3' Azido 3' deoxythymidine' OR 'AZT (Antiviral)' OR '3'-Azido-2',3'-Dideoxythymidine' OR '3' Azido 2',3' Dideoxythymidine' OR 'AZT, Antiviral' OR 'Antiviral AZT' OR 'Retrovir' OR 'BW A509U' OR 'BWA-509U' OR 'BWA 509U' OR 'BWA509U' | 6,474 |
| Tunicamycin | #28 | 'Tunicamycin' | 56,609 |
| Uridine | #29 | 'Uridine' OR 'Allouridine' OR 'Allo-Uridine' OR 'Allo Uridine' OR '3-Deazauridine' OR '3 Deazauridine' OR '4-Hydroxy-1-(beta-D-Ribopentofuranosyl)-2-Pyridone' OR 'Arabinofuranosyluracil' OR 'Ara-U' OR 'Ara U' OR 'Uracil Arabinoside' OR 'Arabinoside, Uracil' OR 'Arauridine' OR 'Sponguridine' OR 'Uracil Arabinofuranoside' OR 'Arabinofuranoside, Uracil' OR '1-beta-D-Arabinofuranosyl Uracil' OR '1 beta D Arabinofuranosyl Uracil' OR 'Uracil, 1-beta-D-Arabinofuranosyl' OR 'Arabinosyluracil' OR 'NSC 68928' OR 'Azauridine' OR '2-beta-D-Ribofuranosyl-1,2,4-triazine-3,5(2H,4H)-dione' OR '6-Azauridine' OR '6 Azauridine' OR 'Deoxyuridine' OR 'Bromodeoxyuridine' OR 'Bromouracil Deoxyriboside' OR 'Deoxyriboside, Bromouracil' OR '5-Bromo-2'-deoxyuridine' OR '5 Bromo 2' deoxyuridine' OR 'BrdU' OR 'BUdR' OR '5-Bromodeoxyuridine' OR '5 Bromodeoxyuridine' OR 'Broxuridine' OR 'NSC-38297' OR 'Floxuridine' OR '5-FUdR' OR 'FUdR' OR 'Fluorodeoxyuridine' OR '5-Fluorodeoxyuridine' OR '5 Fluorodeoxyuridine' OR 'Idoxuridine' OR '5-Iodo-2'-deoxyuridine' OR '5 Iodo 2' deoxyuridine' OR 'IUdR' OR '5-Iododeoxyuridine' OR '5 Iododeoxyuridine' OR 'Iododeoxyuridine' OR 'Herplex Liquifilm' OR 'Liquifilm, Herplex' OR 'Idoxuridine, 123I-Labeled' OR '123I-Labeled Idoxuridine' OR 'Idoxuridine, 123I Labeled' OR 'Idoxuridine, 125I-Labeled' OR '125I-Labeled Idoxuridine' OR 'Idoxuridine, 125I Labeled' OR 'Idoxuridine, 131I-Labeled' OR '131I-Labeled Idoxuridine' OR 'Idoxuridine, 131I Labeled' OR 'Idoxuridine, 3H-Labeled' OR '3H-Labeled Idoxuridine' OR 'Idoxuridine, 3H Labeled' OR 'Stoxil' OR 'Idoxuridine, Radical Ion (1-)' OR 'Kerecide' OR 'NSC-39661' OR 'NSC 39661' OR 'NSC39661' OR 'Oftan-IDU' OR 'Oftan IDU' OR 'OftanIDU' OR 'SK and F-14287' OR 'Allergan 211' OR 'Idoxuridine, Radical Ion (+1)' OR 'Pseudouridine' OR 'Tetrahydrouridine' OR 'NSC-112907' OR 'NSC 112907' OR 'NSC112907' OR 'Thiouridine' OR '4-Thiouridine' OR '4 Thiouridine' | 14,429 |
| Pyrimidine Nucleosides combine | #30 | #24 OR #25 OR #26 OR #27 OR #28 OR #29 | 185,187 |
| Ribonucleosides | #31 | 'Ribonucleosides' | 91,868 |
| Adenosine | #32 | 'Adenosine' OR 'Adenocard' OR '2-Chloroadenosine' OR '2 Chloroadenosine' OR 'Cladribine' OR '2-Chlorodeoxyadenosine' OR '2-Chloro-2'-deoxyadenosine' OR '2'-Deoxy-2-chloroadenosine' OR 'Leustatin' OR 'Adenosine-5'-(N-ethylcarboxamide)' OR 'N6-Ethyl-carboxamido Adenosine' OR 'Adenosine, N6-Ethyl-carboxamido' OR 'N6 Ethyl carboxamido Adenosine' OR 'NECA' OR 'Adenosine-5'-N-ethyluronamide' OR 'Adenosine 5' N ethyluronamide' OR '5'-N-Ethylcarboxamidoadenosine' OR '5' N Ethylcarboxamidoadenosine' OR 'N-Ethylcarboxamidoadenosine' OR 'N Ethylcarboxamidoadenosine' OR 'Deoxyadenosines' OR 'Cladribine' OR '2-Chlorodeoxyadenosine' OR '2-Chloro-2'-deoxyadenosine' OR '2'-Deoxy-2-chloroadenosine' OR 'Leustatin' OR 'Dideoxyadenosine' OR '2',3'-Dideoxyadenosine' OR '2',3' Dideoxyadenosine' OR 'ddA (Antiviral)' OR 'Puromycin Aminonucleoside' OR 'Aminonucleoside, Puromycin' OR '3'-Amino-3'-deoxy-N,N-dimethyladenosine' OR '3' Amino 3' deoxy N,N dimethyladenosine' OR 'Aminonucleoside' OR 'Isopentenyladenosine' OR 'Phenylisopropyladenosine' OR 'N(6)-Phenylisopropyl-Adenosine' OR 'Isopropylphenyladenosine' OR 'L-Phenylisopropyladenosine' OR 'L Phenylisopropyladenosine' OR 'Puromycin' OR 'P-638' OR 'P 638' OR 'P638' OR 'Puromycin Hydrochloride' OR 'Stylomycin' OR 'CL-13900' OR 'CL 13900' OR 'CL13900' OR 'Puromycin Dihydrochloride' OR 'Puromycin Aminonucleoside' OR 'Aminonucleoside, Puromycin' OR '3'-Amino-3'-deoxy-N,N-dimethyladenosine' OR '3' Amino 3' deoxy N,N dimethyladenosine' OR 'Aminonucleoside' OR 'S-Adenosylhomocysteine' OR 'S Adenosylhomocysteine' OR 'Adenosylhomocysteine, S' OR 'S-Adenosylmethionine' OR 'S Adenosylmethionine' OR 'SAM-e' OR 'AdoMet' OR 'S-Adenosyl-L-Methionine' OR 'S Adenosyl L Methionine' OR 'Ademetionine' OR 'S Amet' OR 'Samyr' OR 'S-Adenosylmethionine Sulfate Tosylate' OR 'S Adenosylmethionine Sulfate Tosylate' OR 'Gumbaral' OR 'FO-1561' OR 'FO 1561' OR 'FO1561' OR 'Ticagrelor' OR 'Brilique' OR 'AZD 6140' OR 'AZD6140' OR 'AZD-6140' OR 'Brilinta' OR '3-(7-((2-(3,4-Difluorophenyl)cyclopropyl)amino)-5-(propylthio)-3H-(1-3)-triazolo(4,5-d)pyrimidin-3-yl)-5-(2-hydroxyethoxy)cyclopentane-1,2-diol' OR 'Vidarabine' OR 'beta-Ara A' OR 'beta Ara A' OR 'Adenine Arabinoside' OR 'Arabinoside, Adenine' OR 'alpha-D-Arabinofuranosyladenine' OR 'alpha D Arabinofuranosyladenine' OR 'Ara A' OR 'alpha-Ara A' OR 'alpha Ara A' OR 'Arabinofuranosyladenine' OR 'Arabinosyladenine' OR '9-beta-D-Arabinofuranosyladenine' OR '9 beta D Arabinofuranosyladenine' OR '9-beta-Arabinofuranosyladenine' OR '9 beta Arabinofuranosyladenine' OR 'Ara-A' OR 'Vira-A' OR 'Vira A' OR 'ViraA' | 6,026 |
| Cytidine | #33 | 'Cytidine' OR 'Azacitidine' OR '5-Azacytidine' OR '5 Azacytidine' OR 'Azacytidine' OR 'Vidaza' OR 'NSC-102816' OR 'NSC 102816' OR 'NSC102816' OR 'Decitabine' OR '5-Aza-2'-deoxycytidine' OR '5 Aza 2' deoxycytidine' OR '5-AzadC' OR 'AzadC Compound' OR 'Compound, AzadC' OR '5AzadC' OR '2'-Deoxy-5-azacytidine' OR '2' Deoxy 5 azacytidine' OR '5-Azadeoxycytidine' OR '5 Azadeoxycytidine' OR 'Dacogen' OR '5-Deoxyazacytidine' OR '5 Deoxyazacytidine' OR 'NSC 127716' OR 'NSC-127716' OR 'NSC127716' OR 'Decitabine Mesylate' OR 'Mesylate, Decitabine' | 69,734 |
| Dichlororibofuranosylbenzimidazole | #34 | 'Dichlororibofuranosylbenzimidazole' OR 'Dichlorobenzimidazole Riboside' OR 'Riboside, Dichlorobenzimidazole' OR '5,6-Dichloro-1-beta-D-ribofuranosyl-1-H-benzimidazol' OR '5,6 Dichloro 1 beta D ribofuranosyl 1 H benzimidazol' OR 'DRB' OR '5,6-Dichloro-1-beta-ribofuranosylbenzimidazole' OR '5,6 Dichloro 1 beta ribofuranosylbenzimidazole' | 2,732 |
| Formycins | #35 | 'Formycins' OR 'Coformycin' | 1,499 |
| Guanosine | #36 | 'Guanosine' OR 'Nucleoside Q' OR 'Queuosine' OR 'Q-Ribonucleoside' OR 'Q Ribonucleoside' OR 'Q Nucleoside' OR 'Nucleoside Q*' | 310,762 |
| Inosine | #37 | 'Inosine' OR 'Didanosine' OR 'Dideoxyinosine' OR '2',3'-Dideoxyinosine' OR '2',3' Dideoxyinosine' OR 'ddI (Antiviral)' OR 'Videx' OR 'NSC-612049' OR 'NSC 612049' OR 'NSC612049' OR 'Inosine Pranobex' OR 'Pranobex, Inosine' OR 'Metisoprinol' OR 'Methysoprinol' OR 'Inosiplex' OR 'Methisoprinol' OR 'Isoprinosin' OR 'Isoprinosine' OR 'NPT-10381' OR 'NPT 10381' OR 'NPT10381' OR 'Imunovir' OR 'Thioinosine' OR '6-Mercaptopurine Riboside' OR '6 Mercaptopurine Riboside' OR 'Riboside, 6-Mercaptopurine' OR 'Ribosyl-6-mercaptopurine' OR 'Ribosyl 6 mercaptopurine' OR 'NSC-4911' OR 'Methylthioinosine' OR '6-Methylmercaptopurine Riboside' OR '6 Methylmercaptopurine Riboside' OR 'Riboside, 6-Methylmercaptopurine' OR '6-Methylthiopurine Riboside' OR '6 Methylthiopurine Riboside' OR 'Riboside, 6-Methylthiopurine' | 18,052 |
| Ribavirin | #38 | 'Ribavirin' OR 'Ribovirin' OR 'Tribavirin' OR 'Rebetol' OR 'Virazole' OR 'Vilona' OR 'Ribasphere' OR 'Viramide' OR 'ICN-1229' OR 'ICN 1229' OR 'ICN1229' OR 'Ribamide' OR 'Ribamidil' OR 'Ribamidyl' | 105 |
| Showdomycin | #39 | 'Showdomycin' | 215 |
| Toyocamycin | #40 | 'Toyocamycin' OR 'Toyokamycin' OR 'Deazacyanoadenosine' | 3,096 |
| Tubercidin | #41 | 'Tubercidin' OR '7-Deazaadenosine' OR '7 Deazaadenosine' OR 'Deazaadenosine' | 3,096 |
| Uridine | #42 | '3-Deazauridine' OR '3 Deazauridine' OR '4-Hydroxy-1-(beta-D-Ribopentofuranosyl)-2-Pyridone' OR 'Azauridine' OR '2-beta-D-Ribofuranosyl-1,2,4-triazine-3,5(2H,4H)-dione' OR '6-Azauridine' OR '6 Azauridine' OR 'Pseudouridine' OR 'Tetrahydrouridine' OR 'NSC-112907' OR 'NSC 112907' OR 'NSC112907' OR 'Thiouridine' OR '4-Thiouridine' OR '4 Thiouridine' | 1,018 |
| Ribonucleosides combine | #43 | #31 OR #32 OR #33 OR #34 OR #35 OR #36 OR #37 OR #38 OR #39 OR #40 OR #41 OR #42 | 335,131 |
| Thionucleosides | #44 | 'Thionucleosides' | 17,512 |
| Azathioprine | #45 | 'Azathioprine' OR 'Azothioprine' OR 'Imurel' OR 'Imuran' OR 'Immuran' OR 'Azathioprine Sodium' OR 'Sodium, Azathioprine' OR 'Azathioprine Sodium Salt' OR 'Azathioprine Sulfate' | 24,661 |
| Thioinosine | #46 | 'Thioinosine' OR '6-Mercaptopurine Riboside' OR '6 Mercaptopurine Riboside' OR 'Riboside, 6-Mercaptopurine' OR 'Ribosyl-6-mercaptopurine' OR 'Ribosyl 6 mercaptopurine' OR 'NSC-4911' OR 'Methylthioinosine' OR '6-Methylmercaptopurine Riboside' OR '6 Methylmercaptopurine Riboside' OR 'Riboside, 6-Methylmercaptopurine' OR '6-Methylthiopurine Riboside' OR '6 Methylthiopurine Riboside' OR 'Riboside, 6-Methylthiopurine' | 1,167 |
| Thiouridine | #47 | 'Thiouridine' OR '4-Thiouridine' OR '4 Thiouridine' | 899 |
| Thionucleosides combine | #48 | #44 OR #45 OR #46 OR #47 | 27,544 |
| combine | #49 | #2 OR #8 OR #17 OR #23 OR #30 OR #43 OR #48 | 408,676 |
| combine | #50 | #1 AND #49 and Articles (Document Types) | 336 |
| Part 3. SciFinder | | | |
| Search terms | | | |
| Hydrogels: Hydrogels, Hydrogel, In Situ Hydrogels, In Situ Hydrogel, Hydrogel, In Situ, Patterned Hydrogels, Patterned Hydrogel. | | | |
| Nucleosides: Nucleosides, Arabinonucleosides, Arabinofuranosyluracil, Clofarabine, Cytarabine, Vidarabine, Arabinonucleosides, Deoxyribonucleosides, Deoxyadenosines, Deoxycytidine, Deoxyguanosine, Deoxyuridine, Dideoxynucleosides, Pentostatin, Thymidine , Deoxyribonucleosides conbine, Purine Nucleosides, Adenosine, Guanosine, Inosine, Tubercidin, Purine Nucleosides, Pyrimidine Nucleosides, Cytidine, Formycins, Thymidine, Tunicamycin, Uridine, Pyrimidine Nucleosides, Ribonucleosides, Adenosine, Cytidine, Dichlororibofuranosylbenzimidazole, Formycins, Guanosine, Inosine, Ribavirin, Showdomycin, Toyocamycin, Tubercidin, Uridine, Ribonucleosides, Thionucleosides, Azathioprine, Thioinosine, Thiouridine, Thionucleosides. | | | |
|  | | | |
