# Supplementary material for: Developing a machine learning model for accurate nucleoside hydrogels prediction based on descriptors
Source: Nat Commun. 2024 Mar 23;15:2603. doi: 10.1038/s41467-024-46866-9 (PMC10960799; doi:10.1038/s41467-024-46866-9)
Supplement: Supplementary file 4 — Supplementary Data 1 [file 41467_2024_46866_MOESM4_ESM.pdf]

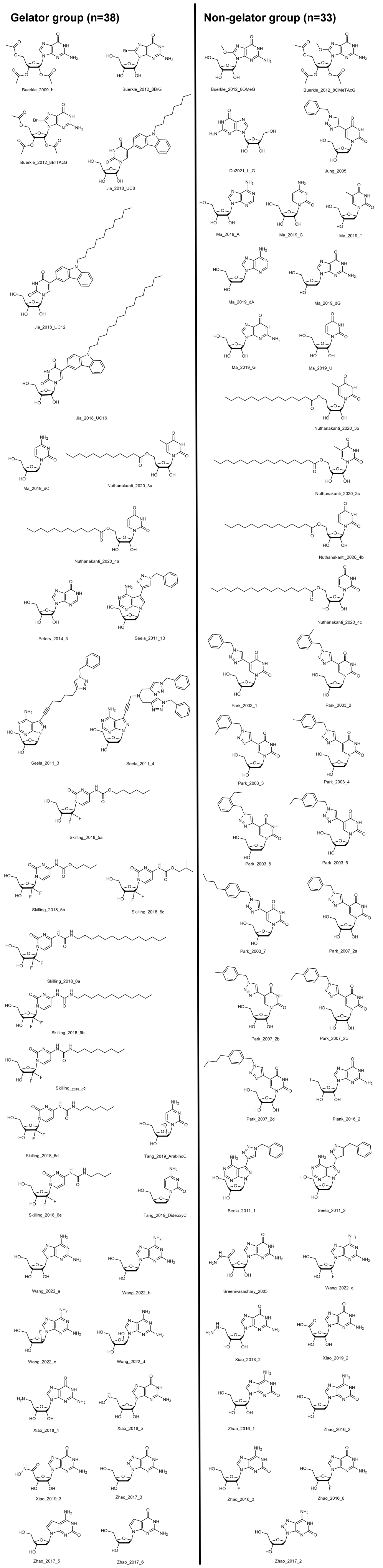
Gelator group (n=38)
Non-gelator group (n=33)
Buerkle_2009_b
Buerkle_2012_8BrG
Buerkle_2012_8BrTAcG
Jia_2018_UC8
Jia_2018_UC12
Jia_2018_UC16
Ma_2019_dC
Nuthanakanti_2020_3a
Nuthanakanti_2020_4a
Peters_2014_3
Seela_2011_13
Seela_2011_3
Seela_2011_4
Skilling_2018_5a
Skilling_2018_5b
Skilling_2018_5c
Skilling_2018_6a
Skilling_2018_6b
Skilling_2018_6c
Skilling_2018_6d
Tang_2019_ArabinoC
Skilling_2018_6e
Tang_2019_DideoxyC
Wang_2022_a
Wang_2022_b
Wang_2022_c
Wang_2022_d
Xiao_2018_4
Xiao_2018_5
Xiao_2019_3
Zhao_2017_3
Zhao_2017_5
Zhao_2017_6
Buerkle_2012_8OMeG
Buerkle_2012_8OMeTAcG
Du2021_L_G
Jung_2005
Ma_2019_A
Ma_2019_C
Ma_2019_T
Ma_2019_dA
Ma_2019_dG
Ma_2019_G
Ma_2019_U
Nuthanakanti_2020_3b
Nuthanakanti_2020_3c
Nuthanakanti_2020_4b
Nuthanakanti_2020_4c
Park_2003_1
Park_2003_2
Park_2003_3
Park_2003_4
Park_2003_5
Park_2003_6
Park_2003_7
Park_2007_2a
Park_2007_2b
Park_2007_2c
Park_2007_2d
Plank_2016_2
Seela_2011_1
Seela_2011_2
Sreenivasachary_2005
Wang_2022_e
Xiao_2018_2
Xiao_2019_2
Zhao_2016_1
Zhao_2016_2
Zhao_2016_3
Zhao_2016_6
Zhao_2017_2
